# Effect of the early diastolic blood pressure response to the head-up tilt test on the recurrence of benign paroxysmal positional vertigo

**DOI:** 10.1371/journal.pone.0301800

**Published:** 2024-05-02

**Authors:** Guil Rhim, Moon Jung Kim

**Affiliations:** 1 Department of Otorhinolaryngology, One Otorhinolaryngology Clinic, Paju, Republic of Korea; 2 Department of Laboratory Medicine, Myunggok Medical Research Center, Konyang University College of Medicine, Daejeon, Republic of Korea; Tehran University of Medical Sciences, ISLAMIC REPUBLIC OF IRAN

## Abstract

**Background:**

Otolith organ acts complementarily with the autonomic nervous system to maintain blood pressure. However, the effect of blood pressure variability in the autonomic nervous system on otolith organ has not yet been determined. This study aimed to verify the hypothesis that blood pressure variability in the autonomic nervous system affects the recurrence of benign paroxysmal positional vertigo (BPPV), which is the most common disease of the vestibular organs, by using the head-up tilt test (HUTT).

**Methods:**

This study included 432 patients diagnosed with idiopathic BPPV. The follow-up period for all patients was 12 months. Age, sex, hypertension, diabetes and recurrence were analyzed. The HUTT parameters were divided into a group of patients whose average diastolic blood pressure increased in the upright position compared to supine position during the HUTT (DBP_1_) and a group of patients whose average diastolic blood pressure decreased in the upright position compared to supine position during the HUTT (DBP_2_). Model selection, general loglinear analysis, and logit loglinear analysis were performed using a hierarchically progressing loglinear analysis.

**Results:**

In summary, the group with increased average diastolic blood pressure (DBP_1_) showed a higher tendency for BPPV recurrence compared to the group with decreased diastolic blood pressure (DBP_2_) in the upright position during the HUTT, although the difference was not statistically significant (p = 0.080). However, in males, the DBP_1_ group demonstrated a significantly higher recurrence rate of BPPV than the DBP_2_ group during the HUTT (95% CI, -20.021 to -16.200; p < 0.001).

**Conclusions:**

It is presumed that poor autonomic nervous system response through vestibulosympathetic reflex maintains elevated diastolic blood pressure in the upright position during the HUTT. This variability is assumed to affect the recurrence of BPPV.

## Introduction

The autonomic nervous system regulates involuntary physiological responses, including digestion, respiration, pulse, and blood pressure. The vestibulosympathetic reflexes (VSRs) and baroreceptor reflexes are involved in the maintenance of blood pressure during involuntary physiological responses. In the vestibular organs, signals stimulated by postural changes are transmitted to the nucleus tractus solitarius and the rostral ventrolateral medulla via the vestibular nucleus complex through VSRs. Signals that enter the nucleus tractus solitarius maintain blood pressure through neurogenic and humoral control mechanisms in the rostral ventrolateral medulla [1]. Many studies have been conducted on the relationship between baroreceptors and blood pressure [2, 3], but studies on the relationship between VSR and blood pressure are relatively few. VSRs differ from responses triggered by the unloading of cardiovascular receptors, such as baroreceptors and cardiopulmonary receptors, because they can be elicited before a change in blood distribution occurs in the body. Differences in the expression of VSRs in humans and other animals have also been described [4–6]. Therefore, studies on the vestibular organs or VSRs related to blood pressure are limited. However, the sympathetic impulses are grouped in pulse synchronous bursts occurring preferentially during transient reductions of blood pressure [7]. Vestibular nerve stimulation increased baroreflex-driven MSNA, as well as the diastolic blood pressure threshold at which bursts of MSNA occurred [8]. When the vestibular evoked myogenic potential (VEMP) response was absent, the diastolic blood pressure dropped significantly at 1 min of active standing [9]. Furthermore, orthostatic hypotension (OH) accompanying benign paroxysmal positional vertigo (BPPV) affects the recurrence of BPPV [10], and utricular dysfunction is associated with OH [11]. The current study hypothesized that if otolith organ acts complementarily with the autonomic nervous system to maintain blood pressure, blood pressure variability may affect otolith organ. Therefore, this study aimed to verify the hypothesis that diastolic blood pressure variability affects the recurrence of BPPV by using the head-up tilt test (HUTT), which is method for examining the autonomic nervous system.

## Methods

### Study subjects

This study included patients with idiopathic BPPV who visited a primary otorhinolaryngology clinic from September 2020 to April 2022 with dizziness as the cardinal symptom. When clinical findings suggestive of BPPV were observed during history-taking and physical examination using Frenzel goggles, the following tests were conducted, and diagnosis and treatment were performed by a single physician.

### Test protocol

**Fig 1:**
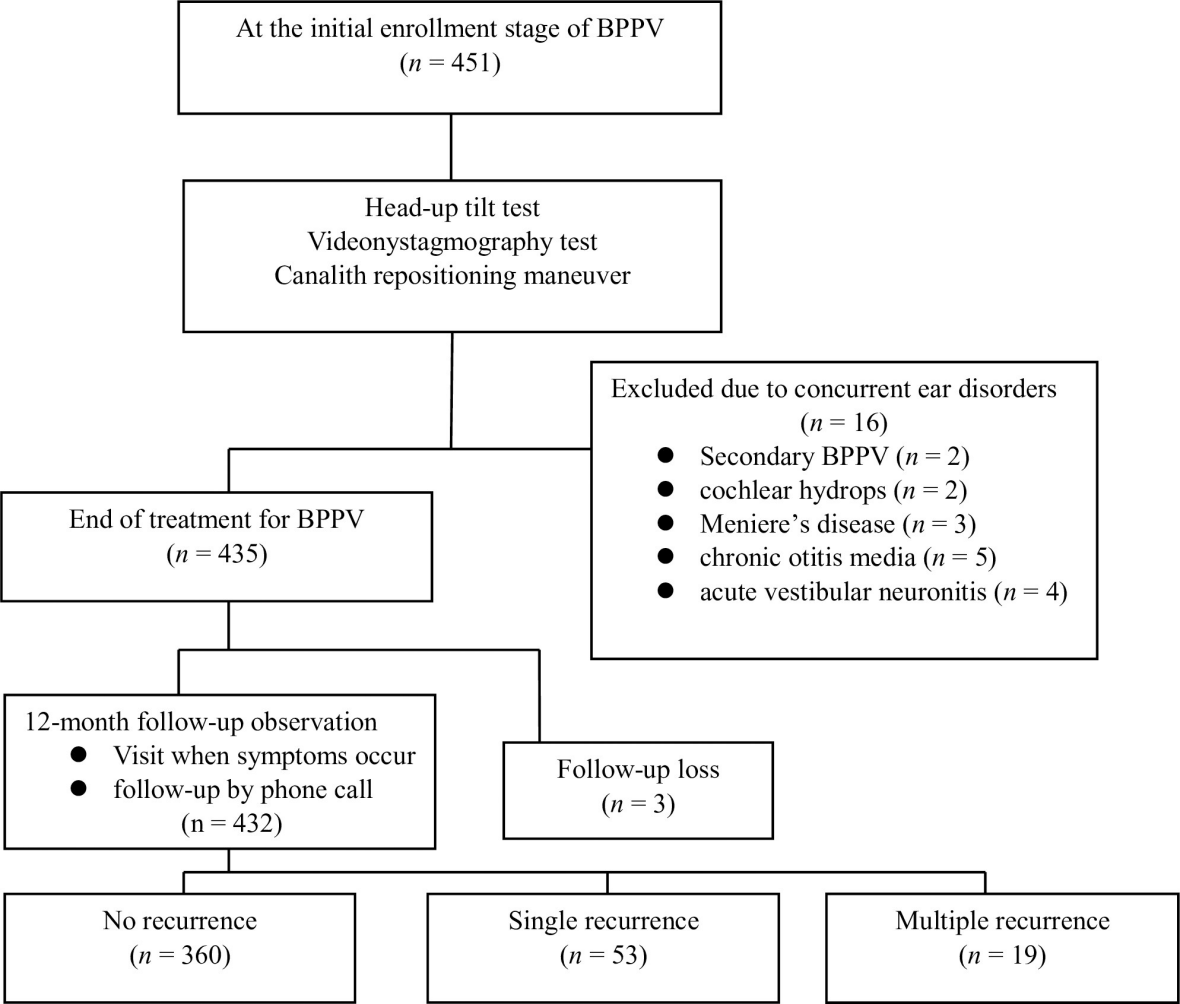
Flow chart of study.

Patients were prohibited from consuming alcohol within 12 h of the test. Drugs for high blood pressure and diabetes, excluding stabilizers, were taken as usual. After resting for 15 min in a quiet, comfortable room, the patient was laid down on a test table with a footplate and restrained by using two Velcro straps placed around the legs and waist. For the sequence of the test, blood pressure and pulse rate were measured twice by using automated cuff measurements in the right arm with an interval of 1 min after resting for 3 min in a lying position. Thereafter, the table was raised to 70° for 30 s, and the blood pressure and pulse rate were measured three times at 1 min intervals. If the patient experienced severe dizziness during the test, the test was immediately discontinued. Subsequently, pure-tone audiometry and video nystagmography tests were performed. Patients diagnosed with BPPV underwent the canalith repositioning maneuver according to the type of BPPV. The canalith repositioning maneuver was performed twice a week, and the treatment was terminated when the symptoms disappeared and the nystagmus disappeared when it was examined using video Frentzel goggles. Patients were instructed to return to the clinic immediately if they suspected BPPV recurrence, and at the one-year mark, direct patient telephone calls were made to ensure the accuracy of the recurrence data. Recurrence was diagnosed as the occurrence of symptoms one month after the end of the treatment. Nystagmus was confirmed using video Frentzel goggles, and treatment was performed again (Fig 1).

### Patients data and variables

During the study period, 451 patients were diagnosed with BPPV. A total of 432 patients were included in this study, excluding 2 with secondary BPPV, 2 with cochlear hydrops, 3 with Ménière’s disease, 5 with chronic otitis media, 4 with acute vestibular neuritis, and 3 who were not followed up. The follow-up period for all patients was 12 months, and age, sex, hypertension, diabetes, and recurrence were assessed using the patients’ medical records and questionnaires. This study was approved by the Institutional Review Board of Myongji Hospital (No. 2023-04-016: Approve date. May 9, 2023). The data was collected for research purposes on May 12, 2023. Written consent was obtained from all patients in our study. Authors had no access to information that could identify individual participants during or after data collection and all data were fully anonymized prior to the study.

The patients were classified into two groups according to age (age < 60 years [AGE_1_] and age ≥ 60 years [AGE_2_]) and into two groups with regard to hypertension and diabetes status (patients who have no disease [HIBP_1_, DM_1_] and patients who are taking drugs [HIBP_2_, DM_2_]). With regard to recurrence, the patients were classified into a group with no recurrence (RECUR_1_), a group with one recurrence in 12 months (RECUR_2_), and a group with two or more recurrences in 12 months (RECUR_3_).

Classification according to variations in blood pressure and pulse rate followed the criteria of the Bárány Society and the International Study Classification for Syncope [12, 13]. The criteria for blood pressure and pulse rate according to each classification were organized and compared with the normal reactions in HUTTs. In the current study, the patients were classified into a group in which the average diastolic blood pressure that was measured thrice in the upright position during the HUTT is equal to or higher than the average of the diastolic blood pressure that was measured twice in the supine position during the HUTT (DBP_1_) and a group in which the average diastolic blood pressure in the upright position is lower than the average of the diastolic blood pressure in the supine position during the HUTT (DBP_2_). Patients with bradycardia or tachycardia were excluded from the HUTT.

### Statistical analysis

Model selection, general loglinear analysis, and logit loglinear analysis were performed using a hierarchically progressing loglinear analysis.

K-way and higher-order effects were checked using hierarchical loglinear analysis for model selection to identify if the quadratic interaction model was meaningful with Sig. = 0.820 at K = 3 and with Sig. = 0.000 at K = 2. The model that was necessary for the analysis was completed using backward elimination statistics.

The Pearson value for the test of independence was Sig. = 0.568. The goodness of fit of the probability model using Poisson distribution was good, and the likelihood ratio was Sig. = 0.514 for the general loglinear analysis.

The logit loglinear analysis model was used. Recurrence was set as the dependent variable, and the remaining variables were applied as explanatory variables.

The Pearson value for the test of independence was Sig. = 0.617. The goodness of fit of the probability model using multinomial distributions was good, and the likelihood ratio was Sig. = 0.421 for the logit loglinear analysis.

Statistical analyses were performed using IBM SPSS ver. 19.0 (IBM Corp., Armonk, IL, USA). *P* < 0.05 was considered statistically significant.

## Results

The age of the 432 participants was 50.60 ± 16.24 years (mean ± SD); furthermore, 310 (71.8%) were female, and 287 (66.4%) were less than 60 years old (Table 1).

**Table 1:** Characteristics of the no recurrence, single recurrence, and multiple recurrence groups in BPPV.

Recurrence	Sex		Age		Hypertension		Diabetics		DBP	
	Female	Male	<60	>60	-	+	-	+	1	2
No	251	109	239	121	262	98	313	47	246	114
Single	42	11	34	19	35	18	45	8	37	16
Multiple	17	2	14	5	15	4	19	2	17	2
**TOTAL (n = 432)**	310	122	287	145	312	120	375	57	300	132

DBP, group with increased (DBP_1_) or decreased (DBP_2_) average diastolic blood pressure upon standing on the head-up tilt test.

A total of 53 patients (12.3%) had one recurrence, and 19 patients (4.4%) had two or more recurrences. The recurrence rate was 16.6% (72/432). There were 300 patients (69.4%) in the DBP_1_ group and 132 patients (30.5%) in the DBP_2_ group. There were 120 patients (27.7%) with hypertension and 57 patients (13.2%) with diabetes.

The associative relationships among recurrence_1,2,3_, hypertension_1,2_, diabetes_1,2_, age_1,2_, and DBP_1,2_ groups were analyzed (Table 2).

**Table 2:** Associative relationships among variables in the general loglinear model.

			95% CI	
Variable	Estimate	Sig.	Lower Bound	Upper Bound
HIBP_1_–DM_1_	1.675	**.000**	.985	2.364
HIBP_1_ –AGE_1_	2.172	**.000**	1.666	2.678
RECUR_1_–DBP_1_	-1.333	**.080** ^**a**^	-2.825	.158
RECUR_2_–DBP_1_	-1.221	**.133** ^**a**^	-2.812	.370
RECUR_3_–DBP_1_	0	.	.	.

RECUR_1_, no recurrence; RECUR_2_, single recurrence; RECUR_3_ multiple recurrence; HIBP_1_, no hypertension; DM_1_, no diabetes; AGE_1_, age < 60 years; DBP_1_, group with elevated average diastolic blood pressure upon standing on the head-up tilt test. Bold values indicate statistical significance (p < 0.05). ^a^ The trend of p *-*values is meaningful.

Relatively high frequencies were expected in the HIBP_1_–DM_1_ and HIBP_1_–AGE_1_ combinations (estimate: 1.675, 2.172). Patients without high blood pressure had a statistically significant tendency to have no diabetes (95% confidence interval [CI], 0.985–2.364; p < 0.001). This also means that the ratio of patients aged <60 years without hypertension was significantly higher than that of patients aged ≥60 years without hypertension (95% CI, 1.666–2.678; p < 0.001).

The RECUR_1_–DBP_1_ and RECUR_2_–DBP_1_ combinations are expected to have relatively lower frequencies than the RECUR_3_–DBP_1_ combination (estimate: -1.333, -1.221). The DBP_1_ group had a high recurrence rate, whereas the DBP_2_ group had a low recurrence rate (95% CI, -2.825 to 0.158; p = 0.080; 95% CI, -2.812 to 0.370; p = 0.133).

Table 3 shows the results of the logit loglinear analysis conducted using hypertension_1,2_, diabetes_1,2_, age_1,2_, and DBP_1,2_ as independent variables, with recurrence_1,2,3_ determined as a dependent variable for males and females.

**Table 3:** Causal relationships using recurrence as a dependent variable in the logit loglinear model.

		Estimate		Sig.		95% CI			
Variables						Lower Bound		Upper Bound	
Independent	Dependent	Male	Female	Male	Female	Male	Female	Male	Female
	RECUR_1_	-18.111	-1.195	**.000**	.119	-20.021	-2.697	-16.200	.306
DBP_1_	RECUR_2_	.000	-1.258	.	.127	.	-2.873	.	.357
	RECUR_3_	0	0	.	.	.	.	.	.

RECUR_1_, no recurrence; RECUR_2_, single recurrence; RECUR_3_, multiple recurrence; DBP_1_, group with elevated average diastolic blood pressure upon standing on the head-up tilt test. Bold values indicate statistical significance (p < 0.05).

The RECUR_1_–DBP_1_ combination is expected to have a very low frequency compared with the RECUR_2_–DBP_1_ and RECUR_3_–DBP_1_ combinations in males (estimate: -18.111). In males, the above information indicates that the ratio of the DBP_1_ group in the group without recurrence was significantly lower than that in the DBP_1_ group in the one-time recurrence group and the ratio in the DBP_1_ group in the multiple recurrence group (95% CI, -20.021 to -16.200; p < 0.001). Similarly, in females, the DBP_1_ group showed a high recurrence rate, whereas the DBP_2_ group showed a low recurrence rate; however, the frequency value (estimate: -1.195, -1.258) or p -value in females had a lower statistical significance than that in males.

## Discussion

The vestibular organ is an important regulator of autonomic nervous system activities and is involved in the posture-related regulation of blood pressure [9]. In experiments involving baroreceptor-denervated animals, vestibular afferent activation causes various and complex cardiovascular changes in different parts of the body. Various patterns of changes in blood flow are shown at the same time, such as increased blood pressure in the upper limbs, decreased blood pressure in the lower limbs, and small changes in the blood pressure of the internal organs [14]. According to an experiment in which rats with sino-aortic baroreceptor denervation were observed for seven months, the average blood pressure was observed to be similar to that of the normal group.

However, the blood pressure variability showed very large differences, and the interbeat interval variability was also very large. Nevertheless, the overall cardiovascular system was maintained very well [2]. Another study reported that the electrical stimulation of the vestibular system on one side in rats with normal baroreceptors resulted in a blood pressure reduction of at least 20 mmHg [15]. Head-down rotation (HDR) experiments engaging the otolith organ in human participants led to an increase in MSNA and caused calf vasoconstriction; however, cardiac output, arterial blood pressure, and mean cerebral blood flow velocities did not change [5, 6].

VSR experiments related to age showed that HDR performance during simultaneous orthostatic stress increased total MSNA further in young subjects but not in older subjects. Older subjects demonstrated significant hypotension during HDR. They concluded that the VSR elicits a diverse pattern of sympathetic outflow that results in heterogeneous vascular responses in humans and that these responses are significantly attenuated in older humans.

Human and animal experiments have shown that the purpose of blood pressure regulation according to posture, position, and external temperature changes is to maintain blood pressure stability. Maintaining stable blood pressure with low variability for the integrated regulation of the blood vessels, heart, and muscles is the role of the baroreceptor and VSR. According to the findings of several studies on normal responses, in the upright position, the diastolic blood pressure and systolic blood pressure slightly increases and decreases, respectively, before reaching a stable state within 1–2 min [16, 17].

According to the criteria of the Bárány Society and the International Study Classification for Syncope [12, 13], diastolic blood pressure is less variable than systolic blood pressure. Andrei et al. [8], described that Vestibular nerve stimulation significantly increased baroreflex-driven MSNA, as well as the diastolic blood pressure threshold. This agrees with data from Kienbaum et al. [7], who also reported the number of sympathetic bursts and the threshold for occurrence of bursts were reproducible in all subjects. Aoki et al. [9] classified the otolith organ according to VEMP responses. They reported that when the VEMP response was absent in men, the diastolic blood pressure dropped significantly compared with other groups at 1 min of active standing.

In the current study, the men in the DBP_1_ group had a significantly higher BPPV recurrence rate than those in the DBP_2_ group. This is probably because the sympathetic nervous system is already excited because of BPPV. Therefore, the reaction of the cardiovascular system to postural changes, namely, limiting the increase in blood pressure and maintaining stability, is considered a normal reaction of the autonomic nervous system. The group with elevated blood pressure in the upright position during the HUTT had high blood pressure variability and was unable to maintain stable blood pressure owing to a poor autonomic nervous system through the VSR, which later affected the recurrence of BPPV.

Although it is not certain, it can be assumed that it is affected by the autonomic nervous system rather than the short reflex response. Reflex is a reaction that occurs in a few seconds, and the parameters for DBP_1_ and DBP_2_ in this study include the average blood pressure for 2 min in the supine position and the average blood pressure for 3 min in the upright position.

The current study showed that the absence of hypertension or diabetes had a significantly higher trend than the presence of hypertension without diabetes (Tables 2); this finding is similar to those of studies involving the general population. The prevalence of hypertension is 40%–60% higher in people with type 2 diabetes between 45–70 years of age than in people without diabetes [18]. Given that 95% of all patients with diabetes have type 2 diabetes, diabetes and hypertension are very closely related with each other. In this study, 120 patients (27.8%) had hypertension, similar to the prevalence of hypertension in adults in developed countries (28.5%) [19]. As shown in Table 2, the number of cases in which patients aged less than 60 years old had no hypertension was significantly larger than that in which patients aged at least 60 years old had hypertension. This is consistent with the results of another study that indicated that the prevalence of hypertension increases with age [20].

A study on how blood pressure variability such as hypertension or hypotension affects the capillaries of the vestibular organ was conducted by Sakagami et al. [21]. The blood-labyrinth barrier (BLB) of the vestibular organ is similar to the blood–brain barrier (BBB) of the brain and is presumably affected similarly by blood pressure variability. Many studies have been conducted on the BBB and hypertension.

Among them, the hypothesis model of Setiadi et al. [22] is interesting. According to this model, damage to the BBB causes autonomic dysfunction. Owing to hypertension, circulating pro-inflammatory cytokines and angiotensin II from the periphery act on the angiotensin II type 1 receptor on the BBB endothelium, thus causing the disruption to tight junction proteins and occludin. Circulating leukocytes access the brain, differentiate into functional microglia, release pro-inflammatory cytokines, and cause neuroinflammation and autonomic dysfunction.

According to Chen et al. [23], the serum levels of interleukin-1β (IL-1β), soluble intercellular adhesion molecule-1(sICAM-1), and soluble vascular adhesion protein-1 (sVAP-1) were significantly higher in patients with BPPV than in controls [23]. Cai et al. [24] indicated that serum levels of macrophage migration inhibitory factor in patients with BPPV were higher than those in controls. Furthermore, patients with BPPV recurrence had higher levels of migration inhibitory factor than patients with no BPPV recurrence. Güçlütürk et al. [25] showed that the total antioxidant levels were lower in the BPPV group than in the control group. After performing the Epley repositioning maneuver in the vertigo group, a statistically significant decrease in IL-1β levels was observed at the first and third months of follow-up.

Given the hypothesis that blood pressure variability affects the recurrence of BPPV, it can be assumed that blood pressure variability injures the BLB of the vestibular organ, affects the destruction of barrier proteins and the migration and diffusion of pro-inflammatory cytokines to the perilymph, and increases BLB receptors, such as sICAM-1 and sVAP-1. Therefore, the effect on the generation and maintenance of otoconia by IL-1β and leukocyte infiltration may increase the recurrence of BPPV.

This study is a preliminary investigation analyzing the impact of changes in diastolic blood pressure using the HUTT on the recurrence of BPPV, applying experimental research observing blood pressure changes using VSR stimulation to clinical conditions. In addition, an independent cohort study is needed to confirm if the autonomic nervous system as a risk factor. In this study, all variables including age, high blood pressure, and diabetes were used as confounding variables. The advantage of this statistical method is that it solves the problem of data loss in a specific layer when there are too many disturbances to be controlled regardless of the reference level. Furthermore, it is easy to calculate the result for the joint effect of one or more factors (Table 2). This method also facilitates the easy understanding of the interaction effect between the independent variables. However, the statistical method used in this study is suitable for examining the associations or dependencies between categorical variables, where one categorical variable acts as the dependent variable, and a model is constructed accordingly. Continuous variables cannot be used in this statistical method, and the inability to incorporate variables such as vitamin D levels that may affect blood pressure is a limitation of this study. It is anticipated that future research will need to consider other variables to address this limitation.

In hospitals, it is possible that patients’ nervousness affects blood pressure during the HUTT. The blood pressure measured in this manner may have had sufficient bias. Some patients in this study may have OH, and such results may have affected the measurement of diastolic blood pressure, thus causing bias. However, the Q–Q plot in this study was normally distributed, and the bias was not significant (S1 File).

This study investigated the response of the initial 5 min of the HUTT, and it is estimated that the influence of the VSR mainly affected the conclusion of this study. In a normal response to the HUTT, elevated diastolic blood pressure is normalized within 1–2 min, but it is presumed that poor autonomic nervous system response through VSR maintains elevated diastolic blood pressure in the upright position during the HUTT. This variability is assumed to affect the recurrence of BPPV. This study contributes to a better understanding of the involvement of the autonomic nervous system in the development of BPPV. These findings have important implications for the prevention and treatment of BPPV.

## Conclusions

This study examined the relationship between diastolic blood pressure variability and BPPV recurrence using the HUTT. Results revealed that increased diastolic blood pressure in the upright position during the HUTT was associated with higher BPPV recurrence rates, suggesting a role for poor autonomic nervous system responses. Understanding these mechanisms may inform targeted interventions to reduce BPPV recurrence and improve patient outcomes.

## Supporting information

S1 File Statistical methods and results. (PDF)

S2 File The data used in this study. (PDF)
