# Supplementary material for: Effect of the early diastolic blood pressure response to the head-up tilt test on the recurrence of benign paroxysmal positional vertigo
Source: PLoS One. 2024 May 2;19(5):e0301800. doi: 10.1371/journal.pone.0301800 (PMC11065201; doi:10.1371/journal.pone.0301800)
Supplement: S1 File — (PDF) [file pone.0301800.s001.pdf]

```
GENLOG Recur1yr123 HIBP DM Age2 DPaverage
  /MODEL=POISSON
  /PRINT=FREQ RESID ADJRESID ZRESID DEV ESTIM CORR COV
  /PLOT=RESID(ADJRESID) NORMPROB(ADJRESID)
  /CRITERIA=CIN(95) ITERATE(20) CONVERGE(0.001) DELTA(.5)
  /DESIGN Recur1yr123 HIBP DM Age2 DPaverage HIBP*Recur1yr123 Age2*DM
DM*DPaverage DM*Recur1yr123 DM*HIBP Age2*HIBP DPaverage*HIBP Age2*DPaverage
Age2*Recur1yr123 DPaverage*Recur1yr123.
```

General Loglinear

Notes

Output Created		14:4월:202319시 38분 41초
Comments		
Input	Data	C:\Users\guzi9\OneDrive\바탕화면\자율\result\ex3.sav
	Active Dataset	DataSet1
	Filter	<none>
	Weight	<none>
	Split File	<none>
	N of Rows in Working Data File	432
Missing Value Handling	Definition of Missing	User-defined missing values are treated as missing.
	Cases Used	Statistics are based on all cases with valid data for all variables in the model.

Syntax		GENLOG Recur1yr123 HIBP DM Age2 DPaverage /MODEL=POISSON /PRINT=FREQ RESID ADJRESID ZRESID DEV ESTIM CORR COV /PLOT=RESID(ADJRESID) NORMPROB(ADJRESID) /CRITERIA=CIN(95) ITERATE(20) CONVERGE(0.001) DELTA(.5) /DESIGN Recur1yr123 HIBP DM Age2 DPaverage HIBP*Recur1yr123 Age2*DM DM*DPaverage DM*Recur1yr123 DM*HIBP Age2*HIBP DPaverage*HIBP Age2*DPaverage Age2*Recur1yr123 DPaverage*Recur1yr123.
Resources	Processor Time	00 00:00:01.719
	Elapsed Time	00 00:00:03.119

[DataSet1] C:\Users\guzi9\OneDrive\바탕 화면\자율\result\ex3.sav

Data Information

		N
Cases	Valid	432
	Missing	0
	Weighted Valid	432
Cells	Defined Cells	48
	Structural Zeros	0
	Sampling Zeros	14
Categories	Recur1yr123	3
	HIBP	2
	DM	2
	Age2	2
	DPaverage	2

Convergence Information[b,c]

Maximum Number of Iterations	20
Converge Tolerance	.00100
Final Maximum Absolute Difference	.00070[a]
Final Maximum Relative Difference	.02695
Number of Iterations	8

a. The iteration converged because the maximum absolute changes of parameter estimates is less than the specified convergence criterion.

b. Model: Poisson

c. Design: Constant + Recur1yr123 + HIBP + DM + Age2 + DPaverage + Recur1yr123 * HIBP + DM * Age2 + DM * DPaverage + Recur1yr123 * DM + HIBP * DM + HIBP * Age2 + HIBP * DPaverage + Age2 * DPaverage + Recur1yr123 * Age2 + Recur1yr123 * DPaverage

Goodness-of-Fit Tests[a,b]

	Value	df	Sig.
Likelihood Ratio	26.080	27	.514
Pearson Chi-Square	25.110	27	.568

a. Model: Poisson

b. Design: Constant + Recur1yr123 + HIBP + DM + Age2 + DPaverage + Recur1yr123 * HIBP + DM * Age2 + DM * DPaverage + Recur1yr123 * DM + HIBP * DM + HIBP * Age2 + HIBP * DPaverage + Age2 * DPaverage + Recur1yr123 * Age2 + Recur1yr123 * DPaverage

Cell Counts and Residuals[a,b]

					Observed		Expected	
Recur1yr123	HIBP	DM	Age2	DPaverage	Count	%	Count	%
1.00	1.00	1.00	1.00	1.00	149	34.5%	148.413	34.4%
				2.00	51	11.8%	51.003	11.8%

			2.00	1.00	29	6.7%	30.419	7.0%
				2.00	14	3.2%	16.195	3.7%
		2.00	1.00	1.00	8	1.9%	7.800	1.8%
				2.00	4	.9%	3.615	.8%
			2.00	1.00	3	.7%	2.652	.6%
				2.00	4	.9%	1.904	.4%
	2.00	1.00	1.00	1.00	13	3.0%	14.484	3.4%
				2.00	8	1.9%	6.982	1.6%
			2.00	1.00	30	6.9%	26.050	6.0%
				2.00	19	4.4%	19.455	4.5%
		2.00	1.00	1.00	3	.7%	4.063	.9%
				2.00	3	.7%	2.641	.6%
			2.00	1.00	11	2.5%	12.120	2.8%
				2.00	11	2.5%	12.206	2.8%
2.00	1.00	1.00	1.00	1.00	23	5.3%	20.624	4.8%
				2.00	5	1.2%	6.333	1.5%
			2.00	1.00	3	.7%	3.964	.9%
				2.00	4	.9%	1.886	.4%
		2.00	1.00	1.00	0	.0%	1.132	.3%
				2.00	0	.0%	.469	.1%
			2.00	1.00	0	.0%	.361	.1%
				2.00	0	.0%	.232	.1%
	2.00	1.00	1.00	1.00	1	.2%	2.874	.7%
				2.00	2	.5%	1.238	.3%
			2.00	1.00	4	.9%	4.847	1.1%
				2.00	3	.7%	3.235	.7%
		2.00	1.00	1.00	3	.7%	.842	.2%
				2.00	0	.0%	.489	.1%
			2.00	1.00	3	.7%	2.356	.5%
				2.00	2	.5%	2.120	.5%
3.00	1.00	1.00	1.00	1.00	11	2.5%	10.978	2.5%
				2.00	1	.2%	.994	.2%
			2.00	1.00	3	.7%	1.922	.4%
				2.00	0	.0%	.270	.1%
		2.00	1.00	1.00	0	.0%	.570	.1%
				2.00	0	.0%	.070	.0%
			2.00	1.00	0	.0%	.165	.0%
				2.00	0	.0%	.031	.0%
	2.00	1.00	1.00	1.00	1	.2%	.956	.2%

				2.00	0	.0%	.122	.0%
			2.00	1.00	0	.0%	1.469	.3%
				2.00	1	.2%	.289	.1%
		2.00	1.00	1.00	1	.2%	.265	.1%
				2.00	0	.0%	.045	.0%
			2.00	1.00	1	.2%	.675	.2%
				2.00	0	.0%	.179	.0%

Cell Counts and Residuals[a,b]

Recur1yr123	HIBP	DM	Age2	DPaverage	Residual	Standardized Residual	Adjusted Residual	Deviance
1.00	1.00	1.00	1.00	1.00	.587	.048	.192	.048
				2.00	-.003	.000	-.001	.000
			2.00	1.00	-1.419	-.257	-.587	-.259
				2.00	-2.195	-.545	-1.017	-.558
		2.00	1.00	1.00	.200	.072	.115	.071
				2.00	.385	.203	.268	.199
			2.00	1.00	.348	.214	.272	.209
				2.00	2.096	1.519	1.837	1.322
	2.00	1.00	1.00	1.00	-1.484	-.390	-.699	-.397
				2.00	1.018	.385	.559	.376
			2.00	1.00	3.950	.774	1.648	.755
				2.00	-.455	-.103	-.201	-.103
		2.00	1.00	1.00	-1.063	-.527	-.728	-.553
				2.00	.359	.221	.280	.216
			2.00	1.00	-1.120	-.322	-.581	-.327
				2.00	-1.206	-.345	-.628	-.351
2.00	1.00	1.00	1.00	1.00	2.376	.523	1.178	.514
				2.00	-1.333	-.530	-.833	-.550
			2.00	1.00	-.964	-.484	-.688	-.506
				2.00	2.114	1.540	1.885	1.337
		2.00	1.00	1.00	-1.132	-1.064	-1.251	-1.064
				2.00	-.469	-.685	-.743	-.685
			2.00	1.00	-.361	-.601	-.644	-.601
				2.00	-.232	-.481	-.506	-.481
	2.00	1.00	1.00	1.00	-1.874	-1.105	-1.467	-1.279
				2.00	.762	.685	.797	.628
			2.00	1.00	-.847	-.385	-.565	-.397
				2.00	-.235	-.130	-.174	-.132
		2.00	1.00	1.00	2.158	2.352	2.690	1.819

				2.00	-.489	-.699	-.764	-.699
			2.00	1.00	.644	.420	.557	.403
				2.00	-.120	-.082	-.107	-.083
3.00	1.00	1.00	1.00	1.00	.022	.007	.019	.007
				2.00	.006	.006	.009	.006
			2.00	1.00	1.078	.778	1.286	.718
				2.00	-.270	-.519	-.585	-.519
		2.00	1.00	1.00	-.570	-.755	-.963	-.755
				2.00	-.070	-.264	-.275	-.264
			2.00	1.00	-.165	-.407	-.442	-.407
				2.00	-.031	-.177	-.181	-.177
	2.00	1.00	1.00	1.00	.044	.045	.060	.044
				2.00	-.122	-.349	-.371	-.349
			2.00	1.00	-1.469	-1.212	-1.809	-1.212
				2.00	.711	1.322	1.503	1.030
		2.00	1.00	1.00	.735	1.429	1.615	1.089
				2.00	-.045	-.213	-.220	-.213
			2.00	1.00	.325	.396	.523	.369
				2.00	-.179	-.423	-.468	-.423

a. Model: Poisson

b. Design: Constant + Recur1yr123 + HIBP + DM + Age2 + DPaverage + Recur1yr123 * HIBP + DM * Age2 + DM * DPaverage + Recur1yr123 * DM + HIBP * DM + HIBP * Age2 + HIBP * DPaverage + Age2 * DPaverage + Recur1yr123 * Age2 + Recur1yr123 * DPaverage

Parameter Estimates[b,c]

Parameter	Estimate	Std. Error	Z	Sig.	95% Confidence Interval Lower Bound	95% Confidence Interval Upper Bound
Constant	-1.720	1.005	-1.712	.087	-3.689	.249
[Recur1yr123 = 1.00]	4.222	.991	4.258	.000	2.279	6.165
[Recur1yr123 = 2.00]	2.471	1.059	2.334	.020	.396	4.546
[Recur1yr123 = 3.00]	0[a]	.	.	.	.	.
[HIBP = 1.00]	-1.744	.764	-2.283	.022	-3.242	-.247
[HIBP = 2.00]	0[a]	.	.	.	.	.
[DM = 1.00]	.479	.846	.566	.571	-1.179	2.137
[DM = 2.00]	0[a]	.	.	.	.	.
[Age2 = 1.00]	-1.373	.695	-1.975	.048	-2.736	-.010

[Age2 = 2.00]	0[a]	.	.	.	.	.
[DPaverage = 1.00]	1.326	.799	1.660	.097	-.240	2.893
[DPaverage = 2.00]	0[a]	.	.	.	.	.
[Recur1yr123 = 1.00] * [HIBP = 1.00]	-.114	.672	-.169	.866	-1.431	1.204
[Recur1yr123 = 1.00] * [HIBP = 2.00]	0[a]	.	.	.	.	.
[Recur1yr123 = 2.00] * [HIBP = 1.00]	-.470	.744	-.631	.528	-1.928	.988
[Recur1yr123 = 2.00] * [HIBP = 2.00]	0[a]	.	.	.	.	.
[Recur1yr123 = 3.00] * [HIBP = 1.00]	0[a]	.	.	.	.	.
[Recur1yr123 = 3.00] * [HIBP = 2.00]	0[a]	.	.	.	.	.
[DM = 1.00] * [Age2 = 1.00]	.506	.349	1.451	.147	-.177	1.189
[DM = 1.00] * [Age2 = 2.00]	0[a]	.	.	.	.	.
[DM = 2.00] * [Age2 = 1.00]	0[a]	.	.	.	.	.
[DM = 2.00] * [Age2 = 2.00]	0[a]	.	.	.	.	.
[DM = 1.00] * [DPaverage = 1.00]	.299	.316	.947	.343	-.320	.918
[DM = 1.00] * [DPaverage = 2.00]	0[a]	.	.	.	.	.
[DM = 2.00] * [DPaverage = 1.00]	0[a]	.	.	.	.	.
[DM = 2.00] * [DPaverage = 2.00]	0[a]	.	.	.	.	.
[Recur1yr123 = 1.00] * [DM = 1.00]	-.013	.811	-.016	.987	-1.602	1.577
[Recur1yr123 = 1.00] * [DM = 2.00]	0[a]	.	.	.	.	.
[Recur1yr123 = 2.00] * [DM = 1.00]	-.056	.894	-.063	.950	-1.808	1.695
[Recur1yr123 = 2.00] * [DM = 2.00]	0[a]	.	.	.	.	.
[Recur1yr123 = 3.00] * [DM = 1.00]	0[a]	.	.	.	.	.
[Recur1yr123 = 3.00] * [DM = 2.00]	0[a]	.	.	.	.	.
[HIBP = 1.00] * [DM = 1.00]	1.675	.352	4.760	.000	.985	2.364
[HIBP = 1.00] * [DM = 2.00]	0[a]	.	.	.	.	.
[HIBP = 2.00] * [DM = 1.00]	0[a]	.	.	.	.	.
[HIBP = 2.00] * [DM = 2.00]	0[a]	.	.	.	.	.

[HIBP = 1.00] * [Age2 = 1.00]	2.172	.258	8.417	.000	1.666	2.678
[HIBP = 1.00] * [Age2 = 2.00]	0[a]	.	.	.	.	.
[HIBP = 2.00] * [Age2 = 1.00]	0[a]	.	.	.	.	.
[HIBP = 2.00] * [Age2 = 2.00]	0[a]	.	.	.	.	.
[HIBP = 1.00] * [DPaverage = 1.00]	.338	.271	1.247	.212	-.193	.870
[HIBP = 1.00] * [DPaverage = 2.00]	0[a]	.	.	.	.	.
[HIBP = 2.00] * [DPaverage = 1.00]	0[a]	.	.	.	.	.
[HIBP = 2.00] * [DPaverage = 2.00]	0[a]	.	.	.	.	.
[Age2 = 1.00] * [DPaverage = 1.00]	.438	.251	1.744	.081	-.054	.930
[Age2 = 1.00] * [DPaverage = 2.00]	0[a]	.	.	.	.	.
[Age2 = 2.00] * [DPaverage = 1.00]	0[a]	.	.	.	.	.
[Age2 = 2.00] * [DPaverage = 2.00]	0[a]	.	.	.	.	.
[Recur1yr123 = 1.00] * [Age2 = 1.00]	-.158	.605	-.261	.794	-1.343	1.028
[Recur1yr123 = 1.00] * [Age2 = 2.00]	0[a]	.	.	.	.	.
[Recur1yr123 = 2.00] * [Age2 = 1.00]	-.094	.679	-.138	.890	-1.425	1.238
[Recur1yr123 = 2.00] * [Age2 = 2.00]	0[a]	.	.	.	.	.
[Recur1yr123 = 3.00] * [Age2 = 1.00]	0[a]	.	.	.	.	.
[Recur1yr123 = 3.00] * [Age2 = 2.00]	0[a]	.	.	.	.	.
[Recur1yr123 = 1.00] * [DPaverage = 1.00]	-1.333	.761	-1.752	.080	-2.825	.158
[Recur1yr123 = 1.00] * [DPaverage = 2.00]	0[a]	.	.	.	.	.
[Recur1yr123 = 2.00] * [DPaverage = 1.00]	-1.221	.812	-1.504	.133	-2.812	.370

[Recur1yr123 = 2.00] * [DPaverage = 2.00]	0[a]	.	.	.	.	.
[Recur1yr123 = 3.00] * [DPaverage = 1.00]	0[a]	.	.	.	.	.
[Recur1yr123 = 3.00] * [DPaverage = 2.00]	0[a]	.	.	.	.	.

a. This parameter is set to zero because it is redundant.

b. Model: Poisson

c. Design: Constant + Recur1yr123 + HIBP + DM + Age2 + DPaverage + Recur1yr123 * HIBP + DM * Age2 + DM * DPaverage + Recur1yr123 * DM + HIBP * DM + HIBP * Age2 + HIBP * DPaverage + Age2 * DPaverage + Recur1yr123 * Age2 + Recur1yr123 * DPaverage

Correlations of Parameter Estimates[a,b,c]

	Constant	[Recur1yr123 = 1.00]	[Recur1yr123 = 2.00]	[HIBP = 1.00]	[DM = 1.00]
Constant	1	-.971	-.911	-.141	-.555
[Recur1yr123 = 1.00]	-.971	1	.911	.119	.512
[Recur1yr123 = 2.00]	-.911	.911	1	.119	.480
[HIBP = 1.00]	-.141	.119	.119	1	-.201
[DM = 1.00]	-.555	.512	.480	-.201	1
[Age2 = 1.00]	-.148	.118	.110	-.398	-.034
[DPaverage = 1.00]	-.632	.595	.557	-.027	.041
[Recur1yr123 = 1.00] * [HIBP = 1.00]	.129	-.133	-.121	-.863	.236
[Recur1yr123 = 2.00] * [HIBP = 1.00]	.115	-.116	-.121	-.772	.213
[DM = 1.00] * [Age2 = 1.00]	.051	-.007	-.004	.217	-.095
[DM = 1.00] * [DPaverage = 1.00]	.154	-.051	-.044	.098	-.287
[Recur1yr123 = 1.00] * [DM = 1.00]	.527	-.542	-.487	.213	-.943
[Recur1yr123 = 2.00] * [DM = 1.00]	.478	-.472	-.555	.183	-.857
[HIBP = 1.00] * [DM = 1.00]	.044	-.005	-.019	-.386	-.085
[HIBP = 1.00] * [Age2 = 1.00]	.020	-.003	-.006	-.171	.041
[HIBP = 1.00] * [DPaverage = 1.00]	.036	-.014	-.020	-.284	.084
[Age2 = 1.00] * [DPaverage = 1.00]	.041	-.016	-.012	.134	.027

[Recur1yr123 = 1.00] * [Age2 = 1.00]	.128	-.132	-.119	.362	.066
[Recur1yr123 = 2.00] * [Age2 = 1.00]	.115	-.113	-.136	.318	.059
[Recur1yr123 = 1.00] * [DPaverage = 1.00]	.592	-.609	-.562	.036	.032
[Recur1yr123 = 2.00] * [DPaverage = 1.00]	.558	-.564	-.585	.028	.030

Correlations of Parameter Estimates[a,b,c]

	[Age2 = 1.00]	[DPaverage = 1.00]	[Recur1yr123 = 1.00] * [HIBP = 1.00]	[Recur1yr123 = 2.00] * [HIBP = 1.00]
Constant	-.148	-.632	.129	.115
[Recur1yr123 = 1.00]	.118	.595	-.133	-.116
[Recur1yr123 = 2.00]	.110	.557	-.121	-.121
[HIBP = 1.00]	-.398	-.027	-.863	-.772
[DM = 1.00]	-.034	.041	.236	.213
[Age2 = 1.00]	1	-.040	.357	.326
[DPaverage = 1.00]	-.040	1	.033	.031
[Recur1yr123 = 1.00] * [HIBP = 1.00]	.357	.033	1	.863
[Recur1yr123 = 2.00] * [HIBP = 1.00]	.326	.031	.863	1
[DM = 1.00] * [Age2 = 1.00]	-.358	.028	-.004	-.003
[DM = 1.00] * [DPaverage = 1.00]	.053	-.257	-.024	-.020
[Recur1yr123 = 1.00] * [DM = 1.00]	.056	.006	-.251	-.216
[Recur1yr123 = 2.00] * [DM = 1.00]	.053	.008	-.217	-.252
[HIBP = 1.00] * [DM = 1.00]	.183	.022	-.002	-.005
[HIBP = 1.00] * [Age2 = 1.00]	-.139	.012	.011	-.009
[HIBP = 1.00] * [DPaverage = 1.00]	.141	-.059	.092	.072
[Age2 = 1.00] * [DPaverage = 1.00]	-.291	-.069	-.040	-.032
[Recur1yr123 = 1.00] * [Age2 = 1.00]	-.853	.047	-.420	-.362
[Recur1yr123 = 2.00] * [Age2 = 1.00]	-.766	.043	-.356	-.428

[Recur1yr123 = 1.00] * [DPaverage = 1.00]	.048	-.931	-.043	-.037
[Recur1yr123 = 2.00] * [DPaverage = 1.00]	.046	-.878	-.037	-.044

Correlations of Parameter Estimates[a,b,c]

	[DM = 1.00] * [Age2 = 1.00]	[DM = 1.00] * [DPaverage = 1.00]	[Recur1yr123 = 1.00] * [DM = 1.00]	[Recur1yr123 = 2.00] * [DM = 1.00]
Constant	.051	.154	.527	.478
[Recur1yr123 = 1.00]	-.007	-.051	-.542	-.472
[Recur1yr123 = 2.00]	-.004	-.044	-.487	-.555
[HIBP = 1.00]	.217	.098	.213	.183
[DM = 1.00]	-.095	-.287	-.943	-.857
[Age2 = 1.00]	-.358	.053	.056	.053
[DPaverage = 1.00]	.028	-.257	.006	.008
[Recur1yr123 = 1.00] * [HIBP = 1.00]	-.004	-.024	-.251	-.217
[Recur1yr123 = 2.00] * [HIBP = 1.00]	-.003	-.020	-.216	-.252
[DM = 1.00] * [Age2 = 1.00]	1	-.088	.013	.007
[DM = 1.00] * [DPaverage = 1.00]	-.088	1	.095	.077
[Recur1yr123 = 1.00] * [DM = 1.00]	.013	.095	1	.869
[Recur1yr123 = 2.00] * [DM = 1.00]	.007	.077	.869	1
[HIBP = 1.00] * [DM = 1.00]	-.466	-.056	.010	.034
[HIBP = 1.00] * [Age2 = 1.00]	-.250	.033	-.004	-.005
[HIBP = 1.00] * [DPaverage = 1.00]	.046	-.268	-.024	-.020
[Age2 = 1.00] * [DPaverage = 1.00]	-.042	-.072	-.007	-.006
[Recur1yr123 = 1.00] * [Age2 = 1.00]	.000	-.007	-.071	-.061
[Recur1yr123 = 2.00] * [Age2 = 1.00]	.003	-.006	-.060	-.072
[Recur1yr123 = 1.00] * [DPaverage = 1.00]	.000	-.003	-.034	-.030
[Recur1yr123 = 2.00] * [DPaverage = 1.00]	.000	.000	-.031	-.036

Correlations of Parameter Estimates[a,b,c]

	[HIBP = 1.00] * [DM = 1.00]	[HIBP = 1.00] * [Age2 = 1.00]	[HIBP = 1.00] * [DPaverage = 1.00]	[Age2 = 1.00] * [DPaverage = 1.00]
Constant	.044	.020	.036	.041
[Recur1yr123 = 1.00]	-.005	-.003	-.014	-.016
[Recur1yr123 = 2.00]	-.019	-.006	-.020	-.012
[HIBP = 1.00]	-.386	-.171	-.284	.134
[DM = 1.00]	-.085	.041	.084	.027
[Age2 = 1.00]	.183	-.139	.141	-.291
[DPaverage = 1.00]	.022	.012	-.059	-.069
[Recur1yr123 = 1.00] * [HIBP = 1.00]	-.002	.011	.092	-.040
[Recur1yr123 = 2.00] * [HIBP = 1.00]	-.005	-.009	.072	-.032
[DM = 1.00] * [Age2 = 1.00]	-.466	-.250	.046	-.042
[DM = 1.00] * [DPaverage = 1.00]	-.056	.033	-.268	-.072
[Recur1yr123 = 1.00] * [DM = 1.00]	.010	-.004	-.024	-.007
[Recur1yr123 = 2.00] * [DM = 1.00]	.034	-.005	-.020	-.006
[HIBP = 1.00] * [DM = 1.00]	1	.050	-.034	.029
[HIBP = 1.00] * [Age2 = 1.00]	.050	1	-.070	-.027
[HIBP = 1.00] * [DPaverage = 1.00]	-.034	-.070	1	-.448
[Age2 = 1.00] * [DPaverage = 1.00]	.029	-.027	-.448	1
[Recur1yr123 = 1.00] * [Age2 = 1.00]	-.001	.003	-.041	.092
[Recur1yr123 = 2.00] * [Age2 = 1.00]	-.004	.035	-.035	.073
[Recur1yr123 = 1.00] * [DPaverage = 1.00]	.000	-.001	.002	.004
[Recur1yr123 = 2.00] * [DPaverage = 1.00]	-.002	-.003	.021	.001

Correlations of Parameter Estimates[a,b,c]

	[Recur1yr123 = 1.00] * [Age2 = 1.00]	[Recur1yr123 = 2.00] * [Age2 = 1.00]	[Recur1yr123 = 1.00] * [DPaverage = 1.00]	[Recur1yr123 = 2.00] * [DPaverage = 1.00]

Constant	.128	.115	.592	.558
[Recur1yr123 = 1.00]	-.132	-.113	-.609	-.564
[Recur1yr123 = 2.00]	-.119	-.136	-.562	-.585
[HIBP = 1.00]	.362	.318	.036	.028
[DM = 1.00]	.066	.059	.032	.030
[Age2 = 1.00]	-.853	-.766	.048	.046
[DPaverage = 1.00]	.047	.043	-.931	-.878
[Recur1yr123 = 1.00] * [HIBP = 1.00]	-.420	-.356	-.043	-.037
[Recur1yr123 = 2.00] * [HIBP = 1.00]	-.362	-.428	-.037	-.044
[DM = 1.00] * [Age2 = 1.00]	.000	.003	.000	.000
[DM = 1.00] * [DPaverage = 1.00]	-.007	-.006	-.003	.000
[Recur1yr123 = 1.00] * [DM = 1.00]	-.071	-.060	-.034	-.031
[Recur1yr123 = 2.00] * [DM = 1.00]	-.061	-.072	-.030	-.036
[HIBP = 1.00] * [DM = 1.00]	-.001	-.004	.000	-.002
[HIBP = 1.00] * [Age2 = 1.00]	.003	.035	-.001	-.003
[HIBP = 1.00] * [DPaverage = 1.00]	-.041	-.035	.002	.021
[Age2 = 1.00] * [DPaverage = 1.00]	.092	.073	.004	.001
[Recur1yr123 = 1.00] * [Age2 = 1.00]	1	.850	-.058	-.052
[Recur1yr123 = 2.00] * [Age2 = 1.00]	.850	1	-.049	-.062
[Recur1yr123 = 1.00] * [DPaverage = 1.00]	-.058	-.049	1	.916
[Recur1yr123 = 2.00] * [DPaverage = 1.00]	-.052	-.062	.916	1

a. Model: Poisson

b. Design: Constant + Recur1yr123 + HIBP + DM + Age2 + DPaverage + Recur1yr123 * HIBP + DM * Age2 + DM * DPaverage + Recur1yr123 * DM + HIBP * DM + HIBP * Age2 + HIBP * DPaverage + Age2 * DPaverage + Recur1yr123 * Age2 + Recur1yr123 * DPaverage

c. Redundant parameters are not displayed.

Covariances of Parameter Estimates[a,b,c]

	Constant	[Recur1yr123 = 1.00]	[Recur1yr123 = 2.00]	[HIBP = 1.00]	[DM = 1.00]
Constant	1.010	-.968	-.969	-.108	-.472
[Recur1yr123 = 1.00]	-.968	.983	.956	.090	.429
[Recur1yr123 = 2.00]	-.969	.956	1.121	.096	.430
[HIBP = 1.00]	-.108	.090	.096	.584	-.130
[DM = 1.00]	-.472	.429	.430	-.130	.716
[Age2 = 1.00]	-.103	.081	.081	-.211	-.020
[DPaverage = 1.00]	-.508	.472	.471	-.016	.028
[Recur1yr123 = 1.00] * [HIBP = 1.00]	.087	-.089	-.086	-.443	.134
[Recur1yr123 = 2.00] * [HIBP = 1.00]	.086	-.085	-.096	-.439	.134
[DM = 1.00] * [Age2 = 1.00]	.018	-.002	-.002	.058	-.028
[DM = 1.00] * [DPaverage = 1.00]	.049	-.016	-.015	.024	-.077
[Recur1yr123 = 1.00] * [DM = 1.00]	.429	-.436	-.418	.132	-.647
[Recur1yr123 = 2.00] * [DM = 1.00]	.430	-.418	-.525	.125	-.648
[HIBP = 1.00] * [DM = 1.00]	.016	-.002	-.007	-.104	-.025
[HIBP = 1.00] * [Age2 = 1.00]	.005	-.001	-.002	-.034	.009
[HIBP = 1.00] * [DPaverage = 1.00]	.010	-.004	-.006	-.059	.019
[Age2 = 1.00] * [DPaverage = 1.00]	.010	-.004	-.003	.026	.006
[Recur1yr123 = 1.00] * [Age2 = 1.00]	.078	-.079	-.076	.167	.034
[Recur1yr123 = 2.00] * [Age2 = 1.00]	.079	-.076	-.098	.165	.034
[Recur1yr123 = 1.00] * [DPaverage = 1.00]	.453	-.460	-.453	.021	.021
[Recur1yr123 = 2.00] * [DPaverage = 1.00]	.455	-.454	-.503	.018	.020

Covariances of Parameter Estimates[a,b,c]

	[Age2 = 1.00]	[DPaverage = 1.00]	[Recur1yr123 = 1.00] * [HIBP = 1.00]	[Recur1yr123 = 2.00] * [HIBP = 1.00]
Constant	-.103	-.508	.087	.086
[Recur1yr123 = 1.00]	.081	.472	-.089	-.085

[Recur1yr123 = 2.00]	.081	.471	-.086	-.096
[HIBP = 1.00]	-.211	-.016	-.443	-.439
[DM = 1.00]	-.020	.028	.134	.134
[Age2 = 1.00]	.483	-.022	.167	.169
[DPaverage = 1.00]	-.022	.639	.018	.018
[Recur1yr123 = 1.00] * [HIBP = 1.00]	.167	.018	.452	.432
[Recur1yr123 = 2.00] * [HIBP = 1.00]	.169	.018	.432	.553
[DM = 1.00] * [Age2 = 1.00]	-.087	.008	-.001	-.001
[DM = 1.00] * [DPaverage = 1.00]	.012	-.065	-.005	-.005
[Recur1yr123 = 1.00] * [DM = 1.00]	.031	.004	-.137	-.130
[Recur1yr123 = 2.00] * [DM = 1.00]	.033	.006	-.130	-.168
[HIBP = 1.00] * [DM = 1.00]	.045	.006	.000	-.001
[HIBP = 1.00] * [Age2 = 1.00]	-.025	.003	.002	-.002
[HIBP = 1.00] * [DPaverage = 1.00]	.027	-.013	.017	.015
[Age2 = 1.00] * [DPaverage = 1.00]	-.051	-.014	-.007	-.006
[Recur1yr123 = 1.00] * [Age2 = 1.00]	-.359	.023	-.171	-.163
[Recur1yr123 = 2.00] * [Age2 = 1.00]	-.362	.023	-.163	-.216
[Recur1yr123 = 1.00] * [DPaverage = 1.00]	.025	-.566	-.022	-.021
[Recur1yr123 = 2.00] * [DPaverage = 1.00]	.026	-.569	-.020	-.027

Covariances of Parameter Estimates[a,b,c]

	[DM = 1.00] * [Age2 = 1.00]	[DM = 1.00] * [DPaverage = 1.00]	[Recur1yr123 = 1.00] * [DM = 1.00]	[Recur1yr123 = 2.00] * [DM = 1.00]
Constant	.018	.049	.429	.430
[Recur1yr123 = 1.00]	-.002	-.016	-.436	-.418
[Recur1yr123 = 2.00]	-.002	-.015	-.418	-.525
[HIBP = 1.00]	.058	.024	.132	.125
[DM = 1.00]	-.028	-.077	-.647	-.648
[Age2 = 1.00]	-.087	.012	.031	.033
[DPaverage = 1.00]	.008	-.065	.004	.006

[Recur1yr123 = 1.00] * [HIBP = 1.00]	-.001	-.005	-.137	-.130
[Recur1yr123 = 2.00] * [HIBP = 1.00]	-.001	-.005	-.130	-.168
[DM = 1.00] * [Age2 = 1.00]	.122	-.010	.004	.002
[DM = 1.00] * [DPaverage = 1.00]	-.010	.100	.024	.022
[Recur1yr123 = 1.00] * [DM = 1.00]	.004	.024	.658	.630
[Recur1yr123 = 2.00] * [DM = 1.00]	.002	.022	.630	.799
[HIBP = 1.00] * [DM = 1.00]	-.057	-.006	.003	.011
[HIBP = 1.00] * [Age2 = 1.00]	-.023	.003	-.001	-.001
[HIBP = 1.00] * [DPaverage = 1.00]	.004	-.023	-.005	-.005
[Age2 = 1.00] * [DPaverage = 1.00]	-.004	-.006	-.001	-.001
[Recur1yr123 = 1.00] * [Age2 = 1.00]	.000	-.001	-.035	-.033
[Recur1yr123 = 2.00] * [Age2 = 1.00]	.001	-.001	-.033	-.044
[Recur1yr123 = 1.00] * [DPaverage = 1.00]	.000	-.001	-.021	-.020
[Recur1yr123 = 2.00] * [DPaverage = 1.00]	.000	.000	-.020	-.026

Covariances of Parameter Estimates[a,b,c]

	[HIBP = 1.00] * [DM = 1.00]	[HIBP = 1.00] * [Age2 = 1.00]	[HIBP = 1.00] * [DPaverage = 1.00]	[Age2 = 1.00] * [DPaverage = 1.00]
Constant	.016	.005	.010	.010
[Recur1yr123 = 1.00]	-.002	-.001	-.004	-.004
[Recur1yr123 = 2.00]	-.007	-.002	-.006	-.003
[HIBP = 1.00]	-.104	-.034	-.059	.026
[DM = 1.00]	-.025	.009	.019	.006
[Age2 = 1.00]	.045	-.025	.027	-.051
[DPaverage = 1.00]	.006	.003	-.013	-.014
[Recur1yr123 = 1.00] * [HIBP = 1.00]	.000	.002	.017	-.007
[Recur1yr123 = 2.00] * [HIBP = 1.00]	-.001	-.002	.015	-.006
[DM = 1.00] * [Age2 = 1.00]	-.057	-.023	.004	-.004

[DM = 1.00] * [DPaverage = 1.00]	-.006	.003	-.023	-.006
[Recur1yr123 = 1.00] * [DM = 1.00]	.003	-.001	-.005	-.001
[Recur1yr123 = 2.00] * [DM = 1.00]	.011	-.001	-.005	-.001
[HIBP = 1.00] * [DM = 1.00]	.124	.005	-.003	.003
[HIBP = 1.00] * [Age2 = 1.00]	.005	.067	-.005	-.002
[HIBP = 1.00] * [DPaverage = 1.00]	-.003	-.005	.074	-.031
[Age2 = 1.00] * [DPaverage = 1.00]	.003	-.002	-.031	.063
[Recur1yr123 = 1.00] * [Age2 = 1.00]	.000	.001	-.007	.014
[Recur1yr123 = 2.00] * [Age2 = 1.00]	-.001	.006	-.006	.012
[Recur1yr123 = 1.00] * [DPaverage = 1.00]	.000	.000	.000	.001
[Recur1yr123 = 2.00] * [DPaverage = 1.00]	.000	-.001	.005	.000

Covariances of Parameter Estimates[a,b,c]

	[Recur1yr123 = 1.00] * [Age2 = 1.00]	[Recur1yr123 = 2.00] * [Age2 = 1.00]	[Recur1yr123 = 1.00] * [DPaverage = 1.00]	[Recur1yr123 = 2.00] * [DPaverage = 1.00]
Constant	.078	.079	.453	.455
[Recur1yr123 = 1.00]	-.079	-.076	-.460	-.454
[Recur1yr123 = 2.00]	-.076	-.098	-.453	-.503
[HIBP = 1.00]	.167	.165	.021	.018
[DM = 1.00]	.034	.034	.021	.020
[Age2 = 1.00]	-.359	-.362	.025	.026
[DPaverage = 1.00]	.023	.023	-.566	-.569
[Recur1yr123 = 1.00] * [HIBP = 1.00]	-.171	-.163	-.022	-.020
[Recur1yr123 = 2.00] * [HIBP = 1.00]	-.163	-.216	-.021	-.027
[DM = 1.00] * [Age2 = 1.00]	.000	.001	.000	.000
[DM = 1.00] * [DPaverage = 1.00]	-.001	-.001	-.001	.000
[Recur1yr123 = 1.00] * [DM = 1.00]	-.035	-.033	-.021	-.020

[Recur1yr123 = 2.00] * [DM = 1.00]	-.033	-.044	-.020	-.026
[HIBP = 1.00] * [DM = 1.00]	.000	-.001	.000	.000
[HIBP = 1.00] * [Age2 = 1.00]	.001	.006	.000	-.001
[HIBP = 1.00] * [DPaverage = 1.00]	-.007	-.006	.000	.005
[Age2 = 1.00] * [DPaverage = 1.00]	.014	.012	.001	.000
[Recur1yr123 = 1.00] * [Age2 = 1.00]	.366	.349	-.027	-.026
[Recur1yr123 = 2.00] * [Age2 = 1.00]	.349	.461	-.025	-.034
[Recur1yr123 = 1.00] * [DPaverage = 1.00]	-.027	-.025	.579	.566
[Recur1yr123 = 2.00] * [DPaverage = 1.00]	-.026	-.034	.566	.659

a. Model: Poisson

b. Design: Constant + Recur1yr123 + HIBP + DM + Age2 + DPaverage + Recur1yr123 * HIBP + DM * Age2 + DM * DPaverage + Recur1yr123 * DM + HIBP * DM + HIBP * Age2 + HIBP * DPaverage + Age2 * DPaverage + Recur1yr123 * Age2 + Recur1yr123 * DPaverage

c. Redundant parameters are not displayed.

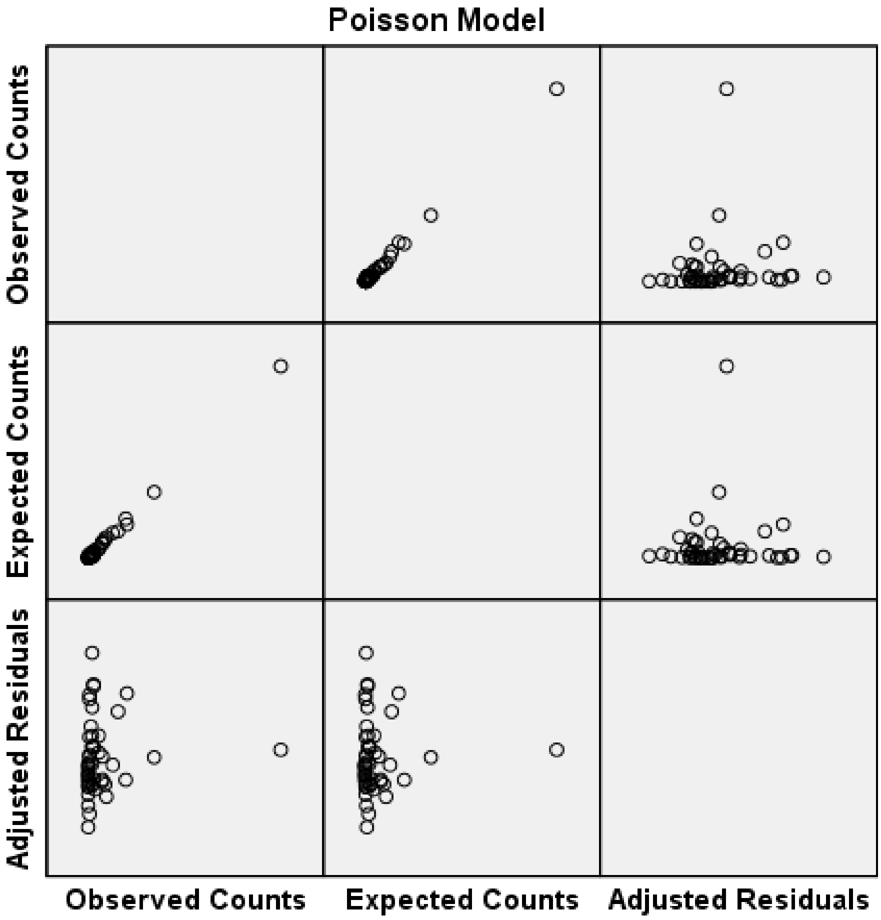
Poisson Model
Observed Counts
Expected Counts
Adjusted Residuals
Observed Counts
Expected Counts
Adjusted Residuals

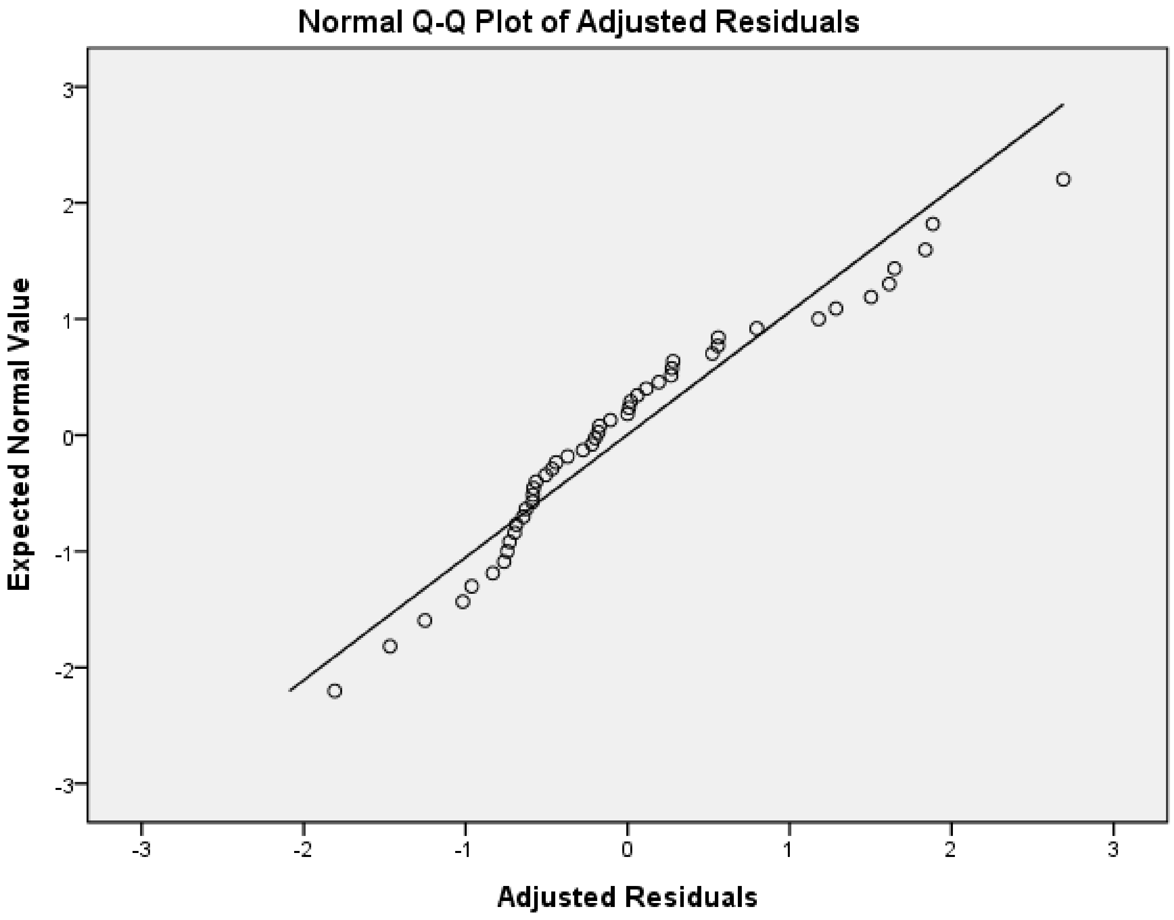
Normal Q-Q Plot of Adjusted Residuals
Expected Normal Value
Adjusted Residuals
3
2
1
0
-1
-2
-3
-3
-2
-1
0
1
2
3

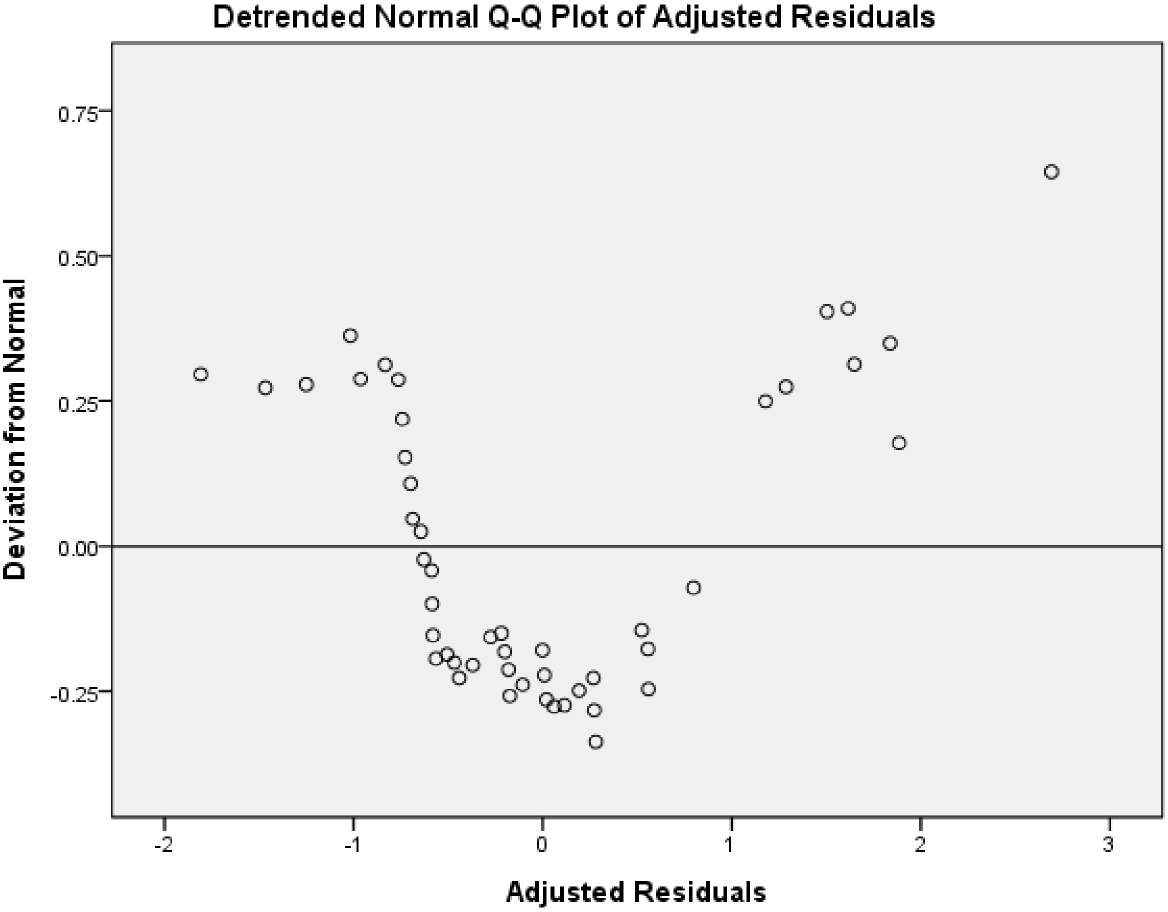


```
USE ALL.
COMPUTE filter_$=(sex = 1).
VARIABLE LABELS filter_$ 'sex = 1 (FILTER)'.
VALUE LABELS filter_$ 0 'Not Selected' 1 'Selected'.
FORMATS filter_$ (f1.0).
FILTER BY filter_$.
EXECUTE.
GENLOG Recur1yr123 HIBP DM Age2 DPaverage
  /MODEL=POISSON
  /PRINT=FREQ RESID ADJRESID ZRESID DEV ESTIM CORR COV
  /PLOT=RESID(ADJRESID) NORMPROB(ADJRESID)
  /CRITERIA=CIN(95) ITERATE(20) CONVERGE(0.001) DELTA(.5)
  /DESIGN Recur1yr123 HIBP DM Age2 DPaverage HIBP*Recur1yr123 Age2*DM
DM*DPaverage DM*Recur1yr123 DM*HIBP Age2*HIBP DPaverage*HIBP Age2*DPaverage
Age2*Recur1yr123 DPaverage*Recur1yr123.
```

General Loglinear male

Notes

Output Created		14:4월:202319시 45분 45초
Comments		
Input	Data	C:\Users\guzi9\OneDrive\바탕 화면\자율\result\ex3.sav
	Active Dataset	DataSet1
	Filter	sex = 1 (FILTER)
	Weight	<none>
	Split File	<none>
	N of Rows in Working Data File	122
Missing Value Handling	Definition of Missing	User-defined missing values are treated as missing.
	Cases Used	Statistics are based on all cases with valid data for all variables in the model.
Syntax		GENLOG Recur1yr123 HIBP DM Age2 DPaverage<br>/MODEL=POISSON<br>/PRINT=FREQ RESID ADJRESID ZRESID DEV ESTIM CORR COV<br>/PLOT=RESID(ADJRESID) NORMPROB(ADJRESID)<br>/CRITERIA=CIN(95) ITERATE(20) CONVERGE(0.001) DELTA(.5)<br>/DESIGN Recur1yr123 HIBP DM Age2 DPaverage HIBP*Recur1yr123 Age2*DM DM*DPaverage DM*Recur1yr123 DM*HIBP Age2*HIBP DPaverage*HIBP Age2*DPaverage Age2*Recur1yr123 DPaverage*Recur1yr123.
Resources	Processor Time	00 00:00:00.641
	Elapsed Time	00 00:00:00.629

```
[DataSet1] C:\Users\guzi9\OneDrive\바탕 화면\자율\result\ex3.sav
```

Data Information

		N
Cases	Valid	122
	Missing	0
	Weighted Valid	122
Cells	Defined Cells	48
	Structural Zeros	0
	Sampling Zeros	28
Categories	Recur1yr123	3
	HIBP	2
	DM	2
	Age2	2
	DPaverage	2

Convergence Information[b,c]

Maximum Number of Iterations	20
Converge Tolerance	.00100
Final Maximum Absolute Difference	1.00000
Final Maximum Relative Difference	.06720
Number of Iterations	20[a]

a. The parameter estimates did not converge before the maximum number of iterations was performed.

b. Model: Poisson

c. Design: Constant + Recur1yr123 + HIBP + DM + Age2 + DPaverage + Recur1yr123 * HIBP + DM * Age2 + DM * DPaverage + Recur1yr123 * DM + HIBP * DM + HIBP * Age2 + HIBP * DPaverage + Age2 * DPaverage + Recur1yr123 * Age2 + Recur1yr123 * DPaverage

Goodness-of-Fit Tests[a,b]

	Value	df	Sig.
Likelihood Ratio	19.279	27	.860

Pearson Chi-Square	17.334	27	.923

a. Model: Poisson

b. Design: Constant + Recur1yr123 + HIBP + DM + Age2 + DPaverage + Recur1yr123 * HIBP + DM * Age2 + DM * DPaverage + Recur1yr123 * DM + HIBP * DM + HIBP * Age2 + HIBP * DPaverage + Age2 * DPaverage + Recur1yr123 * Age2 + Recur1yr123 * DPaverage

Cell Counts and Residuals[a,b]

Recur1yr123	HIBP	DM	Age2	DPaverage	Observed Count	Observed %	Expected Count	Expected %
1.00	1.00	1.00	1.00	1.00	52	42.6%	50.642	41.5%
				2.00	9	7.4%	10.103	8.3%
			2.00	1.00	5	4.1%	6.502	5.3%
				2.00	4	3.3%	3.468	2.8%
		2.00	1.00	1.00	3	2.5%	3.090	2.5%
				2.00	1	.8%	1.390	1.1%
			2.00	1.00	0	.0%	.819	.7%
				2.00	3	2.5%	.986	.8%
	2.00	1.00	1.00	1.00	3	2.5%	4.281	3.5%
				2.00	3	2.5%	2.307	1.9%
			2.00	1.00	7	5.7%	5.201	4.3%
				2.00	7	5.7%	7.495	6.1%
		2.00	1.00	1.00	1	.8%	.987	.8%
				2.00	2	1.6%	1.200	1.0%
			2.00	1.00	3	2.5%	2.478	2.0%
				2.00	6	4.9%	8.051	6.6%
2.00	1.00	1.00	1.00	1.00	8	6.6%	6.633	5.4%
				2.00	0	.0%	.951	.8%
			2.00	1.00	0	.0%	.000	.0%
				2.00	0	.0%	.000	.0%
		2.00	1.00	1.00	0	.0%	.314	.3%
				2.00	0	.0%	.102	.1%
			2.00	1.00	0	.0%	.000	.0%
				2.00	0	.0%	.000	.0%
	2.00	1.00	1.00	1.00	0	.0%	1.741	1.4%
				2.00	2	1.6%	.675	.6%

			2.00	1.00	0	.0%	.000	.0%
				2.00	0	.0%	.000	.0%
		2.00	1.00	1.00	1	.8%	.312	.3%
				2.00	0	.0%	.272	.2%
			2.00	1.00	0	.0%	.000	.0%
				2.00	0	.0%	.000	.0%
3.00	1.00	1.00	1.00	1.00	1	.8%	.571	.5%
				2.00	0	.0%	.000	.0%
			2.00	1.00	0	.0%	.129	.1%
				2.00	0	.0%	.000	.0%
		2.00	1.00	1.00	0	.0%	.204	.2%
				2.00	0	.0%	.000	.0%
			2.00	1.00	0	.0%	.095	.1%
				2.00	0	.0%	.000	.0%
	2.00	1.00	1.00	1.00	0	.0%	.095	.1%
				2.00	0	.0%	.000	.0%
			2.00	1.00	0	.0%	.204	.2%
				2.00	0	.0%	.000	.0%
		2.00	1.00	1.00	0	.0%	.129	.1%
				2.00	0	.0%	.000	.0%
			2.00	1.00	1	.8%	.571	.5%
				2.00	0	.0%	.000	.0%

Cell Counts and Residuals[a,b]

Recur1yr123	HIBP	DM	Age2	DPaverage	Residual	Standardized Residual	Adjusted Residual	Deviance
1.00	1.00	1.00	1.00	1.00	1.358	.191	.978	.190
				2.00	-1.103	-.347	-.924	-.354
			2.00	1.00	-1.502	-.589	-1.333	-.614
				2.00	.532	.285	.513	.279
		2.00	1.00	1.00	-.090	-.051	-.092	-.051
				2.00	-.390	-.331	-.467	-.348
			2.00	1.00	-.819	-.905	-1.165	-.905
				2.00	2.014	2.029	2.710	1.628
	2.00	1.00	1.00	1.00	-1.281	-.619	-1.203	-.655
				2.00	.693	.456	.716	.436
			2.00	1.00	1.799	.789	1.616	.749
				2.00	-.495	-.181	-.427	-.183
		2.00	1.00	1.00	.013	.013	.017	.013

				2.00	.800	.731	1.021	.666
			2.00	1.00	.522	.332	.555	.321
				2.00	-2.051	-.723	-1.837	-.757
2.00	1.00	1.00	1.00	1.00	1.367	.531	1.904	.514
				2.00	-.951	-.975	-1.602	-.975
			2.00	1.00	.000	.000	.000	.000
				2.00	.000	.000	.000	.000
		2.00	1.00	1.00	-.314	-.561	-.735	-.561
				2.00	-.102	-.319	-.352	-.319
			2.00	1.00	.000	.000	.000	.000
				2.00	.000	.000	.000	.000
	2.00	1.00	1.00	1.00	-1.741	-1.320	-2.743	-1.320
				2.00	1.325	1.614	2.330	1.302
			2.00	1.00	.000	.000	.000	.000
				2.00	.000	.000	.000	.000
		2.00	1.00	1.00	.688	1.233	1.608	.977
				2.00	-.272	-.522	-.665	-.522
			2.00	1.00	.000	.000	.000	.000
				2.00	.000	.000	.000	.000
3.00	1.00	1.00	1.00	1.00	.429	.568	1.152	.513
				2.00	.000	.000	.000	.000
			2.00	1.00	-.129	-.359	-.473	-.359
				2.00	.000	.000	.000	.000
		2.00	1.00	1.00	-.204	-.452	-.650	-.452
				2.00	.000	.000	.000	.000
			2.00	1.00	-.095	-.309	-.383	-.309
				2.00	.000	.000	.000	.000
	2.00	1.00	1.00	1.00	-.095	-.309	-.383	-.309
				2.00	.000	.000	.000	.000
			2.00	1.00	-.204	-.452	-.650	-.452
				2.00	.000	.000	.000	.000
		2.00	1.00	1.00	-.129	-.359	-.473	-.359
				2.00	.000	.000	.000	.000
			2.00	1.00	.429	.568	1.152	.513
				2.00	.000	.000	.000	.000

a. Model: Poisson

b. Design: Constant + Recur1yr123 + HIBP + DM + Age2 + DPaverage + Recur1yr123 * HIBP + DM * Age2 + DM * DPaverage + Recur1yr123 * DM + HIBP * DM + HIBP * Age2 + HIBP * DPaverage + Age2 * DPaverage + Recur1yr123 * Age2 + Recur1yr123 * DPaverage

Parameter Estimates[b,c]

Parameter	Estimate	Std. Error	Z	Sig.	95% Confidence Interval	
					Lower Bound	Upper Bound
Constant	-16.441	1852.393	-.009	.993	-3647.065	3614.183
[Recur1yr123 = 1.00]	18.527	1852.393	.010	.992	-3612.097	3649.151
[Recur1yr123 = 2.00]	-1.135	2633.250	.000	1.000	-5162.210	5159.940
[Recur1yr123 = 3.00]	0[a]	.	.	.	.	.
[HIBP = 1.00]	-2.782	1.865	-1.492	.136	-6.437	.872
[HIBP = 2.00]	0[a]	.	.	.	.	.
[DM = 1.00]	-1.841	1.658	-1.110	.267	-5.091	1.409
[DM = 2.00]	0[a]	.	.	.	.	.
[Age2 = 1.00]	-2.470	1.810	-1.365	.172	-6.017	1.077
[Age2 = 2.00]	0[a]	.	.	.	.	.
[DPaverage = 1.00]	15.881	1852.393	.009	.993	-3614.744	3646.505
[DPaverage = 2.00]	0[a]	.	.	.	.	.
[Recur1yr123 = 1.00] * [HIBP = 1.00]	.682	1.808	.377	.706	-2.861	4.225
[Recur1yr123 = 1.00] * [HIBP = 2.00]	0[a]	.	.	.	.	.
[Recur1yr123 = 2.00] * [HIBP = 1.00]	-.451	1.940	-.233	.816	-4.253	3.351
[Recur1yr123 = 2.00] * [HIBP = 2.00]	0[a]	.	.	.	.	.
[Recur1yr123 = 3.00] * [HIBP = 1.00]	0[a]	.	.	.	.	.
[Recur1yr123 = 3.00] * [HIBP = 2.00]	0[a]	.	.	.	.	.
[DM = 1.00] * [Age2 = 1.00]	.725	.647	1.122	.262	-.542	1.993
[DM = 1.00] * [Age2 = 2.00]	0[a]	.	.	.	.	.
[DM = 2.00] * [Age2 = 1.00]	0[a]	.	.	.	.	.

[DM = 2.00] * [Age2 = 2.00]	0[a]	.	.	.	.	.
[DM = 1.00] * [DPaverage = 1.00]	.813	.577	1.408	.159	-.318	1.944
[DM = 1.00] * [DPaverage = 2.00]	0[a]	.	.	.	.	.
[DM = 2.00] * [DPaverage = 1.00]	0[a]	.	.	.	.	.
[DM = 2.00] * [DPaverage = 2.00]	0[a]	.	.	.	.	.
[Recur1yr123 = 1.00] * [DM = 1.00]	1.769	1.612	1.098	.272	-1.389	4.928
[Recur1yr123 = 1.00] * [DM = 2.00]	0[a]	.	.	.	.	.
[Recur1yr123 = 2.00] * [DM = 1.00]	2.022	1.944	1.040	.298	-1.787	5.832
[Recur1yr123 = 2.00] * [DM = 2.00]	0[a]	.	.	.	.	.
[Recur1yr123 = 3.00] * [DM = 1.00]	0[a]	.	.	.	.	.
[Recur1yr123 = 3.00] * [DM = 2.00]	0[a]	.	.	.	.	.
[HIBP = 1.00] * [DM = 1.00]	1.330	.627	2.120	.034	.101	2.559
[HIBP = 1.00] * [DM = 2.00]	0[a]	.	.	.	.	.
[HIBP = 2.00] * [DM = 1.00]	0[a]	.	.	.	.	.
[HIBP = 2.00] * [DM = 2.00]	0[a]	.	.	.	.	.
[HIBP = 1.00] * [Age2 = 1.00]	2.247	.534	4.212	.000	1.202	3.293
[HIBP = 1.00] * [Age2 = 2.00]	0[a]	.	.	.	.	.
[HIBP = 2.00] * [Age2 = 1.00]	0[a]	.	.	.	.	.
[HIBP = 2.00] * [Age2 = 2.00]	0[a]	.	.	.	.	.
[HIBP = 1.00] * [DPaverage = 1.00]	.994	.524	1.897	.058	-.033	2.020
[HIBP = 1.00] * [DPaverage = 2.00]	0[a]	.	.	.	.	.

[HIBP = 2.00] * [DPaverage = 1.00]	0[a]	.	.	.	.	.
[HIBP = 2.00] * [DPaverage = 2.00]	0[a]	.	.	.	.	.
[Age2 = 1.00] * [DPaverage = 1.00]	.984	.527	1.866	.062	-.049	2.016
[Age2 = 1.00] * [DPaverage = 2.00]	0[a]	.	.	.	.	.
[Age2 = 2.00] * [DPaverage = 1.00]	0[a]	.	.	.	.	.
[Age2 = 2.00] * [DPaverage = 2.00]	0[a]	.	.	.	.	.
[Recur1yr123 = 1.00] * [Age2 = 1.00]	.566	1.759	.322	.747	-2.881	4.013
[Recur1yr123 = 1.00] * [Age2 = 2.00]	0[a]	.	.	.	.	.
[Recur1yr123 = 2.00] * [Age2 = 1.00]	18.746	1871.536	.010	.992	-3649.397	3686.888
[Recur1yr123 = 2.00] * [Age2 = 2.00]	0[a]	.	.	.	.	.
[Recur1yr123 = 3.00] * [Age2 = 1.00]	0[a]	.	.	.	.	.
[Recur1yr123 = 3.00] * [Age2 = 2.00]	0[a]	.	.	.	.	.
[Recur1yr123 = 1.00] * [DPaverage = 1.00]	-17.059	1852.394	-.009	.993	-3647.684	3613.565
[Recur1yr123 = 1.00] * [DPaverage = 2.00]	0[a]	.	.	.	.	.
[Recur1yr123 = 2.00] * [DPaverage = 1.00]	-16.729	1852.394	-.009	.993	-3647.354	3613.896
[Recur1yr123 = 2.00] * [DPaverage = 2.00]	0[a]	.	.	.	.	.
[Recur1yr123 = 3.00] * [DPaverage = 1.00]	0[a]	.	.	.	.	.
[Recur1yr123 = 3.00] * [DPaverage = 2.00]	0[a]	.	.	.	.	.

a. This parameter is set to zero because it is redundant.

b. Model: Poisson

c. Design: Constant + Recur1yr123 + HIBP + DM + Age2 + DPaverage + Recur1yr123 * HIBP + DM * Age2 + DM * DPaverage + Recur1yr123 * DM + HIBP * DM + HIBP * Age2 + HIBP * DPaverage + Age2 * DPaverage + Recur1yr123 * Age2 + Recur1yr123 * DPaverage

Correlations of Parameter Estimates[a,b,c]

	Constant	[Recur1yr123 = 1.00]	[Recur1yr123 = 2.00]	[HIBP = 1.00]	[DM = 1.00]
Constant	1	-1.000	-.703	.000	.000
[Recur1yr123 = 1.00]	-1.000	1	.703	.000	.000
[Recur1yr123 = 2.00]	-.703	.703	1	.000	.000
[HIBP = 1.00]	.000	.000	.000	1	-.260
[DM = 1.00]	.000	.000	.000	-.260	1
[Age2 = 1.00]	.000	.000	.000	-.465	-.137
[DPaverage = 1.00]	-1.000	1.000	.703	.000	.000
[Recur1yr123 = 1.00] * [HIBP = 1.00]	.000	.000	.000	-.937	.265
[Recur1yr123 = 2.00] * [HIBP = 1.00]	.000	.000	.000	-.843	.243
[DM = 1.00] * [Age2 = 1.00]	.000	.000	.000	.071	-.047
[DM = 1.00] * [DPaverage = 1.00]	.000	.000	.000	.076	-.280
[Recur1yr123 = 1.00] * [DM = 1.00]	.000	.000	.000	.262	-.965
[Recur1yr123 = 2.00] * [DM = 1.00]	.000	.000	.000	.201	-.776
[HIBP = 1.00] * [DM = 1.00]	.000	.000	.000	-.135	-.042
[HIBP = 1.00] * [Age2 = 1.00]	.000	.000	.000	-.105	.015
[HIBP = 1.00] * [DPaverage = 1.00]	.000	.000	.000	-.245	.070
[Age2 = 1.00] * [DPaverage = 1.00]	.000	.000	.000	.122	.041
[Recur1yr123 = 1.00] * [Age2 = 1.00]	.000	.000	.000	.454	.144
[Recur1yr123 = 2.00] * [Age2 = 1.00]	.000	.000	-.711	.000	.000
[Recur1yr123 = 1.00] * [DPaverage = 1.00]	1.000	-1.000	-.703	.000	.000
[Recur1yr123 = 2.00] * [DPaverage = 1.00]	1.000	-1.000	-.703	.000	.000

Correlations of Parameter Estimates[a,b,c]

	[Age2 = 1.00]	[DPaverage = 1.00]	[Recur1yr123 = 1.00] * [HIBP = 1.00]	[Recur1yr123 = 2.00] * [HIBP = 1.00]
Constant	.000	-1.000	.000	.000

[Recur1yr123 = 1.00]	.000	1.000	.000	.000
[Recur1yr123 = 2.00]	.000	.703	.000	.000
[HIBP = 1.00]	-.465	.000	-.937	-.843
[DM = 1.00]	-.137	.000	.265	.243
[Age2 = 1.00]	1	.000	.453	.427
[DPaverage = 1.00]	.000	1	.000	.000
[Recur1yr123 = 1.00] * [HIBP = 1.00]	.453	.000	1	.911
[Recur1yr123 = 2.00] * [HIBP = 1.00]	.427	.000	.911	1
[DM = 1.00] * [Age2 = 1.00]	-.113	.000	.062	.089
[DM = 1.00] * [DPaverage = 1.00]	.049	.000	-.008	-.002
[Recur1yr123 = 1.00] * [DM = 1.00]	.141	.000	-.268	-.244
[Recur1yr123 = 2.00] * [DM = 1.00]	.138	.000	-.227	-.256
[HIBP = 1.00] * [DM = 1.00]	.055	.000	-.115	-.129
[HIBP = 1.00] * [Age2 = 1.00]	-.077	.000	-.018	-.143
[HIBP = 1.00] * [DPaverage = 1.00]	.122	.000	.131	.094
[Age2 = 1.00] * [DPaverage = 1.00]	-.250	.000	-.053	-.022
[Recur1yr123 = 1.00] * [Age2 = 1.00]	-.935	.000	-.481	-.435
[Recur1yr123 = 2.00] * [Age2 = 1.00]	-.001	.000	.000	.000
[Recur1yr123 = 1.00] * [DPaverage = 1.00]	.000	-1.000	.000	.000
[Recur1yr123 = 2.00] * [DPaverage = 1.00]	.000	-1.000	.000	.000

Correlations of Parameter Estimates[a,b,c]

	[DM = 1.00] * [Age2 = 1.00]	[DM = 1.00] * [DPaverage = 1.00]	[Recur1yr123 = 1.00] * [DM = 1.00]	[Recur1yr123 = 2.00] * [DM = 1.00]
Constant	.000	.000	.000	.000
[Recur1yr123 = 1.00]	.000	.000	.000	.000
[Recur1yr123 = 2.00]	.000	.000	.000	.000
[HIBP = 1.00]	.071	.076	.262	.201
[DM = 1.00]	-.047	-.280	-.965	-.776
[Age2 = 1.00]	-.113	.049	.141	.138

[DPaverage = 1.00]	.000	.000	.000	.000
[Recur1yr123 = 1.00] * [HIBP = 1.00]	.062	-.008	-.268	-.227
[Recur1yr123 = 2.00] * [HIBP = 1.00]	.089	-.002	-.244	-.256
[DM = 1.00] * [Age2 = 1.00]	1	-.182	-.024	-.190
[DM = 1.00] * [DPaverage = 1.00]	-.182	1	.179	.128
[Recur1yr123 = 1.00] * [DM = 1.00]	-.024	.179	1	.799
[Recur1yr123 = 2.00] * [DM = 1.00]	-.190	.128	.799	1
[HIBP = 1.00] * [DM = 1.00]	-.447	-.172	-.018	.070
[HIBP = 1.00] * [Age2 = 1.00]	-.193	.077	.017	.038
[HIBP = 1.00] * [DPaverage = 1.00]	.113	-.206	-.031	-.031
[Age2 = 1.00] * [DPaverage = 1.00]	-.128	-.096	-.014	.009
[Recur1yr123 = 1.00] * [Age2 = 1.00]	-.121	.006	-.141	-.094
[Recur1yr123 = 2.00] * [Age2 = 1.00]	.000	.000	.000	.000
[Recur1yr123 = 1.00] * [DPaverage = 1.00]	.000	.000	.000	.000
[Recur1yr123 = 2.00] * [DPaverage = 1.00]	.000	.000	.000	.000

Correlations of Parameter Estimates[a,b,c]

	[HIBP = 1.00] * [DM = 1.00]	[HIBP = 1.00] * [Age2 = 1.00]	[HIBP = 1.00] * [DPaverage = 1.00]	[Age2 = 1.00] * [DPaverage = 1.00]
Constant	.000	.000	.000	.000
[Recur1yr123 = 1.00]	.000	.000	.000	.000
[Recur1yr123 = 2.00]	.000	.000	.000	.000
[HIBP = 1.00]	-.135	-.105	-.245	.122
[DM = 1.00]	-.042	.015	.070	.041
[Age2 = 1.00]	.055	-.077	.122	-.250
[DPaverage = 1.00]	.000	.000	.000	.000
[Recur1yr123 = 1.00] * [HIBP = 1.00]	-.115	-.018	.131	-.053
[Recur1yr123 = 2.00] * [HIBP = 1.00]	-.129	-.143	.094	-.022

[DM = 1.00] * [Age2 = 1.00]	-.447	-.193	.113	-.128
[DM = 1.00] * [DPaverage = 1.00]	-.172	.077	-.206	-.096
[Recur1yr123 = 1.00] * [DM = 1.00]	-.018	.017	-.031	-.014
[Recur1yr123 = 2.00] * [DM = 1.00]	.070	.038	-.031	.009
[HIBP = 1.00] * [DM = 1.00]	1	-.019	-.109	.094
[HIBP = 1.00] * [Age2 = 1.00]	-.019	1	-.123	-.124
[HIBP = 1.00] * [DPaverage = 1.00]	-.109	-.123	1	-.426
[Age2 = 1.00] * [DPaverage = 1.00]	.094	-.124	-.426	1
[Recur1yr123 = 1.00] * [Age2 = 1.00]	.063	-.029	-.055	.135
[Recur1yr123 = 2.00] * [Age2 = 1.00]	.000	.000	.000	.000
[Recur1yr123 = 1.00] * [DPaverage = 1.00]	.000	.000	.000	.000
[Recur1yr123 = 2.00] * [DPaverage = 1.00]	.000	.000	.000	.000

Correlations of Parameter Estimates[a,b,c]

	[Recur1yr123 = 1.00] * [Age2 = 1.00]	[Recur1yr123 = 2.00] * [Age2 = 1.00]	[Recur1yr123 = 1.00] * [DPaverage = 1.00]	[Recur1yr123 = 2.00] * [DPaverage = 1.00]
Constant	.000	.000	1.000	1.000
[Recur1yr123 = 1.00]	.000	.000	-1.000	-1.000
[Recur1yr123 = 2.00]	.000	-.711	-.703	-.703
[HIBP = 1.00]	.454	.000	.000	.000
[DM = 1.00]	.144	.000	.000	.000
[Age2 = 1.00]	-.935	-.001	.000	.000
[DPaverage = 1.00]	.000	.000	-1.000	-1.000
[Recur1yr123 = 1.00] * [HIBP = 1.00]	-.481	.000	.000	.000
[Recur1yr123 = 2.00] * [HIBP = 1.00]	-.435	.000	.000	.000
[DM = 1.00] * [Age2 = 1.00]	-.121	.000	.000	.000
[DM = 1.00] * [DPaverage = 1.00]	.006	.000	.000	.000

[Recur1yr123 = 1.00] * [DM = 1.00]	-.141	.000	.000	.000
[Recur1yr123 = 2.00] * [DM = 1.00]	-.094	.000	.000	.000
[HIBP = 1.00] * [DM = 1.00]	.063	.000	.000	.000
[HIBP = 1.00] * [Age2 = 1.00]	-.029	.000	.000	.000
[HIBP = 1.00] * [DPaverage = 1.00]	-.055	.000	.000	.000
[Age2 = 1.00] * [DPaverage = 1.00]	.135	.000	.000	.000
[Recur1yr123 = 1.00] * [Age2 = 1.00]	1	.001	.000	.000
[Recur1yr123 = 2.00] * [Age2 = 1.00]	.001	1	.000	.000
[Recur1yr123 = 1.00] * [DPaverage = 1.00]	.000	.000	1	1.000
[Recur1yr123 = 2.00] * [DPaverage = 1.00]	.000	.000	1.000	1

a. Model: Poisson

b. Design: Constant + Recur1yr123 + HIBP + DM + Age2 + DPaverage + Recur1yr123 * HIBP + DM * Age2 + DM * DPaverage + Recur1yr123 * DM + HIBP * DM + HIBP * Age2 + HIBP * DPaverage + Age2 * DPaverage + Recur1yr123 * Age2 + Recur1yr123 * DPaverage

c. Redundant parameters are not displayed.

Covariances of Parameter Estimates[a,b,c]

	Constant	[Recur1yr123 = 1.00]	[Recur1yr123 = 2.00]	[HIBP = 1.00]
Constant	3431360.854	-3431360.832	-3431360.798	-.089
[Recur1yr123 = 1.00]	-3431360.832	3431360.915	3431360.847	.054
[Recur1yr123 = 2.00]	-3431360.798	3431360.847	6934004.553	.114
[HIBP = 1.00]	-.089	.054	.114	3.477
[DM = 1.00]	-.348	.265	.209	-.804
[Age2 = 1.00]	-.183	.142	.046	-1.569
[DPaverage = 1.00]	-3431360.583	3431360.573	3431360.549	-.377
[Recur1yr123 = 1.00] * [HIBP = 1.00]	.077	-.087	-.066	-3.158
[Recur1yr123 = 2.00] * [HIBP = 1.00]	.071	-.086	.013	-3.050

[DM = 1.00] * [Age2 = 1.00]	.009	.028	.161	.086
[DM = 1.00] * [DPaverage = 1.00]	.039	.011	.029	.082
[Recur1yr123 = 1.00] * [DM = 1.00]	.325	-.333	-.278	.786
[Recur1yr123 = 2.00] * [DM = 1.00]	.311	-.311	-1.082	.728
[HIBP = 1.00] * [DM = 1.00]	.006	.023	-.035	-.157
[HIBP = 1.00] * [Age2 = 1.00]	.007	.007	-.021	-.105
[HIBP = 1.00] * [DPaverage = 1.00]	.007	.005	-.011	-.239
[Age2 = 1.00] * [DPaverage = 1.00]	.015	.001	.044	.120
[Recur1yr123 = 1.00] * [Age2 = 1.00]	.163	-.177	-.189	1.488
[Recur1yr123 = 2.00] * [Age2 = 1.00]	.153	-.140	-3502643.029	1.441
[Recur1yr123 = 1.00] * [DPaverage = 1.00]	3431360.538	-3431360.598	-3431360.591	.391
[Recur1yr123 = 2.00] * [DPaverage = 1.00]	3431360.530	-3431360.587	-3431360.829	.328

Covariances of Parameter Estimates[a,b,c]

	[DM = 1.00]	[Age2 = 1.00]	[DPaverage = 1.00]	[Recur1yr123 = 1.00] * [HIBP = 1.00]
Constant	-.348	-.183	-3431360.583	.077
[Recur1yr123 = 1.00]	.265	.142	3431360.573	-.087
[Recur1yr123 = 2.00]	.209	.046	3431360.549	-.066
[HIBP = 1.00]	-.804	-1.569	-.377	-3.158
[DM = 1.00]	2.750	-.410	-.307	.795
[Age2 = 1.00]	-.410	3.276	-.379	1.482
[DPaverage = 1.00]	-.307	-.379	3431361.637	.359
[Recur1yr123 = 1.00] * [HIBP = 1.00]	.795	1.482	.359	3.268
[Recur1yr123 = 2.00] * [HIBP = 1.00]	.782	1.499	.353	3.194
[DM = 1.00] * [Age2 = 1.00]	-.050	-.132	.026	.073
[DM = 1.00] * [DPaverage = 1.00]	-.268	.051	-.057	-.008
[Recur1yr123 = 1.00] * [DM = 1.00]	-2.579	.410	.312	-.780

[Recur1yr123 = 2.00] * [DM = 1.00]	-2.501	.484	.308	-.798
[HIBP = 1.00] * [DM = 1.00]	-.044	.063	.021	-.130
[HIBP = 1.00] * [Age2 = 1.00]	.013	-.074	.013	-.017
[HIBP = 1.00] * [DPaverage = 1.00]	.061	.116	-.010	.124
[Age2 = 1.00] * [DPaverage = 1.00]	.035	-.238	-.022	-.051
[Recur1yr123 = 1.00] * [Age2 = 1.00]	.421	-2.977	.363	-1.528
[Recur1yr123 = 2.00] * [Age2 = 1.00]	.413	-3.045	.386	-1.498
[Recur1yr123 = 1.00] * [DPaverage = 1.00]	.460	.406	-3431361.572	-.412
[Recur1yr123 = 2.00] * [DPaverage = 1.00]	.464	.506	-3431361.561	-.375

Covariances of Parameter Estimates[a,b,c]

	[Recur1yr123 = 2.00] * [HIBP = 1.00]	[DM = 1.00] * [Age2 = 1.00]	[DM = 1.00] * [DPaverage = 1.00]	[Recur1yr123 = 1.00] * [DM = 1.00]
Constant	.071	.009	.039	.325
[Recur1yr123 = 1.00]	-.086	.028	.011	-.333
[Recur1yr123 = 2.00]	.013	.161	.029	-.278
[HIBP = 1.00]	-3.050	.086	.082	.786
[DM = 1.00]	.782	-.050	-.268	-2.579
[Age2 = 1.00]	1.499	-.132	.051	.410
[DPaverage = 1.00]	.353	.026	-.057	.312
[Recur1yr123 = 1.00] * [HIBP = 1.00]	3.194	.073	-.008	-.780
[Recur1yr123 = 2.00] * [HIBP = 1.00]	3.762	.112	-.002	-.764
[DM = 1.00] * [Age2 = 1.00]	.112	.418	-.068	-.025
[DM = 1.00] * [DPaverage = 1.00]	-.002	-.068	.333	.166
[Recur1yr123 = 1.00] * [DM = 1.00]	-.764	-.025	.166	2.597
[Recur1yr123 = 2.00] * [DM = 1.00]	-.967	-.239	.143	2.502
[HIBP = 1.00] * [DM = 1.00]	-.157	-.181	-.062	-.018
[HIBP = 1.00] * [Age2 = 1.00]	-.148	-.067	.024	.015

[HIBP = 1.00] * [DPaverage = 1.00]	.095	.038	-.062	-.026
[Age2 = 1.00] * [DPaverage = 1.00]	-.022	-.044	-.029	-.012
[Recur1yr123 = 1.00] * [Age2 = 1.00]	-1.483	-.137	.006	-.400
[Recur1yr123 = 2.00] * [Age2 = 1.00]	-1.747	-.126	.013	-.385
[Recur1yr123 = 1.00] * [DPaverage = 1.00]	-.400	.031	-.153	-.430
[Recur1yr123 = 2.00] * [DPaverage = 1.00]	-.492	.052	-.163	-.427

Covariances of Parameter Estimates[a,b,c]

	[Recur1yr123 = 2.00] * [DM = 1.00]	[HIBP = 1.00] * [DM = 1.00]	[HIBP = 1.00] * [Age2 = 1.00]	[HIBP = 1.00] * [DPaverage = 1.00]
Constant	.311	.006	.007	.007
[Recur1yr123 = 1.00]	-.311	.023	.007	.005
[Recur1yr123 = 2.00]	-1.082	-.035	-.021	-.011
[HIBP = 1.00]	.728	-.157	-.105	-.239
[DM = 1.00]	-2.501	-.044	.013	.061
[Age2 = 1.00]	.484	.063	-.074	.116
[DPaverage = 1.00]	.308	.021	.013	-.010
[Recur1yr123 = 1.00] * [HIBP = 1.00]	-.798	-.130	-.017	.124
[Recur1yr123 = 2.00] * [HIBP = 1.00]	-.967	-.157	-.148	.095
[DM = 1.00] * [Age2 = 1.00]	-.239	-.181	-.067	.038
[DM = 1.00] * [DPaverage = 1.00]	.143	-.062	.024	-.062
[Recur1yr123 = 1.00] * [DM = 1.00]	2.502	-.018	.015	-.026
[Recur1yr123 = 2.00] * [DM = 1.00]	3.778	.085	.040	-.032
[HIBP = 1.00] * [DM = 1.00]	.085	.393	-.006	-.036
[HIBP = 1.00] * [Age2 = 1.00]	.040	-.006	.285	-.034
[HIBP = 1.00] * [DPaverage = 1.00]	-.032	-.036	-.034	.274
[Age2 = 1.00] * [DPaverage = 1.00]	.009	.031	-.035	-.118

[Recur1yr123 = 1.00] * [Age2 = 1.00]	-.321	.069	-.027	-.050
[Recur1yr123 = 2.00] * [Age2 = 1.00]	-.492	.061	.086	-.056
[Recur1yr123 = 1.00] * [DPaverage = 1.00]	-.408	.033	.013	-.043
[Recur1yr123 = 2.00] * [DPaverage = 1.00]	-.521	.025	.022	.019

Covariances of Parameter Estimates[a,b,c]

	[Age2 = 1.00] * [DPaverage = 1.00]	[Recur1yr123 = 1.00] * [Age2 = 1.00]	[Recur1yr123 = 2.00] * [Age2 = 1.00]
Constant	.015	.163	.153
[Recur1yr123 = 1.00]	.001	-.177	-.140
[Recur1yr123 = 2.00]	.044	-.189	-3502643.029
[HIBP = 1.00]	.120	1.488	1.441
[DM = 1.00]	.035	.421	.413
[Age2 = 1.00]	-.238	-2.977	-3.045
[DPaverage = 1.00]	-.022	.363	.386
[Recur1yr123 = 1.00] * [HIBP = 1.00]	-.051	-1.528	-1.498
[Recur1yr123 = 2.00] * [HIBP = 1.00]	-.022	-1.483	-1.747
[DM = 1.00] * [Age2 = 1.00]	-.044	-.137	-.126
[DM = 1.00] * [DPaverage = 1.00]	-.029	.006	.013
[Recur1yr123 = 1.00] * [DM = 1.00]	-.012	-.400	-.385
[Recur1yr123 = 2.00] * [DM = 1.00]	.009	-.321	-.492
[HIBP = 1.00] * [DM = 1.00]	.031	.069	.061
[HIBP = 1.00] * [Age2 = 1.00]	-.035	-.027	.086
[HIBP = 1.00] * [DPaverage = 1.00]	-.118	-.050	-.056
[Age2 = 1.00] * [DPaverage = 1.00]	.278	.125	.131
[Recur1yr123 = 1.00] * [Age2 = 1.00]	.125	3.093	3.014
[Recur1yr123 = 2.00] * [Age2 = 1.00]	.131	3.014	3502646.442

[Recur1yr123 = 1.00] * [DPaverage = 1.00]	-.046	-.422	-.438
[Recur1yr123 = 2.00] * [DPaverage = 1.00]	-.161	-.463	-.640

Covariances of Parameter Estimates[a,b,c]

	[Recur1yr123 = 1.00] * [DPaverage = 1.00]	[Recur1yr123 = 2.00] * [DPaverage = 1.00]
Constant	3431360.538	3431360.530
[Recur1yr123 = 1.00]	-3431360.598	-3431360.587
[Recur1yr123 = 2.00]	-3431360.591	-3431360.829
[HIBP = 1.00]	.391	.328
[DM = 1.00]	.460	.464
[Age2 = 1.00]	.406	.506
[DPaverage = 1.00]	-3431361.572	-3431361.561
[Recur1yr123 = 1.00] * [HIBP = 1.00]	-.412	-.375
[Recur1yr123 = 2.00] * [HIBP = 1.00]	-.400	-.492
[DM = 1.00] * [Age2 = 1.00]	.031	.052
[DM = 1.00] * [DPaverage = 1.00]	-.153	-.163
[Recur1yr123 = 1.00] * [DM = 1.00]	-.430	-.427
[Recur1yr123 = 2.00] * [DM = 1.00]	-.408	-.521
[HIBP = 1.00] * [DM = 1.00]	.033	.025
[HIBP = 1.00] * [Age2 = 1.00]	.013	.022
[HIBP = 1.00] * [DPaverage = 1.00]	-.043	.019
[Age2 = 1.00] * [DPaverage = 1.00]	-.046	-.161
[Recur1yr123 = 1.00] * [Age2 = 1.00]	-.422	-.463
[Recur1yr123 = 2.00] * [Age2 = 1.00]	-.438	-.640
[Recur1yr123 = 1.00] * [DPaverage = 1.00]	3431361.802	3431361.775
[Recur1yr123 = 2.00] * [DPaverage = 1.00]	3431361.775	3431362.499

a. Model: Poisson

b. Design: Constant + Recur1yr123 + HIBP + DM + Age2 + DPaverage + Recur1yr123 * HIBP + DM * Age2 + DM * DPaverage + Recur1yr123 * DM + HIBP * DM + HIBP * Age2 + HIBP * DPaverage + Age2 * DPaverage + Recur1yr123 * Age2 + Recur1yr123 * DPaverage

c. Redundant parameters are not displayed.

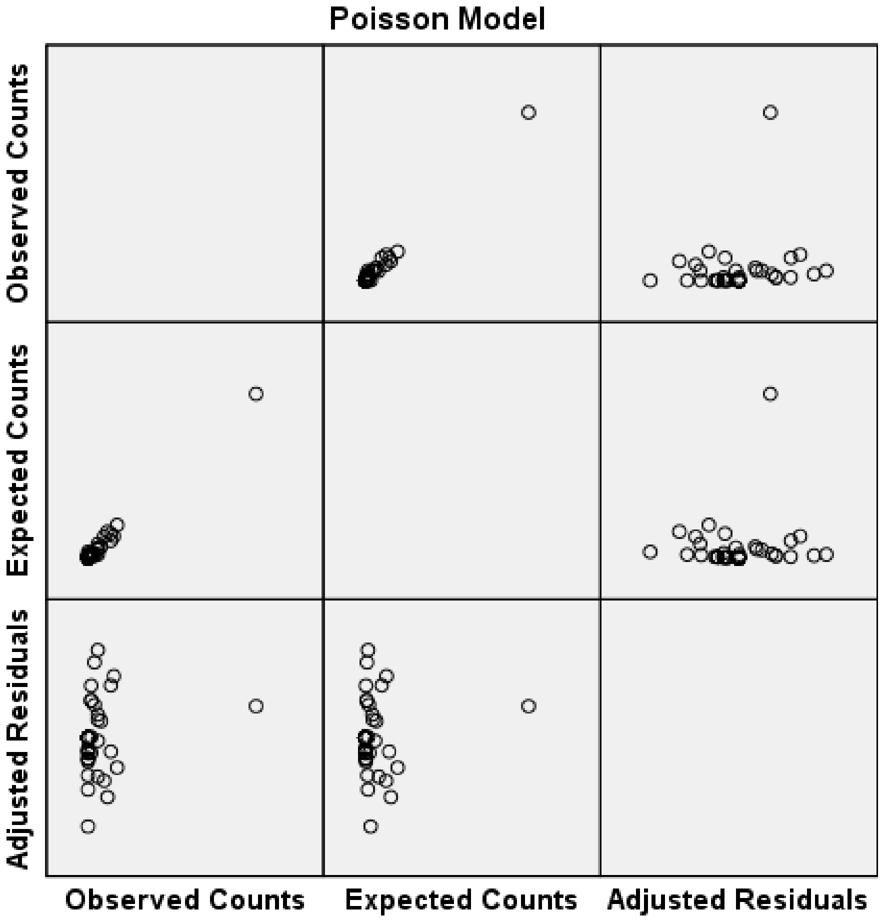

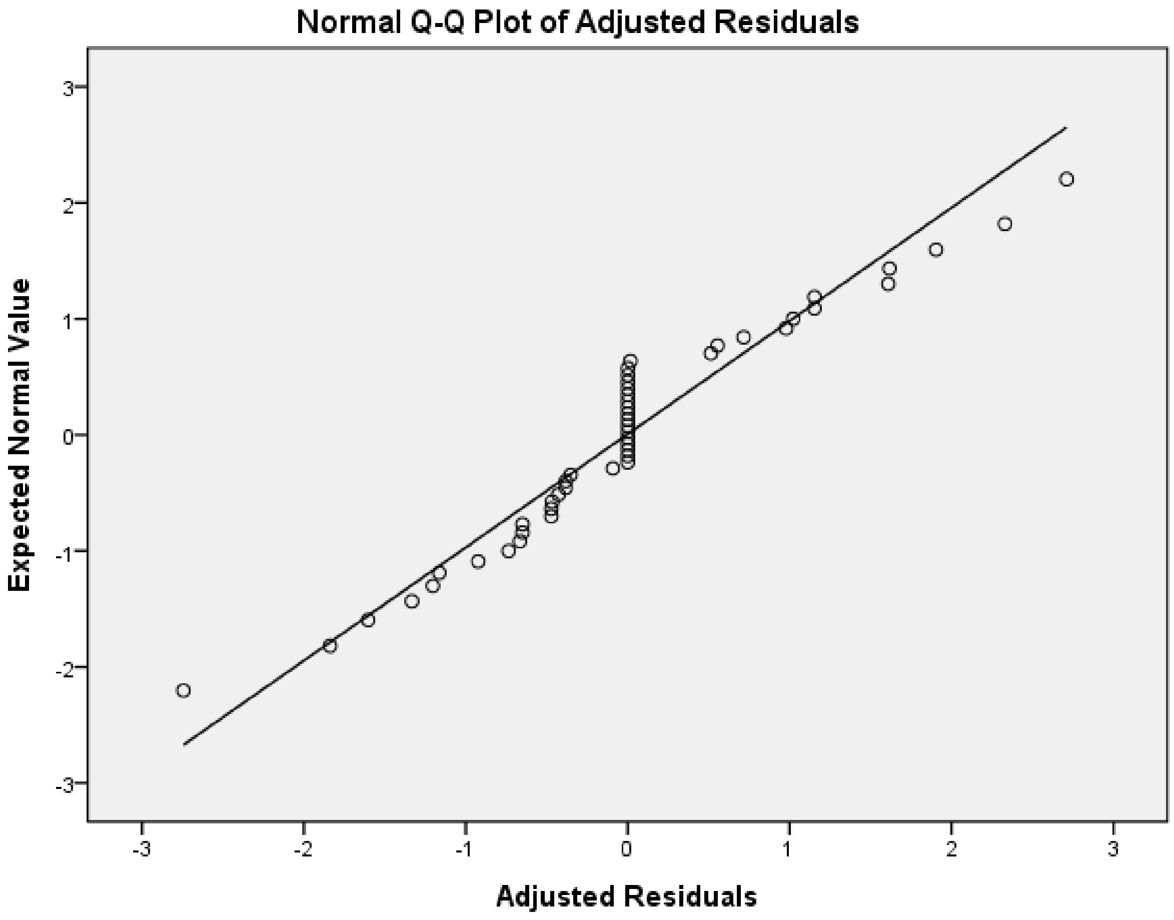
Normal Q-Q Plot of Adjusted Residuals
Expected Normal Value
Adjusted Residuals
3
2
1
0
-1
-2
-3

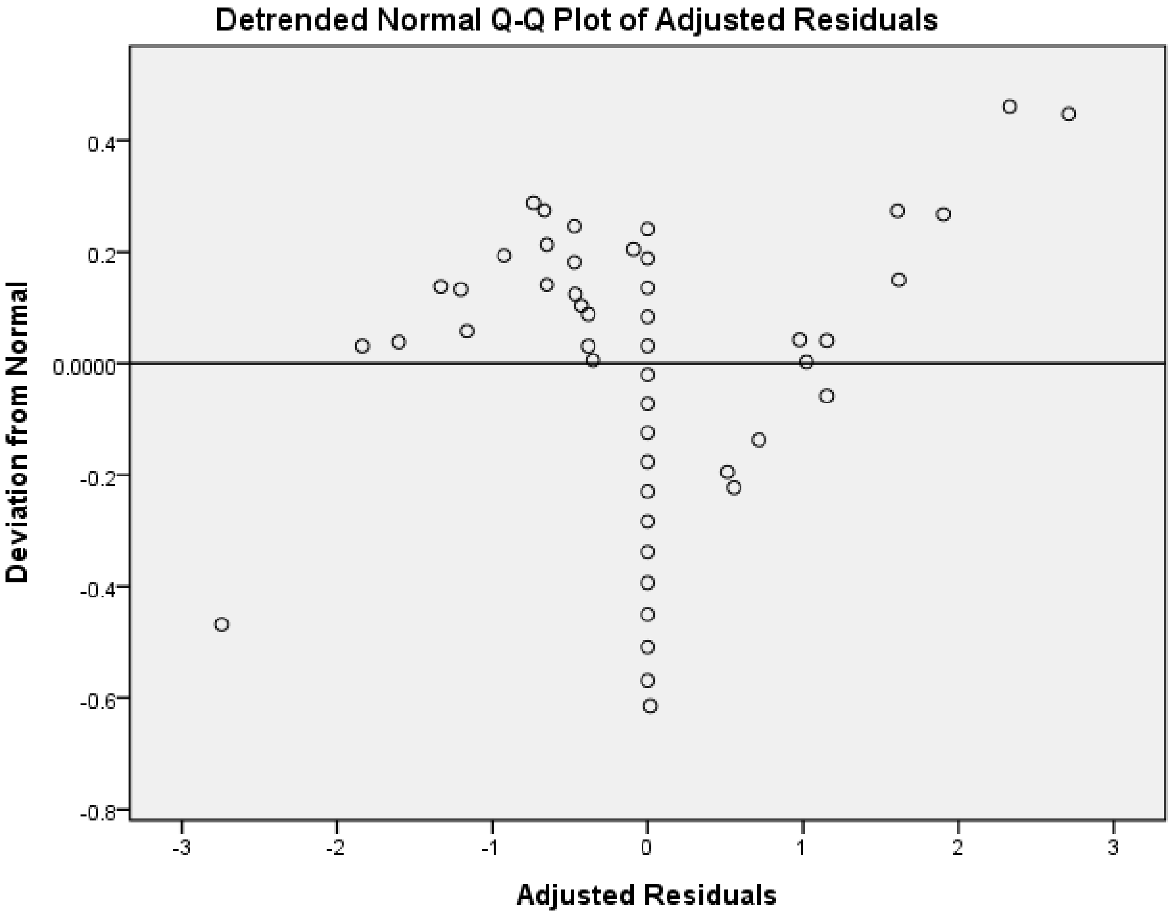


```
USE ALL.
COMPUTE filter_$=(sex = 2).
VARIABLE LABELS filter_$ 'sex = 2 (FILTER)'.
VALUE LABELS filter_$ 0 'Not Selected' 1 'Selected'.
FORMATS filter_$ (f1.0).
FILTER BY filter_$.
EXECUTE.
USE ALL.
COMPUTE filter_$=(sex = 1).
VARIABLE LABELS filter_$ 'sex = 1 (FILTER)'.
VALUE LABELS filter_$ 0 'Not Selected' 1 'Selected'.
FORMATS filter_$ (f1.0).
FILTER BY filter_$.
EXECUTE.
GENLOG Recur1yr123 BY HIBP DM Age2 DPaverage
  /MODEL=MULTINOMIAL
  /PRINT=FREQ RESID ADJRESID ZRESID DEV ESTIM CORR COV
  /PLOT=RESID(ADJRESID) NORMPROB(ADJRESID)
  /CRITERIA=CIN(95) ITERATE(20) CONVERGE(0.001) DELTA(.5)
  /DESIGN Recur1yr123 Recur1yr123*HIBP Recur1yr123*DM Recur1yr123*Age2
Recur1yr123*DPaverage.
```

logit Loglinear male

Notes

Output Created		14:4월:202319시 48분 12초
Comments		
Input	Data	C:\Users\guzi9\OneDrive\바탕 화면\자율\result\ex3.sav
	Active Dataset	DataSet1
	Filter	sex = 1 (FILTER)
	Weight	<none>
	Split File	<none>
	N of Rows in Working Data File	122
Missing Value Handling	Definition of Missing	User-defined missing values are treated as missing.
	Cases Used	Statistics are based on all cases with valid data for all variables in the model.
Syntax		GENLOG Recur1yr123 BY HIBP DM Age2 DPaverage<br>/MODEL=MULTINOMIAL<br>/PRINT=FREQ RESID ADJRESID ZRESID DEV ESTIM CORR COV<br>/PLOT=RESID(ADJRESID) NORMPROB(ADJRESID)<br>/CRITERIA=CIN(95) ITERATE(20) CONVERGE(0.001) DELTA(.5)<br>/DESIGN Recur1yr123 Recur1yr123*HIBP Recur1yr123*DM Recur1yr123*Age2 Recur1yr123*DPaverage.
Resources	Processor Time	00 00:00:00.640
	Elapsed Time	00 00:00:00.610

```
[DataSet1] C:\Users\guzi9\OneDrive\바탕 화면\자율\result\ex3.sav
```

Data Information

		N
Cases	Valid	122
	Missing	0
	Weighted Valid	122
Cells	Defined Cells	48
	Structural Zeros	0
	Sampling Zeros	28
Categories	Recur1yr123	3
	HIBP	2
	DM	2
	Age2	2
	DPaverage	2

Convergence Information[b,c]

Maximum Number of Iterations	20
Converge Tolerance	.00100
Final Maximum Absolute Difference	7.09151E-8[a]
Final Maximum Relative Difference	7.46213E-6
Number of Iterations	20

a. The iteration converged because the maximum absolute changes of parameter estimates is less than the specified convergence criterion.

b. Model: Multinomial Logit

c. Design: Constant + Recur1yr123 + Recur1yr123 * HIBP + Recur1yr123 * DM + Recur1yr123 * Age2 + Recur1yr123 * DPaverage

Goodness-of-Fit Tests[a,b]

	Value	df	Sig.
Likelihood Ratio	8.290	22	.996
Pearson Chi-Square	5.813	22	1.000

Goodness-of-Fit Tests[a,b]

	Value	df	Sig.
Likelihood Ratio	8.290	22	.996
Pearson Chi-Square	5.813	22	1.000

a. Model: Multinomial Logit

b. Design: Constant + Recur1yr123 + Recur1yr123 * HIBP + Recur1yr123 * DM + Recur1yr123 * Age2 + Recur1yr123 * DPaverage

Analysis of Dispersion[a,b]

	Entropy	Concentration	df
Model	7.162	1.586	8
Residual	39.809	22.005	234
Total	46.971	23.590	242

a. Model: Multinomial Logit

b. Design: Constant + Recur1yr123 + Recur1yr123 * HIBP + Recur1yr123 * DM + Recur1yr123 * Age2 + Recur1yr123 * DPaverage

Measure of Association[a,b]

Entropy	.152
Concentration	.067

a. Model: Multinomial Logit

b. Design: Constant + Recur1yr123 + Recur1yr123 * HIBP + Recur1yr123 * DM + Recur1yr123 * Age2 + Recur1yr123 * DPaverage

Cell Counts and Residuals[a,b]

					Observed		Expected	
HIBP	DM	Age2	DPaverage	Recur1yr123	Count	%	Count	%
1.00	1.00	1.00	1.00	1.00	52	85.2%	53.150	87.1%
				2.00	8	13.1%	7.208	11.8%

				3.00	1	1.6%	.643	1.1%
			2.00	1.00	9	100.0%	8.444	93.8%
				2.00	0	.0%	.556	6.2%
				3.00	0	.0%	.000	.0%
		2.00	1.00	1.00	5	100.0%	4.860	97.2%
				2.00	0	.0%	.000	.0%
				3.00	0	.0%	.140	2.8%
			2.00	1.00	4	100.0%	4.000	100.0%
				2.00	0	.0%	.000	.0%
				3.00	0	.0%	.000	.0%
	2.00	1.00	1.00	1.00	3	100.0%	2.582	86.1%
				2.00	0	.0%	.200	6.7%
				3.00	0	.0%	.217	7.2%
			2.00	1.00	1	100.0%	.964	96.4%
				2.00	0	.0%	.036	3.6%
				3.00	0	.0%	.000	.0%
		2.00	1.00	1.00	0	.0%	.000	.0%
				2.00	0	.0%	.000	.0%
				3.00	0	.0%	.000	.0%
			2.00	1.00	3	100.0%	3.000	100.0%
				2.00	0	.0%	.000	.0%
				3.00	0	.0%	.000	.0%
2.00	1.00	1.00	1.00	1.00	3	100.0%	1.864	62.1%
				2.00	0	.0%	1.113	37.1%
				3.00	0	.0%	.022	.7%
			2.00	1.00	3	60.0%	3.877	77.5%
				2.00	2	40.0%	1.123	22.5%
				3.00	0	.0%	.000	.0%
		2.00	1.00	1.00	7	100.0%	6.805	97.2%
				2.00	0	.0%	.000	.0%
				3.00	0	.0%	.195	2.8%
			2.00	1.00	7	100.0%	7.000	100.0%
				2.00	0	.0%	.000	.0%
				3.00	0	.0%	.000	.0%
	2.00	1.00	1.00	1.00	1	50.0%	1.403	70.2%
				2.00	1	50.0%	.479	24.0%
				3.00	0	.0%	.118	5.9%
			2.00	1.00	2	100.0%	1.716	85.8%

			2.00	0	.0%	.284	14.2%
			3.00	0	.0%	.000	.0%
	2.00	1.00	1.00	3	75.0%	3.335	83.4%
			2.00	0	.0%	.000	.0%
			3.00	1	25.0%	.665	16.6%
		2.00	1.00	6	100.0%	6.000	100.0%
			2.00	0	.0%	.000	.0%
			3.00	0	.0%	.000	.0%

Cell Counts and Residuals[a,b]

HIBP	DM	Age2	DPaverage	Recur1yr123	Residual	Standardized Residual	Adjusted Residual	Deviance
1.00	1.00	1.00	1.00	1.00	-1.150	-.440	-1.694	-1.508
				2.00	.792	.314	1.330	1.292
				3.00	.357	.448	1.041	.940
			2.00	1.00	.556	.770	1.081	1.071
				2.00	-.556	-.770	-1.081	.000
				3.00	.000	.000	.000	.000
		2.00	1.00	1.00	.140	.379	.587	.533
				2.00	.000	.000	.000	.000
				3.00	-.140	-.379	-.587	.000
			2.00	1.00	.000	.000	.000	.000
				2.00	.000	.000	.000	.000
				3.00	.000	.000	.000	.000
	2.00	1.00	1.00	1.00	.418	.697	.932	.948
				2.00	-.200	-.463	-.552	.000
				3.00	-.217	-.484	-.770	.000
			2.00	1.00	.036	.194	.203	.272
				2.00	-.036	-.194	-.203	.000
				3.00	.000	.000	.000	.000
		2.00	1.00	1.00	.000	.000	.	.000
				2.00	.000	.000	.	.000
				3.00	.000	.000	.	.000
			2.00	1.00	.000	.000	.000	.000
				2.00	.000	.000	.000	.000
				3.00	.000	.000	.000	.000
2.00	1.00	1.00	1.00	1.00	1.136	1.352	2.087	1.689
				2.00	-1.113	-1.330	-2.086	.000
				3.00	-.022	-.150	-.163	.000

		2.00	1.00	-.877	-.939	-1.671	-1.240
			2.00	.877	.939	1.671	1.519
			3.00	.000	.000	.000	.000
	2.00	1.00	1.00	.195	.448	.675	.629
			2.00	.000	.000	.000	.000
			3.00	-.195	-.448	-.675	.000
		2.00	1.00	.000	.000	.000	.000
			2.00	.000	.000	.000	.000
			3.00	.000	.000	.000	.000
2.00	1.00	1.00	1.00	-.403	-.623	-.875	-.823
			2.00	.521	.863	1.260	1.213
			3.00	-.118	-.353	-.489	.000
		2.00	1.00	.284	.576	.728	.783
			2.00	-.284	-.576	-.728	.000
			3.00	.000	.000	.000	.000
	2.00	1.00	1.00	-.335	-.450	-1.042	-.797
			2.00	.000	.000	.000	.000
			3.00	.335	.450	1.042	.903
		2.00	1.00	.000	.000	.000	.000
			2.00	.000	.000	.000	.000
			3.00	.000	.000	.000	.000

a. Model: Multinomial Logit

b. Design: Constant + Recur1yr123 + Recur1yr123 * HIBP + Recur1yr123 * DM + Recur1yr123 * Age2 + Recur1yr123 * DPaverage

Parameter Estimates[c,d,e]

Parameter		Estimate	Std. Error	Z	Sig.	95% Confidence Interval Lower Bound	Upper Bound
Constant	[HIBP = 1.00] * [DM = 1.00] * [Age2 = 1.00] * [DPaverage = 1.00]	-.442[a]					
	[HIBP = 1.00] * [DM = 1.00] * [Age2 = 1.00] * [DPaverage = 2.00]	-20.392[a]					

	[HIBP = 1.00] * [DM = 1.00] * [Age2 = 2.00] * [DPaverage = 1.00]	-1.966[a]					
	[HIBP = 1.00] * [DM = 1.00] * [Age2 = 2.00] * [DPaverage = 2.00]	-20.272[a]					
	[HIBP = 1.00] * [DM = 2.00] * [Age2 = 1.00] * [DPaverage = 1.00]	-1.526[a]					
	[HIBP = 1.00] * [DM = 2.00] * [Age2 = 1.00] * [DPaverage = 2.00]	-20.622[a]					
	[HIBP = 1.00] * [DM = 2.00] * [Age2 = 2.00] * [DPaverage = 1.00]	.000[a]					
	[HIBP = 1.00] * [DM = 2.00] * [Age2 = 2.00] * [DPaverage = 2.00]	-18.619[a]					
	[HIBP = 2.00] * [DM = 1.00] * [Age2 = 1.00] * [DPaverage = 1.00]	-3.797[a]					
	[HIBP = 2.00] * [DM = 1.00] * [Age2 = 1.00] * [DPaverage = 2.00]	-21.176[a]					
	[HIBP = 2.00] * [DM = 1.00] * [Age2 = 2.00] * [DPaverage = 1.00]	-1.635[a]					
	[HIBP = 2.00] * [DM = 1.00] * [Age2 = 2.00] * [DPaverage = 2.00]	-19.717[a]					
	[HIBP = 2.00] * [DM = 2.00] * [Age2 = 1.00] * [DPaverage = 1.00]	-2.141[a]					
	[HIBP = 2.00] * [DM = 2.00] * [Age2 = 1.00] * [DPaverage = 2.00]	-20.051[a]					
	[HIBP = 2.00] * [DM = 2.00] * [Age2 = 2.00] * [DPaverage = 1.00]	-.408[a]					

[HIBP = 2.00] * [DM = 2.00] * [Age2 = 2.00] * [DPaverage = 2.00]	-17.931[a]					
[Recur1yr123 = 1.00]	19.723	1.551	12.715	.000	16.683	22.763
[Recur1yr123 = 2.00]	-.680	2.322	-.293	.770	-5.231	3.872
[Recur1yr123 = 3.00]	0[b]	.	.	.	.	.
[Recur1yr123 = 1.00] * [HIBP = 1.00]	-.005	2.407	-.002	.998	-4.722	4.712
[Recur1yr123 = 1.00] * [HIBP = 2.00]	0[b]	.	.	.	.	.
[Recur1yr123 = 2.00] * [HIBP = 1.00]	-1.487	2.549	-.584	.560	-6.483	3.508
[Recur1yr123 = 2.00] * [HIBP = 2.00]	0[b]	.	.	.	.	.
[Recur1yr123 = 3.00] * [HIBP = 1.00]	0[b]	.	.	.	.	.
[Recur1yr123 = 3.00] * [HIBP = 2.00]	0[b]	.	.	.	.	.
[Recur1yr123 = 1.00] * [DM = 1.00]	1.940	1.785	1.087	.277	-1.558	5.439
[Recur1yr123 = 1.00] * [DM = 2.00]	0[b]	.	.	.	.	.
[Recur1yr123 = 2.00] * [DM = 1.00]	2.499	2.139	1.169	.243	-1.693	6.691
[Recur1yr123 = 2.00] * [DM = 2.00]	0[b]	.	.	.	.	.
[Recur1yr123 = 3.00] * [DM = 1.00]	0[b]	.	.	.	.	.
[Recur1yr123 = 3.00] * [DM = 2.00]	0[b]	.	.	.	.	.
[Recur1yr123 = 1.00] * [Age2 = 1.00]	.868	2.112	.411	.681	-3.271	5.007
[Recur1yr123 = 1.00] * [Age2 = 2.00]	0[b]	.	.	.	.	.
[Recur1yr123 = 2.00] * [Age2 = 1.00]	.000	.	.	.	.	.
[Recur1yr123 = 2.00] * [Age2 = 2.00]	0[b]	.	.	.	.	.
[Recur1yr123 = 3.00] * [Age2 = 1.00]	0[b]	.	.	.	.	.
[Recur1yr123 = 3.00] * [Age2 = 2.00]	0[b]	.	.	.	.	.
[Recur1yr123 = 1.00] * [DPaverage = 1.00]	-18.111	.975	-18.577	.000	-20.021	-16.200
[Recur1yr123 = 1.00] * [DPaverage = 2.00]	0[b]	.	.	.	.	.
[Recur1yr123 = 2.00] * [DPaverage = 1.00]	.000	.	.	.	.	.

[Recur1yr123 = 2.00] * [DPaverage = 2.00]	0[b]	.	.	.	.	.
[Recur1yr123 = 3.00] * [DPaverage = 1.00]	0[b]	.	.	.	.	.
[Recur1yr123 = 3.00] * [DPaverage = 2.00]	0[b]	.	.	.	.	.

a. Constants are not parameters under the multinomial assumption. Therefore, their standard errors are not calculated.

b. This parameter is set to zero because it is redundant.

c. Model: Multinomial Logit

d. Design: Constant + Recur1yr123 + Recur1yr123 * HIBP + Recur1yr123 * DM + Recur1yr123 * Age2 + Recur1yr123 * DPaverage

e. Some of the parameter estimates are estimated to be zeros because the Hessian matrix is singular and cannot be inverted. Therefore a generalized inverse of the Hessian matrix is computed instead.

Correlations of Parameter Estimates[a,b,c]

	[Recur1yr123 = 1.00]	[Recur1yr123 = 2.00]	[Recur1yr123 = 1.00] * [HIBP = 1.00]	[Recur1yr123 = 2.00] * [HIBP = 1.00]
[Recur1yr123 = 1.00]	1	.278	-.011	-.118
[Recur1yr123 = 2.00]	.278	1	-.614	-.634
[Recur1yr123 = 1.00] * [HIBP = 1.00]	-.011	-.614	1	.929
[Recur1yr123 = 2.00] * [HIBP = 1.00]	-.118	-.634	.929	1
[Recur1yr123 = 1.00] * [DM = 1.00]	-.311	-.069	-.495	-.461
[Recur1yr123 = 2.00] * [DM = 1.00]	-.228	-.248	-.421	-.467
[Recur1yr123 = 1.00] * [Age2 = 1.00]	-.263	.713	-.679	-.630
[Recur1yr123 = 2.00] * [Age2 = 1.00]	.	.	.	.
[Recur1yr123 = 1.00] * [DPaverage = 1.00]	-.625	-.161	-.028	.146
[Recur1yr123 = 2.00] * [DPaverage = 1.00]	.	.	.	.

Correlations of Parameter Estimates[a,b,c]

	[Recur1yr123 = 1.00] * [DM = 1.00]	[Recur1yr123 = 2.00] * [DM = 1.00]	[Recur1yr123 = 1.00] * [Age2 = 1.00]	[Recur1yr123 = 2.00] * [Age2 = 1.00]
[Recur1yr123 = 1.00]	-.311	-.228	-.263	.
[Recur1yr123 = 2.00]	-.069	-.248	.713	.
[Recur1yr123 = 1.00] * [HIBP = 1.00]	-.495	-.421	-.679	.
[Recur1yr123 = 2.00] * [HIBP = 1.00]	-.461	-.467	-.630	.
[Recur1yr123 = 1.00] * [DM = 1.00]	1	.815	.139	.
[Recur1yr123 = 2.00] * [DM = 1.00]	.815	1	.137	.
[Recur1yr123 = 1.00] * [Age2 = 1.00]	.139	.137	1	.
[Recur1yr123 = 2.00] * [Age2 = 1.00]	.	.	.	.
[Recur1yr123 = 1.00] * [DPaverage = 1.00]	.007	-.028	.032	.
[Recur1yr123 = 2.00] * [DPaverage = 1.00]	.	.	.	.

Correlations of Parameter Estimates[a,b,c]

	[Recur1yr123 = 1.00] * [DPaverage = 1.00]	[Recur1yr123 = 2.00] * [DPaverage = 1.00]
[Recur1yr123 = 1.00]	-.625	.
[Recur1yr123 = 2.00]	-.161	.
[Recur1yr123 = 1.00] * [HIBP = 1.00]	-.028	.
[Recur1yr123 = 2.00] * [HIBP = 1.00]	.146	.
[Recur1yr123 = 1.00] * [DM = 1.00]	.007	.
[Recur1yr123 = 2.00] * [DM = 1.00]	-.028	.
[Recur1yr123 = 1.00] * [Age2 = 1.00]	.032	.
[Recur1yr123 = 2.00] * [Age2 = 1.00]	.	.
[Recur1yr123 = 1.00] * [DPaverage = 1.00]	1	.

Correlations of Parameter Estimates[a,b,c]

	[Recur1yr123 = 1.00] * [DPaverage = 1.00]	[Recur1yr123 = 2.00] * [DPaverage = 1.00]
[Recur1yr123 = 1.00]	-.625	.
[Recur1yr123 = 2.00]	-.161	.
[Recur1yr123 = 1.00] * [HIBP = 1.00]	-.028	.
[Recur1yr123 = 2.00] * [HIBP = 1.00]	.146	.
[Recur1yr123 = 1.00] * [DM = 1.00]	.007	.
[Recur1yr123 = 2.00] * [DM = 1.00]	-.028	.
[Recur1yr123 = 1.00] * [Age2 = 1.00]	.032	.
[Recur1yr123 = 2.00] * [Age2 = 1.00]	.	.
[Recur1yr123 = 1.00] * [DPaverage = 1.00]	1	.
[Recur1yr123 = 2.00] * [DPaverage = 1.00]	.	.

a. Model: Multinomial Logit

b. Design: Constant + Recur1yr123 + Recur1yr123 * HIBP + Recur1yr123 * DM + Recur1yr123 * Age2 + Recur1yr123 * DPaverage

c. Constants and redundant parameters are not displayed.

Covariances of Parameter Estimates[a,b,c]

	[Recur1yr123 = 1.00]	[Recur1yr123 = 2.00]	[Recur1yr123 = 1.00] * [HIBP = 1.00]	[Recur1yr123 = 2.00] * [HIBP = 1.00]
[Recur1yr123 = 1.00]	2.406	1.000	-.042	-.468
[Recur1yr123 = 2.00]	1.000	5.393	-3.432	-3.752
[Recur1yr123 = 1.00] * [HIBP = 1.00]	-.042	-3.432	5.792	5.699
[Recur1yr123 = 2.00] * [HIBP = 1.00]	-.468	-3.752	5.699	6.496

[Recur1yr123 = 1.00] * [DM = 1.00]	-.862	-.284	-2.127	-2.099
[Recur1yr123 = 2.00] * [DM = 1.00]	-.758	-1.232	-2.165	-2.548
[Recur1yr123 = 1.00] * [Age2 = 1.00]	-.863	3.497	-3.453	-3.393
[Recur1yr123 = 2.00] * [Age2 = 1.00]	.000	.000	.000	.000
[Recur1yr123 = 1.00] * [DPaverage = 1.00]	-.945	-.364	-.067	.364
[Recur1yr123 = 2.00] * [DPaverage = 1.00]	.000	.000	.000	.000

Covariances of Parameter Estimates[a,b,c]

	[Recur1yr123 = 1.00] * [DM = 1.00]	[Recur1yr123 = 2.00] * [DM = 1.00]	[Recur1yr123 = 1.00] * [Age2 = 1.00]	[Recur1yr123 = 2.00] * [Age2 = 1.00]
[Recur1yr123 = 1.00]	-.862	-.758	-.863	.000
[Recur1yr123 = 2.00]	-.284	-1.232	3.497	.000
[Recur1yr123 = 1.00] * [HIBP = 1.00]	-2.127	-2.165	-3.453	.000
[Recur1yr123 = 2.00] * [HIBP = 1.00]	-2.099	-2.548	-3.393	.000
[Recur1yr123 = 1.00] * [DM = 1.00]	3.187	3.110	.523	.000
[Recur1yr123 = 2.00] * [DM = 1.00]	3.110	4.575	.619	.000
[Recur1yr123 = 1.00] * [Age2 = 1.00]	.523	.619	4.460	.000
[Recur1yr123 = 2.00] * [Age2 = 1.00]	.000	.000	.000	.000
[Recur1yr123 = 1.00] * [DPaverage = 1.00]	.012	-.058	.067	.000
[Recur1yr123 = 2.00] * [DPaverage = 1.00]	.000	.000	.000	.000

Covariances of Parameter Estimates[a,b,c]

	[Recur1yr123 = 1.00] * [DPaverage = 1.00]	[Recur1yr123 = 2.00] * [DPaverage = 1.00]
[Recur1yr123 = 1.00]	-.945	.000
[Recur1yr123 = 2.00]	-.364	.000

[Recur1yr123 = 1.00] * [HIBP = 1.00]	-.067	.000
[Recur1yr123 = 2.00] * [HIBP = 1.00]	.364	.000
[Recur1yr123 = 1.00] * [DM = 1.00]	.012	.000
[Recur1yr123 = 2.00] * [DM = 1.00]	-.058	.000
[Recur1yr123 = 1.00] * [Age2 = 1.00]	.067	.000
[Recur1yr123 = 2.00] * [Age2 = 1.00]	.000	.000
[Recur1yr123 = 1.00] * [DPaverage = 1.00]	.950	.000
[Recur1yr123 = 2.00] * [DPaverage = 1.00]	.000	.000

a. Model: Multinomial Logit

b. Design: Constant + Recur1yr123 + Recur1yr123 * HIBP + Recur1yr123 * DM + Recur1yr123 * Age2 + Recur1yr123 * DPaverage

c. Constants and redundant parameters are not displayed.

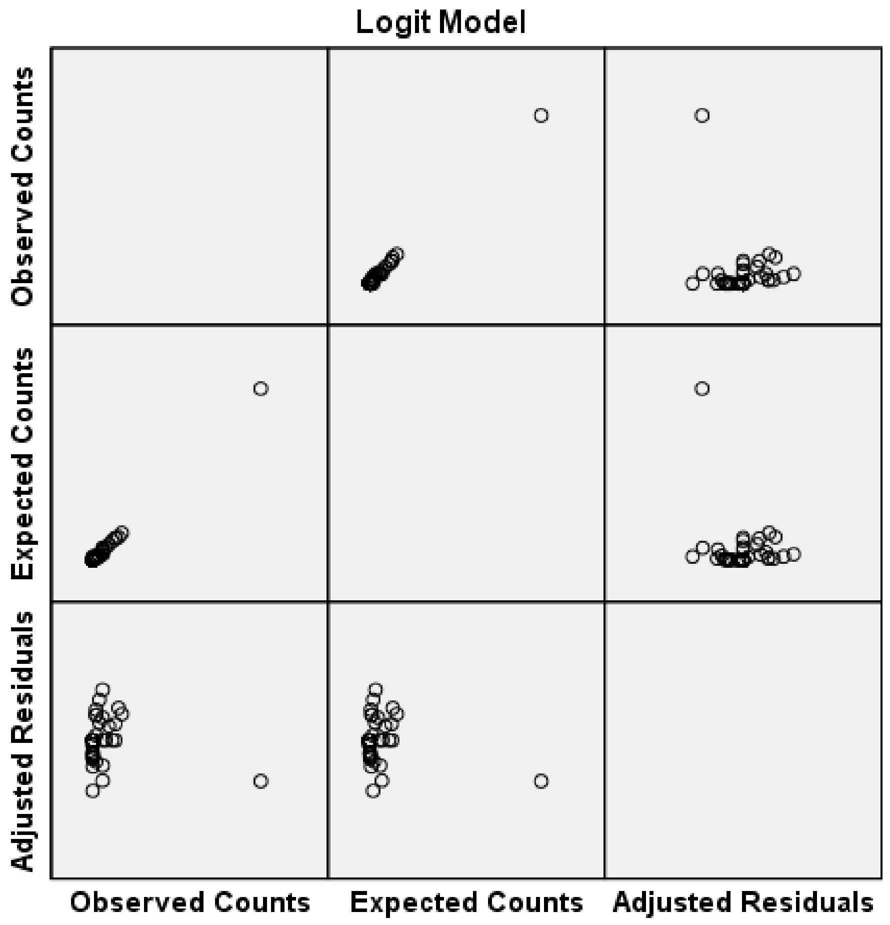
Logit Model
Observed Counts
Expected Counts
Adjusted Residuals
Observed Counts
Expected Counts
Adjusted Residuals

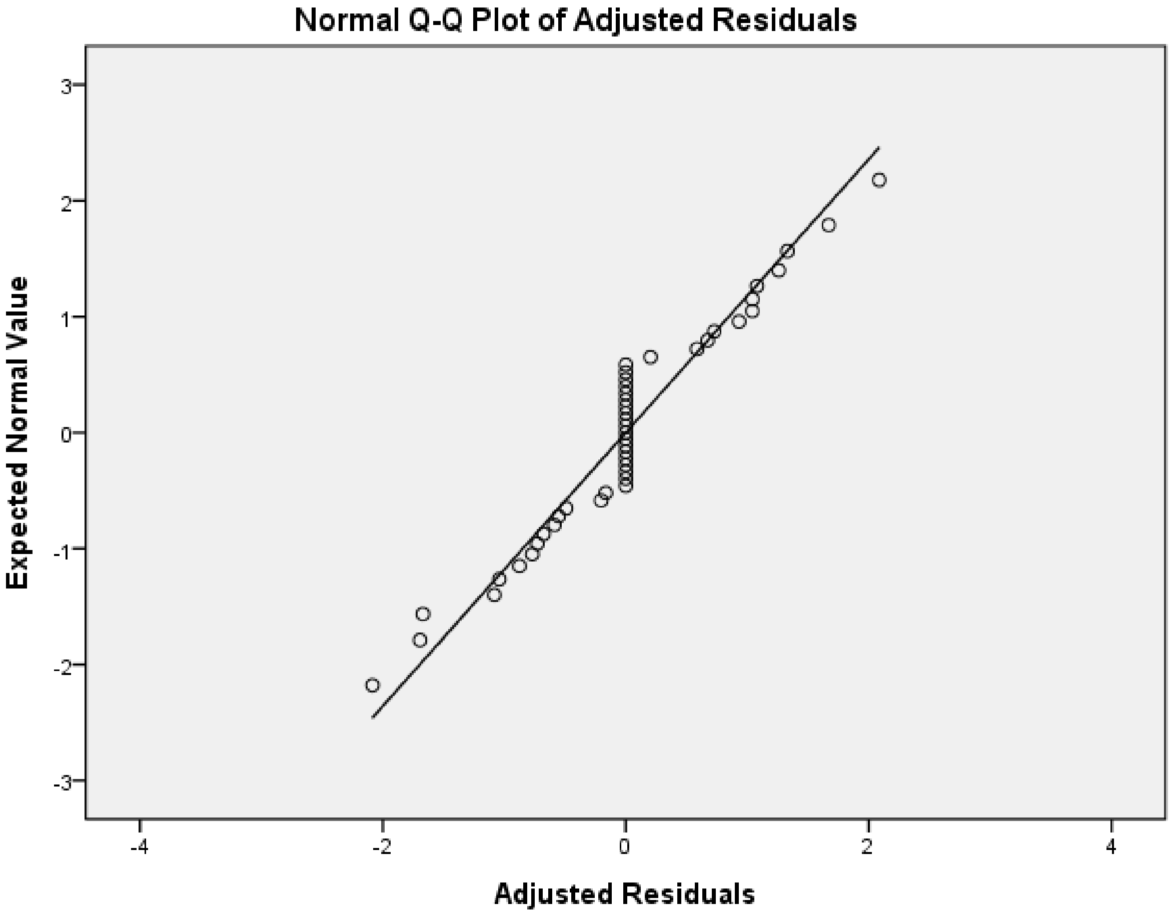

Normal Q-Q Plot of Adjusted Residuals
Expected Normal Value
Adjusted Residuals
3
2
1
0
-1
-2
-3
-4
-2
0
2
4

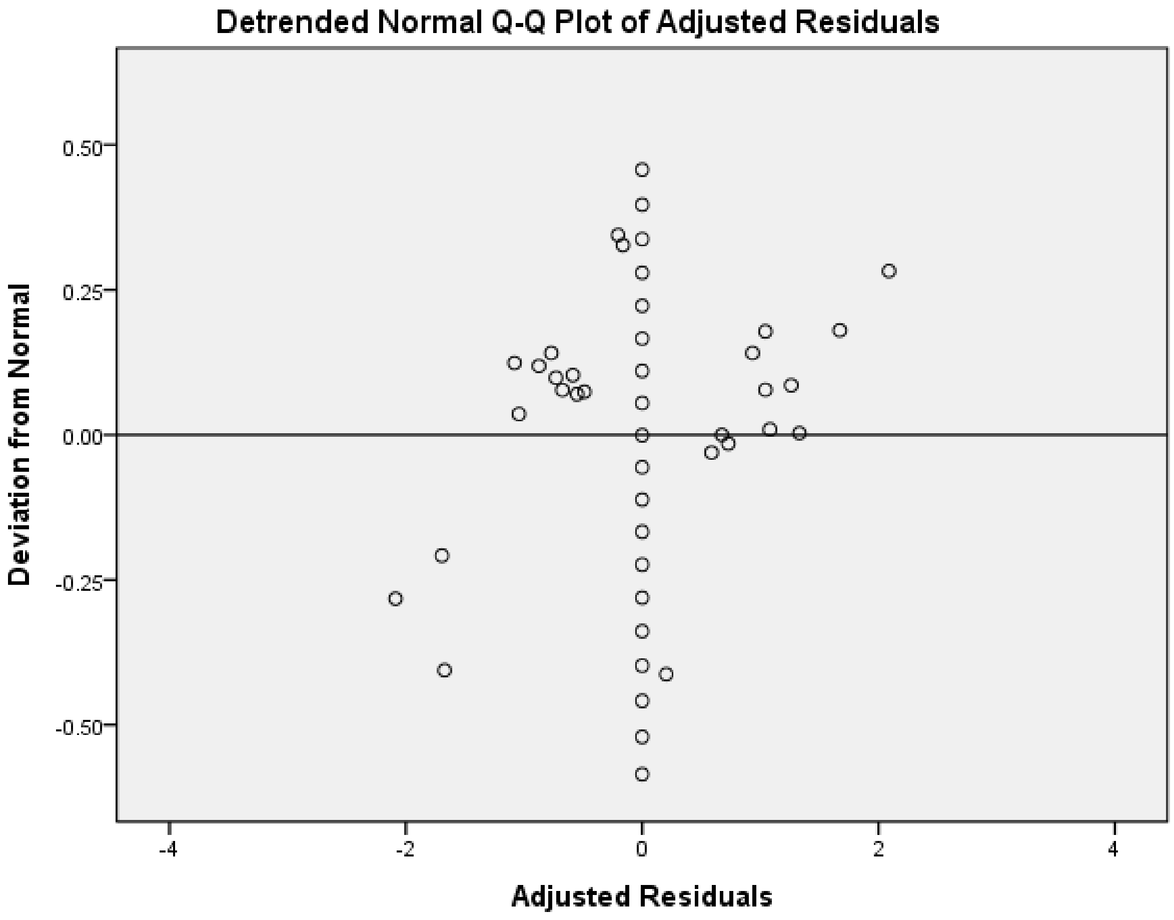


```
USE ALL.
COMPUTE filter_$=(sex = 2).
VARIABLE LABELS filter_$ 'sex = 2 (FILTER)'.
VALUE LABELS filter_$ 0 'Not Selected' 1 'Selected'.
FORMATS filter_$ (f1.0).
FILTER BY filter_$.
EXECUTE.
GENLOG Recur1yr123 HIBP DM Age2 DPaverage
  /MODEL=POISSON
  /PRINT=FREQ RESID ADJRESID ZRESID DEV ESTIM CORR COV
  /PLOT=RESID(ADJRESID) NORMPROB(ADJRESID)
  /CRITERIA=CIN(95) ITERATE(20) CONVERGE(0.001) DELTA(.5)
  /DESIGN Recur1yr123 HIBP DM Age2 DPaverage HIBP*Recur1yr123 Age2*DM
DM*DPaverage DM*Recur1yr123 DM*HIBP Age2*HIBP DPaverage*HIBP Age2*DPaverage
Age2*Recur1yr123 DPaverage*Recur1yr123.
```

General Loglinear female

Notes

Output Created		14:4월:202319시 57분 25초
Comments		
Input	Data	C:\Users\guzi9\OneDrive\바탕 화면\자율\result\ex3.sav
	Active Dataset	DataSet1
	Filter	sex = 2 (FILTER)
	Weight	<none>
	Split File	<none>
	N of Rows in Working Data File	309
Missing Value Handling	Definition of Missing	User-defined missing values are treated as missing.
	Cases Used	Statistics are based on all cases with valid data for all variables in the model.
Syntax		GENLOG Recur1yr123 HIBP DM Age2 DPaverage<br>/MODEL=POISSON<br>/PRINT=FREQ RESID ADJRESID ZRESID DEV ESTIM CORR COV<br>/PLOT=RESID(ADJRESID) NORMPROB(ADJRESID)<br>/CRITERIA=CIN(95) ITERATE(20) CONVERGE(0.001) DELTA(.5)<br>/DESIGN Recur1yr123 HIBP DM Age2 DPaverage HIBP*Recur1yr123 Age2*DM DM*DPaverage DM*Recur1yr123 DM*HIBP Age2*HIBP DPaverage*HIBP Age2*DPaverage Age2*Recur1yr123 DPaverage*Recur1yr123.
Resources	Processor Time	00 00:00:00.625
	Elapsed Time	00 00:00:00.627

```
[DataSet1] C:\Users\guzi9\OneDrive\바탕 화면\자율\result\ex3.sav
```

Data Information

		N
Cases	Valid	309
	Missing	0
	Weighted Valid	309
Cells	Defined Cells	48
	Structural Zeros	0
	Sampling Zeros	16
Categories	Recur1yr123	3
	HIBP	2
	DM	2
	Age2	2
	DPaverage	2

Convergence Information[b,c]

Maximum Number of Iterations	20
Converge Tolerance	.00100
Final Maximum Absolute Difference	1.42840E-6[a]
Final Maximum Relative Difference	2.81080E-6
Number of Iterations	9

a. The iteration converged because the maximum absolute changes of parameter estimates is less than the specified convergence criterion.

b. Model: Poisson

c. Design: Constant + Recur1yr123 + HIBP + DM + Age2 + DPaverage + Recur1yr123 * HIBP + DM * Age2 + DM * DPaverage + Recur1yr123 * DM + HIBP * DM + HIBP * Age2 + HIBP * DPaverage + Age2 * DPaverage + Recur1yr123 * Age2 + Recur1yr123 * DPaverage

Goodness-of-Fit Tests[a,b]

	Value	df	Sig.

Likelihood Ratio	22.406	27	.716
Pearson Chi-Square	22.798	27	.696

a. Model: Poisson

b. Design: Constant + Recur1yr123 + HIBP + DM + Age2 + DPaverage + Recur1yr123 * HIBP + DM * Age2 + DM * DPaverage + Recur1yr123 * DM + HIBP * DM + HIBP * Age2 + HIBP * DPaverage + Age2 * DPaverage + Recur1yr123 * Age2 + Recur1yr123 * DPaverage

Cell Counts and Residuals[a,b]

					Observed		Expected	
Recur1yr123	HIBP	DM	Age2	DPaverage	Count	%	Count	%
1.00	1.00	1.00	1.00	1.00	97	31.4%	98.810	32.0%
				2.00	42	13.6%	40.430	13.1%
			2.00	1.00	24	7.8%	23.514	7.6%
				2.00	9	2.9%	11.573	3.7%
		2.00	1.00	1.00	5	1.6%	5.027	1.6%
				2.00	3	1.0%	2.066	.7%
			2.00	1.00	3	1.0%	1.725	.6%
				2.00	1	.3%	.853	.3%
	2.00	1.00	1.00	1.00	10	3.2%	9.815	3.2%
				2.00	5	1.6%	4.612	1.5%
			2.00	1.00	23	7.4%	21.241	6.9%
				2.00	12	3.9%	12.004	3.9%
		2.00	1.00	1.00	2	.6%	2.880	.9%
				2.00	1	.3%	1.359	.4%
			2.00	1.00	8	2.6%	8.987	2.9%
				2.00	5	1.6%	5.102	1.7%
2.00	1.00	1.00	1.00	1.00	15	4.9%	12.905	4.2%
				2.00	5	1.6%	5.627	1.8%
			2.00	1.00	3	1.0%	4.321	1.4%
				2.00	4	1.3%	2.266	.7%
		2.00	1.00	1.00	0	.0%	.865	.3%
				2.00	0	.0%	.379	.1%
			2.00	1.00	0	.0%	.418	.1%
				2.00	0	.0%	.220	.1%
	2.00	1.00	1.00	1.00	1	.3%	1.549	.5%

				2.00	0	.0%	.776	.3%
			2.00	1.00	4	1.3%	4.716	1.5%
				2.00	3	1.0%	2.841	.9%
		2.00	1.00	1.00	2	.6%	.599	.2%
				2.00	0	.0%	.301	.1%
			2.00	1.00	3	1.0%	2.628	.9%
				2.00	2	.6%	1.590	.5%
3.00	1.00	1.00	1.00	1.00	10	3.2%	10.262	3.3%
				2.00	1	.3%	1.272	.4%
			2.00	1.00	3	1.0%	1.758	.6%
				2.00	0	.0%	.262	.1%
		2.00	1.00	1.00	0	.0%	.317	.1%
				2.00	0	.0%	.039	.0%
			2.00	1.00	0	.0%	.078	.0%
				2.00	0	.0%	.012	.0%
	2.00	1.00	1.00	1.00	1	.3%	.825	.3%
				2.00	0	.0%	.117	.0%
			2.00	1.00	0	.0%	1.284	.4%
				2.00	1	.3%	.220	.1%
		2.00	1.00	1.00	1	.3%	.147	.0%
				2.00	0	.0%	.021	.0%
			2.00	1.00	0	.0%	.330	.1%
				2.00	0	.0%	.057	.0%

Cell Counts and Residuals[a,b]

Recur1yr123	HIBP	DM	Age2	DPaverage	Residual	Standardized Residual	Adjusted Residual	Deviance
1.00	1.00	1.00	1.00	1.00	-1.810	-.182	-.707	-.183
				2.00	1.570	.247	.695	.245
			2.00	1.00	.486	.100	.235	.100
				2.00	-2.573	-.756	-1.415	-.787
		2.00	1.00	1.00	-.027	-.012	-.020	-.012
				2.00	.934	.649	.840	.608
			2.00	1.00	1.275	.970	1.233	.877
				2.00	.147	.159	.184	.155
	2.00	1.00	1.00	1.00	.185	.059	.104	.059
				2.00	.388	.181	.258	.178
			2.00	1.00	1.759	.382	.854	.377
				2.00	-.004	-.001	-.002	-.001

		2.00	1.00	1.00	-.880	-.518	-.731	-.549
				2.00	-.359	-.308	-.376	-.323
			2.00	1.00	-.987	-.329	-.616	-.335
				2.00	-.102	-.045	-.072	-.045
2.00	1.00	1.00	1.00	1.00	2.095	.583	1.210	.569
				2.00	-.627	-.264	-.425	-.270
			2.00	1.00	-1.321	-.635	-.976	-.673
				2.00	1.734	1.152	1.511	1.038
		2.00	1.00	1.00	-.865	-.930	-1.092	-.930
				2.00	-.379	-.616	-.671	-.616
			2.00	1.00	-.418	-.646	-.713	-.646
				2.00	-.220	-.469	-.498	-.469
	2.00	1.00	1.00	1.00	-.549	-.441	-.544	-.472
				2.00	-.776	-.881	-1.000	-.881
			2.00	1.00	-.716	-.330	-.511	-.339
				2.00	.159	.095	.129	.094
		2.00	1.00	1.00	1.401	1.811	2.055	1.422
				2.00	-.301	-.549	-.592	-.549
			2.00	1.00	.372	.229	.330	.224
				2.00	.410	.325	.419	.312
3.00	1.00	1.00	1.00	1.00	-.262	-.082	-.272	-.082
				2.00	-.272	-.241	-.427	-.251
			2.00	1.00	1.242	.937	1.626	.850
				2.00	-.262	-.512	-.580	-.512
		2.00	1.00	1.00	-.317	-.563	-.715	-.563
				2.00	-.039	-.199	-.206	-.199
			2.00	1.00	-.078	-.280	-.298	-.280
				2.00	-.012	-.108	-.110	-.108
	2.00	1.00	1.00	1.00	.175	.193	.263	.187
				2.00	-.117	-.343	-.366	-.343
			2.00	1.00	-1.284	-1.133	-1.754	-1.133
				2.00	.780	1.664	1.858	1.212
		2.00	1.00	1.00	.853	2.228	2.497	1.460
				2.00	-.021	-.145	-.148	-.145
			2.00	1.00	-.330	-.574	-.734	-.574
				2.00	-.057	-.238	-.250	-.238

a. Model: Poisson

b. Design: Constant + Recur1yr123 + HIBP + DM + Age2 + DPaverage + Recur1yr123 * HIBP + DM * Age2 + DM * DPaverage + Recur1yr123 * DM + HIBP * DM + HIBP * Age2 + HIBP * DPaverage + Age2 * DPaverage + Recur1yr123 * Age2 + Recur1yr123 * DPaverage

Parameter Estimates[b,c]

Parameter	Estimate	Std. Error	Z	Sig.	95% Confidence Interval	
					Lower Bound	Upper Bound
Constant	-2.870	1.296	-2.215	.027	-5.411	-.330
[Recur1yr123 = 1.00]	4.500	1.264	3.560	.000	2.022	6.978
[Recur1yr123 = 2.00]	3.334	1.326	2.515	.012	.736	5.933
[Recur1yr123 = 3.00]	0[a]	.	.	.	.	.
[HIBP = 1.00]	-1.576	.889	-1.774	.076	-3.318	.165
[HIBP = 2.00]	0[a]	.	.	.	.	.
[DM = 1.00]	1.356	1.141	1.189	.235	-.880	3.591
[DM = 2.00]	0[a]	.	.	.	.	.
[Age2 = 1.00]	-.994	.795	-1.251	.211	-2.551	.563
[Age2 = 2.00]	0[a]	.	.	.	.	.
[DPaverage = 1.00]	1.760	.844	2.085	.037	.106	3.415
[DPaverage = 2.00]	0[a]	.	.	.	.	.
[Recur1yr123 = 1.00] * [HIBP = 1.00]	-.212	.744	-.285	.776	-1.670	1.246
[Recur1yr123 = 1.00] * [HIBP = 2.00]	0[a]	.	.	.	.	.
[Recur1yr123 = 2.00] * [HIBP = 1.00]	-.401	.823	-.488	.626	-2.014	1.212
[Recur1yr123 = 2.00] * [HIBP = 2.00]	0[a]	.	.	.	.	.
[Recur1yr123 = 3.00] * [HIBP = 1.00]	0[a]	.	.	.	.	.
[Recur1yr123 = 3.00] * [HIBP = 2.00]	0[a]	.	.	.	.	.
[DM = 1.00] * [Age2 = 1.00]	.366	.434	.843	.399	-.485	1.217
[DM = 1.00] * [Age2 = 2.00]	0[a]	.	.	.	.	.
[DM = 2.00] * [Age2 = 1.00]	0[a]	.	.	.	.	.
[DM = 2.00] * [Age2 = 2.00]	0[a]	.	.	.	.	.
[DM = 1.00] * [DPaverage = 1.00]	.005	.402	.011	.991	-.783	.792

[DM = 1.00] * [DPaverage = 2.00]	0[a]	.	.	.	.	.
[DM = 2.00] * [DPaverage = 1.00]	0[a]	.	.	.	.	.
[DM = 2.00] * [DPaverage = 2.00]	0[a]	.	.	.	.	.
[Recur1yr123 = 1.00] * [DM = 1.00]	-.500	1.089	-.459	.646	-2.635	1.635
[Recur1yr123 = 1.00] * [DM = 2.00]	0[a]	.	.	.	.	.
[Recur1yr123 = 2.00] * [DM = 1.00]	-.776	1.159	-.669	.503	-3.047	1.496
[Recur1yr123 = 2.00] * [DM = 2.00]	0[a]	.	.	.	.	.
[Recur1yr123 = 3.00] * [DM = 1.00]	0[a]	.	.	.	.	.
[Recur1yr123 = 3.00] * [DM = 2.00]	0[a]	.	.	.	.	.
[HIBP = 1.00] * [DM = 1.00]	1.752	.439	3.995	.000	.892	2.612
[HIBP = 1.00] * [DM = 2.00]	0[a]	.	.	.	.	.
[HIBP = 2.00] * [DM = 1.00]	0[a]	.	.	.	.	.
[HIBP = 2.00] * [DM = 2.00]	0[a]	.	.	.	.	.
[HIBP = 1.00] * [Age2 = 1.00]	2.208	.307	7.198	.000	1.606	2.809
[HIBP = 1.00] * [Age2 = 2.00]	0[a]	.	.	.	.	.
[HIBP = 2.00] * [Age2 = 1.00]	0[a]	.	.	.	.	.
[HIBP = 2.00] * [Age2 = 2.00]	0[a]	.	.	.	.	.
[HIBP = 1.00] * [DPaverage = 1.00]	.138	.325	.425	.670	-.499	.775
[HIBP = 1.00] * [DPaverage = 2.00]	0[a]	.	.	.	.	.
[HIBP = 2.00] * [DPaverage = 1.00]	0[a]	.	.	.	.	.
[HIBP = 2.00] * [DPaverage = 2.00]	0[a]	.	.	.	.	.
[Age2 = 1.00] * [DPaverage = 1.00]	.185	.295	.626	.532	-.394	.763
[Age2 = 1.00] * [DPaverage = 2.00]	0[a]	.	.	.	.	.

[Age2 = 2.00] * [DPaverage = 1.00]	0[a]	.	.	.	.	.
[Age2 = 2.00] * [DPaverage = 2.00]	0[a]	.	.	.	.	.
[Recur1yr123 = 1.00] * [Age2 = 1.00]	-.329	.655	-.502	.616	-1.613	.955
[Recur1yr123 = 1.00] * [Age2 = 2.00]	0[a]	.	.	.	.	.
[Recur1yr123 = 2.00] * [Age2 = 1.00]	-.670	.732	-.916	.360	-2.104	.764
[Recur1yr123 = 2.00] * [Age2 = 2.00]	0[a]	.	.	.	.	.
[Recur1yr123 = 3.00] * [Age2 = 1.00]	0[a]	.	.	.	.	.
[Recur1yr123 = 3.00] * [Age2 = 2.00]	0[a]	.	.	.	.	.
[Recur1yr123 = 1.00] * [DPaverage = 1.00]	-1.194	.766	-1.559	.119	-2.696	.307
[Recur1yr123 = 1.00] * [DPaverage = 2.00]	0[a]	.	.	.	.	.
[Recur1yr123 = 2.00] * [DPaverage = 1.00]	-1.258	.824	-1.526	.127	-2.873	.357
[Recur1yr123 = 2.00] * [DPaverage = 2.00]	0[a]	.	.	.	.	.
[Recur1yr123 = 3.00] * [DPaverage = 1.00]	0[a]	.	.	.	.	.
[Recur1yr123 = 3.00] * [DPaverage = 2.00]	0[a]	.	.	.	.	.

a. This parameter is set to zero because it is redundant.

b. Model: Poisson

c. Design: Constant + Recur1yr123 + HIBP + DM + Age2 + DPaverage + Recur1yr123 * HIBP + DM * Age2 + DM * DPaverage + Recur1yr123 * DM + HIBP * DM + HIBP * Age2 + HIBP * DPaverage + Age2 * DPaverage + Recur1yr123 * Age2 + Recur1yr123 * DPaverage

Correlations of Parameter Estimates[a,b,c]

	Constant	[Recur1yr123 = 1.00]	[Recur1yr123 = 2.00]	[HIBP = 1.00]	[DM = 1.00]
Constant	1	-.964	-.927	-.151	-.703
[Recur1yr123 = 1.00]	-.964	1	.928	.124	.657
[Recur1yr123 = 2.00]	-.927	.928	1	.125	.634

[HIBP = 1.00]	-.151	.124	.125	1	-.156
[DM = 1.00]	-.703	.657	.634	-.156	1
[Age2 = 1.00]	-.173	.135	.141	-.391	.009
[DPaverage = 1.00]	-.554	.487	.473	.006	.103
[Recur1yr123 = 1.00] * [HIBP = 1.00]	.136	-.140	-.130	-.830	.195
[Recur1yr123 = 2.00] * [HIBP = 1.00]	.124	-.123	-.135	-.763	.177
[DM = 1.00] * [Age2 = 1.00]	.072	-.019	-.034	.262	-.109
[DM = 1.00] * [DPaverage = 1.00]	.191	-.053	-.059	.094	-.294
[Recur1yr123 = 1.00] * [DM = 1.00]	.667	-.692	-.640	.169	-.942
[Recur1yr123 = 2.00] * [DM = 1.00]	.632	-.630	-.686	.157	-.893
[HIBP = 1.00] * [DM = 1.00]	.057	-.015	-.023	-.461	-.079
[HIBP = 1.00] * [Age2 = 1.00]	.024	-.007	-.011	-.203	.042
[HIBP = 1.00] * [DPaverage = 1.00]	.042	-.015	-.019	-.316	.078
[Age2 = 1.00] * [DPaverage = 1.00]	.053	-.021	-.029	.145	.015
[Recur1yr123 = 1.00] * [Age2 = 1.00]	.144	-.149	-.138	.342	.035
[Recur1yr123 = 2.00] * [Age2 = 1.00]	.131	-.129	-.144	.311	.030
[Recur1yr123 = 1.00] * [DPaverage = 1.00]	.496	-.515	-.480	.010	-.005
[Recur1yr123 = 2.00] * [DPaverage = 1.00]	.470	-.470	-.511	.006	-.009

Correlations of Parameter Estimates[a,b,c]

	[Age2 = 1.00]	[DPaverage = 1.00]	[Recur1yr123 = 1.00] * [HIBP = 1.00]	[Recur1yr123 = 2.00] * [HIBP = 1.00]
Constant	-.173	-.554	.136	.124
[Recur1yr123 = 1.00]	.135	.487	-.140	-.123
[Recur1yr123 = 2.00]	.141	.473	-.130	-.135
[HIBP = 1.00]	-.391	.006	-.830	-.763
[DM = 1.00]	.009	.103	.195	.177
[Age2 = 1.00]	1	.005	.334	.304
[DPaverage = 1.00]	.005	1	.008	.007

[Recur1yr123 = 1.00] * [HIBP = 1.00]	.334	.008	1	.859
[Recur1yr123 = 2.00] * [HIBP = 1.00]	.304	.007	.859	1
[DM = 1.00] * [Age2 = 1.00]	-.433	.013	-.019	-.032
[DM = 1.00] * [DPaverage = 1.00]	.031	-.352	-.023	-.023
[Recur1yr123 = 1.00] * [DM = 1.00]	.023	-.025	-.211	-.181
[Recur1yr123 = 2.00] * [DM = 1.00]	.012	-.026	-.188	-.221
[HIBP = 1.00] * [DM = 1.00]	.220	.010	.027	.038
[HIBP = 1.00] * [Age2 = 1.00]	-.143	.006	.025	.047
[HIBP = 1.00] * [DPaverage = 1.00]	.150	-.074	.087	.084
[Age2 = 1.00] * [DPaverage = 1.00]	-.322	-.095	-.039	-.039
[Recur1yr123 = 1.00] * [Age2 = 1.00]	-.816	.013	-.414	-.353
[Recur1yr123 = 2.00] * [Age2 = 1.00]	-.740	.012	-.350	-.423
[Recur1yr123 = 1.00] * [DPaverage = 1.00]	.013	-.893	-.015	-.013
[Recur1yr123 = 2.00] * [DPaverage = 1.00]	.006	-.846	-.012	-.016

Correlations of Parameter Estimates[a,b,c]

	[DM = 1.00] * [Age2 = 1.00]	[DM = 1.00] * [DPaverage = 1.00]	[Recur1yr123 = 1.00] * [DM = 1.00]	[Recur1yr123 = 2.00] * [DM = 1.00]
Constant	.072	.191	.667	.632
[Recur1yr123 = 1.00]	-.019	-.053	-.692	-.630
[Recur1yr123 = 2.00]	-.034	-.059	-.640	-.686
[HIBP = 1.00]	.262	.094	.169	.157
[DM = 1.00]	-.109	-.294	-.942	-.893
[Age2 = 1.00]	-.433	.031	.023	.012
[DPaverage = 1.00]	.013	-.352	-.025	-.026
[Recur1yr123 = 1.00] * [HIBP = 1.00]	-.019	-.023	-.211	-.188
[Recur1yr123 = 2.00] * [HIBP = 1.00]	-.032	-.023	-.181	-.221
[DM = 1.00] * [Age2 = 1.00]	1	-.037	.024	.045

[DM = 1.00] * [DPaverage = 1.00]	-.037	1	.073	.073
[Recur1yr123 = 1.00] * [DM = 1.00]	.024	.073	1	.906
[Recur1yr123 = 2.00] * [DM = 1.00]	.045	.073	.906	1
[HIBP = 1.00] * [DM = 1.00]	-.480	-.027	.010	.018
[HIBP = 1.00] * [Age2 = 1.00]	-.261	.013	-.008	-.015
[HIBP = 1.00] * [DPaverage = 1.00]	.010	-.264	-.019	-.019
[Age2 = 1.00] * [DPaverage = 1.00]	.001	-.050	-.004	-.004
[Recur1yr123 = 1.00] * [Age2 = 1.00]	.025	-.005	-.040	-.035
[Recur1yr123 = 2.00] * [Age2 = 1.00]	.039	-.006	-.034	-.041
[Recur1yr123 = 1.00] * [DPaverage = 1.00]	-.001	.017	.001	.001
[Recur1yr123 = 2.00] * [DPaverage = 1.00]	-.001	.031	.002	.002

Correlations of Parameter Estimates[a,b,c]

	[HIBP = 1.00] * [DM = 1.00]	[HIBP = 1.00] * [Age2 = 1.00]	[HIBP = 1.00] * [DPaverage = 1.00]	[Age2 = 1.00] * [DPaverage = 1.00]
Constant	.057	.024	.042	.053
[Recur1yr123 = 1.00]	-.015	-.007	-.015	-.021
[Recur1yr123 = 2.00]	-.023	-.011	-.019	-.029
[HIBP = 1.00]	-.461	-.203	-.316	.145
[DM = 1.00]	-.079	.042	.078	.015
[Age2 = 1.00]	.220	-.143	.150	-.322
[DPaverage = 1.00]	.010	.006	-.074	-.095
[Recur1yr123 = 1.00] * [HIBP = 1.00]	.027	.025	.087	-.039
[Recur1yr123 = 2.00] * [HIBP = 1.00]	.038	.047	.084	-.039
[DM = 1.00] * [Age2 = 1.00]	-.480	-.261	.010	.001
[DM = 1.00] * [DPaverage = 1.00]	-.027	.013	-.264	-.050
[Recur1yr123 = 1.00] * [DM = 1.00]	.010	-.008	-.019	-.004

[Recur1yr123 = 2.00] * [DM = 1.00]	.018	-.015	-.019	-.004
[HIBP = 1.00] * [DM = 1.00]	1	.080	.007	.002
[HIBP = 1.00] * [Age2 = 1.00]	.080	1	-.029	-.010
[HIBP = 1.00] * [DPaverage = 1.00]	.007	-.029	1	-.451
[Age2 = 1.00] * [DPaverage = 1.00]	.002	-.010	-.451	1
[Recur1yr123 = 1.00] * [Age2 = 1.00]	-.013	.005	-.040	.088
[Recur1yr123 = 2.00] * [Age2 = 1.00]	-.022	.006	-.039	.084
[Recur1yr123 = 1.00] * [DPaverage = 1.00]	.000	-.001	.010	.019
[Recur1yr123 = 2.00] * [DPaverage = 1.00]	-.001	-.002	.021	.040

Correlations of Parameter Estimates[a,b,c]

	[Recur1yr123 = 1.00] * [Age2 = 1.00]	[Recur1yr123 = 2.00] * [Age2 = 1.00]	[Recur1yr123 = 1.00] * [DPaverage = 1.00]	[Recur1yr123 = 2.00] * [DPaverage = 1.00]
Constant	.144	.131	.496	.470
[Recur1yr123 = 1.00]	-.149	-.129	-.515	-.470
[Recur1yr123 = 2.00]	-.138	-.144	-.480	-.511
[HIBP = 1.00]	.342	.311	.010	.006
[DM = 1.00]	.035	.030	-.005	-.009
[Age2 = 1.00]	-.816	-.740	.013	.006
[DPaverage = 1.00]	.013	.012	-.893	-.846
[Recur1yr123 = 1.00] * [HIBP = 1.00]	-.414	-.350	-.015	-.012
[Recur1yr123 = 2.00] * [HIBP = 1.00]	-.353	-.423	-.013	-.016
[DM = 1.00] * [Age2 = 1.00]	.025	.039	-.001	-.001
[DM = 1.00] * [DPaverage = 1.00]	-.005	-.006	.017	.031
[Recur1yr123 = 1.00] * [DM = 1.00]	-.040	-.034	.001	.002
[Recur1yr123 = 2.00] * [DM = 1.00]	-.035	-.041	.001	.002
[HIBP = 1.00] * [DM = 1.00]	-.013	-.022	.000	-.001

[HIBP = 1.00] * [Age2 = 1.00]	.005	.006	-.001	-.002
[HIBP = 1.00] * [DPaverage = 1.00]	-.040	-.039	.010	.021
[Age2 = 1.00] * [DPaverage = 1.00]	.088	.084	.019	.040
[Recur1yr123 = 1.00] * [Age2 = 1.00]	1	.848	-.023	-.018
[Recur1yr123 = 2.00] * [Age2 = 1.00]	.848	1	-.019	-.023
[Recur1yr123 = 1.00] * [DPaverage = 1.00]	-.023	-.019	1	.902
[Recur1yr123 = 2.00] * [DPaverage = 1.00]	-.018	-.023	.902	1

a. Model: Poisson

b. Design: Constant + Recur1yr123 + HIBP + DM + Age2 + DPaverage + Recur1yr123 * HIBP + DM * Age2 + DM * DPaverage + Recur1yr123 * DM + HIBP * DM + HIBP * Age2 + HIBP * DPaverage + Age2 * DPaverage + Recur1yr123 * Age2 + Recur1yr123 * DPaverage

c. Redundant parameters are not displayed.

Covariances of Parameter Estimates[a,b,c]

	Constant	[Recur1yr123 = 1.00]	[Recur1yr123 = 2.00]	[HIBP = 1.00]	[DM = 1.00]
Constant	1.680	-1.579	-1.594	-.174	-1.039
[Recur1yr123 = 1.00]	-1.579	1.598	1.555	.139	.947
[Recur1yr123 = 2.00]	-1.594	1.555	1.758	.147	.959
[HIBP = 1.00]	-.174	.139	.147	.790	-.158
[DM = 1.00]	-1.039	.947	.959	-.158	1.301
[Age2 = 1.00]	-.179	.135	.148	-.276	.008
[DPaverage = 1.00]	-.607	.520	.529	.005	.100
[Recur1yr123 = 1.00] * [HIBP = 1.00]	.131	-.132	-.128	-.549	.165
[Recur1yr123 = 2.00] * [HIBP = 1.00]	.132	-.128	-.147	-.558	.167
[DM = 1.00] * [Age2 = 1.00]	.041	-.011	-.020	.101	-.054
[DM = 1.00] * [DPaverage = 1.00]	.100	-.027	-.032	.033	-.135
[Recur1yr123 = 1.00] * [DM = 1.00]	.941	-.953	-.924	.163	-1.170

[Recur1yr123 = 2.00] * [DM = 1.00]	.949	-.923	-1.054	.161	-1.181
[HIBP = 1.00] * [DM = 1.00]	.033	-.008	-.014	-.180	-.039
[HIBP = 1.00] * [Age2 = 1.00]	.010	-.003	-.004	-.055	.015
[HIBP = 1.00] * [DPaverage = 1.00]	.018	-.006	-.008	-.091	.029
[Age2 = 1.00] * [DPaverage = 1.00]	.020	-.008	-.011	.038	.005
[Recur1yr123 = 1.00] * [Age2 = 1.00]	.123	-.124	-.120	.199	.026
[Recur1yr123 = 2.00] * [Age2 = 1.00]	.124	-.119	-.140	.202	.025
[Recur1yr123 = 1.00] * [DPaverage = 1.00]	.493	-.499	-.488	.007	-.004
[Recur1yr123 = 2.00] * [DPaverage = 1.00]	.502	-.490	-.559	.004	-.008

Covariances of Parameter Estimates[a,b,c]

	[Age2 = 1.00]	[DPaverage = 1.00]	[Recur1yr123 = 1.00] * [HIBP = 1.00]	[Recur1yr123 = 2.00] * [HIBP = 1.00]
Constant	-.179	-.607	.131	.132
[Recur1yr123 = 1.00]	.135	.520	-.132	-.128
[Recur1yr123 = 2.00]	.148	.529	-.128	-.147
[HIBP = 1.00]	-.276	.005	-.549	-.558
[DM = 1.00]	.008	.100	.165	.167
[Age2 = 1.00]	.631	.004	.197	.199
[DPaverage = 1.00]	.004	.713	.005	.005
[Recur1yr123 = 1.00] * [HIBP = 1.00]	.197	.005	.553	.526
[Recur1yr123 = 2.00] * [HIBP = 1.00]	.199	.005	.526	.677
[DM = 1.00] * [Age2 = 1.00]	-.149	.005	-.006	-.011
[DM = 1.00] * [DPaverage = 1.00]	.010	-.119	-.007	-.008
[Recur1yr123 = 1.00] * [DM = 1.00]	.020	-.023	-.171	-.162
[Recur1yr123 = 2.00] * [DM = 1.00]	.011	-.025	-.162	-.211
[HIBP = 1.00] * [DM = 1.00]	.077	.004	.009	.014
[HIBP = 1.00] * [Age2 = 1.00]	-.035	.002	.006	.012

[HIBP = 1.00] * [DPaverage = 1.00]	.039	-.020	.021	.022
[Age2 = 1.00] * [DPaverage = 1.00]	-.075	-.024	-.009	-.009
[Recur1yr123 = 1.00] * [Age2 = 1.00]	-.425	.007	-.202	-.190
[Recur1yr123 = 2.00] * [Age2 = 1.00]	-.430	.007	-.191	-.255
[Recur1yr123 = 1.00] * [DPaverage = 1.00]	.008	-.578	-.009	-.008
[Recur1yr123 = 2.00] * [DPaverage = 1.00]	.004	-.589	-.008	-.011

Covariances of Parameter Estimates[a,b,c]

	[DM = 1.00] * [Age2 = 1.00]	[DM = 1.00] * [DPaverage = 1.00]	[Recur1yr123 = 1.00] * [DM = 1.00]	[Recur1yr123 = 2.00] * [DM = 1.00]
Constant	.041	.100	.941	.949
[Recur1yr123 = 1.00]	-.011	-.027	-.953	-.923
[Recur1yr123 = 2.00]	-.020	-.032	-.924	-1.054
[HIBP = 1.00]	.101	.033	.163	.161
[DM = 1.00]	-.054	-.135	-1.170	-1.181
[Age2 = 1.00]	-.149	.010	.020	.011
[DPaverage = 1.00]	.005	-.119	-.023	-.025
[Recur1yr123 = 1.00] * [HIBP = 1.00]	-.006	-.007	-.171	-.162
[Recur1yr123 = 2.00] * [HIBP = 1.00]	-.011	-.008	-.162	-.211
[DM = 1.00] * [Age2 = 1.00]	.188	-.006	.011	.023
[DM = 1.00] * [DPaverage = 1.00]	-.006	.162	.032	.034
[Recur1yr123 = 1.00] * [DM = 1.00]	.011	.032	1.187	1.144
[Recur1yr123 = 2.00] * [DM = 1.00]	.023	.034	1.144	1.343
[HIBP = 1.00] * [DM = 1.00]	-.091	-.005	.005	.009
[HIBP = 1.00] * [Age2 = 1.00]	-.035	.002	-.003	-.005
[HIBP = 1.00] * [DPaverage = 1.00]	.001	-.034	-.007	-.007
[Age2 = 1.00] * [DPaverage = 1.00]	.000	-.006	-.001	-.001

[Recur1yr123 = 1.00] * [Age2 = 1.00]	.007	-.001	-.029	-.027
[Recur1yr123 = 2.00] * [Age2 = 1.00]	.012	-.002	-.027	-.035
[Recur1yr123 = 1.00] * [DPaverage = 1.00]	.000	.005	.001	.001
[Recur1yr123 = 2.00] * [DPaverage = 1.00]	.000	.010	.002	.002

Covariances of Parameter Estimates[a,b,c]

	[HIBP = 1.00] * [DM = 1.00]	[HIBP = 1.00] * [Age2 = 1.00]	[HIBP = 1.00] * [DPaverage = 1.00]	[Age2 = 1.00] * [DPaverage = 1.00]
Constant	.033	.010	.018	.020
[Recur1yr123 = 1.00]	-.008	-.003	-.006	-.008
[Recur1yr123 = 2.00]	-.014	-.004	-.008	-.011
[HIBP = 1.00]	-.180	-.055	-.091	.038
[DM = 1.00]	-.039	.015	.029	.005
[Age2 = 1.00]	.077	-.035	.039	-.075
[DPaverage = 1.00]	.004	.002	-.020	-.024
[Recur1yr123 = 1.00] * [HIBP = 1.00]	.009	.006	.021	-.009
[Recur1yr123 = 2.00] * [HIBP = 1.00]	.014	.012	.022	-.009
[DM = 1.00] * [Age2 = 1.00]	-.091	-.035	.001	.000
[DM = 1.00] * [DPaverage = 1.00]	-.005	.002	-.034	-.006
[Recur1yr123 = 1.00] * [DM = 1.00]	.005	-.003	-.007	-.001
[Recur1yr123 = 2.00] * [DM = 1.00]	.009	-.005	-.007	-.001
[HIBP = 1.00] * [DM = 1.00]	.192	.011	.001	.000
[HIBP = 1.00] * [Age2 = 1.00]	.011	.094	-.003	-.001
[HIBP = 1.00] * [DPaverage = 1.00]	.001	-.003	.106	-.043
[Age2 = 1.00] * [DPaverage = 1.00]	.000	-.001	-.043	.087
[Recur1yr123 = 1.00] * [Age2 = 1.00]	-.004	.001	-.009	.017
[Recur1yr123 = 2.00] * [Age2 = 1.00]	-.007	.001	-.009	.018

[Recur1yr123 = 1.00] * [DPaverage = 1.00]	.000	.000	.002	.004
[Recur1yr123 = 2.00] * [DPaverage = 1.00]	.000	-.001	.006	.010

Covariances of Parameter Estimates[a,b,c]

	[Recur1yr123 = 1.00] * [Age2 = 1.00]	[Recur1yr123 = 2.00] * [Age2 = 1.00]	[Recur1yr123 = 1.00] * [DPaverage = 1.00]	[Recur1yr123 = 2.00] * [DPaverage = 1.00]
Constant	.123	.124	.493	.502
[Recur1yr123 = 1.00]	-.124	-.119	-.499	-.490
[Recur1yr123 = 2.00]	-.120	-.140	-.488	-.559
[HIBP = 1.00]	.199	.202	.007	.004
[DM = 1.00]	.026	.025	-.004	-.008
[Age2 = 1.00]	-.425	-.430	.008	.004
[DPaverage = 1.00]	.007	.007	-.578	-.589
[Recur1yr123 = 1.00] * [HIBP = 1.00]	-.202	-.191	-.009	-.008
[Recur1yr123 = 2.00] * [HIBP = 1.00]	-.190	-.255	-.008	-.011
[DM = 1.00] * [Age2 = 1.00]	.007	.012	.000	.000
[DM = 1.00] * [DPaverage = 1.00]	-.001	-.002	.005	.010
[Recur1yr123 = 1.00] * [DM = 1.00]	-.029	-.027	.001	.002
[Recur1yr123 = 2.00] * [DM = 1.00]	-.027	-.035	.001	.002
[HIBP = 1.00] * [DM = 1.00]	-.004	-.007	.000	.000
[HIBP = 1.00] * [Age2 = 1.00]	.001	.001	.000	-.001
[HIBP = 1.00] * [DPaverage = 1.00]	-.009	-.009	.002	.006
[Age2 = 1.00] * [DPaverage = 1.00]	.017	.018	.004	.010
[Recur1yr123 = 1.00] * [Age2 = 1.00]	.429	.406	-.011	-.010
[Recur1yr123 = 2.00] * [Age2 = 1.00]	.406	.535	-.011	-.014
[Recur1yr123 = 1.00] * [DPaverage = 1.00]	-.011	-.011	.587	.569
[Recur1yr123 = 2.00] * [DPaverage = 1.00]	-.010	-.014	.569	.679

a. Model: Poisson

b. Design: Constant + Recur1yr123 + HIBP + DM + Age2 + DPaverage + Recur1yr123 * HIBP + DM * Age2 + DM * DPaverage + Recur1yr123 * DM + HIBP * DM + HIBP * Age2 + HIBP * DPaverage + Age2 * DPaverage + Recur1yr123 * Age2 + Recur1yr123 * DPaverage

c. Redundant parameters are not displayed.

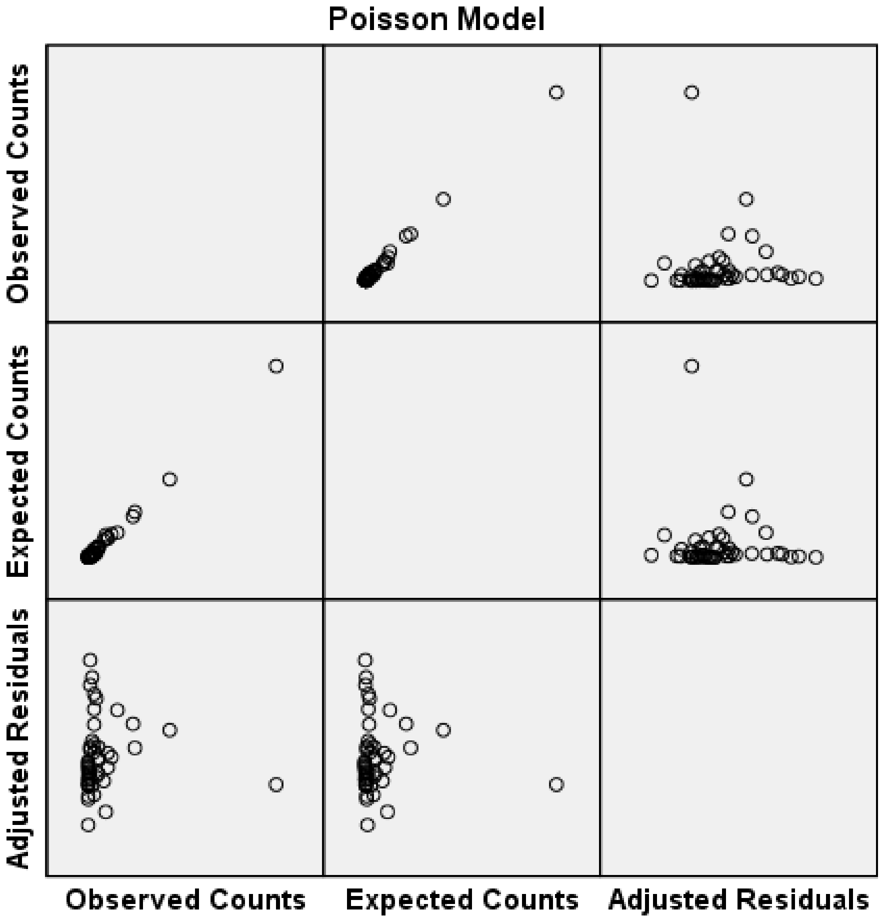

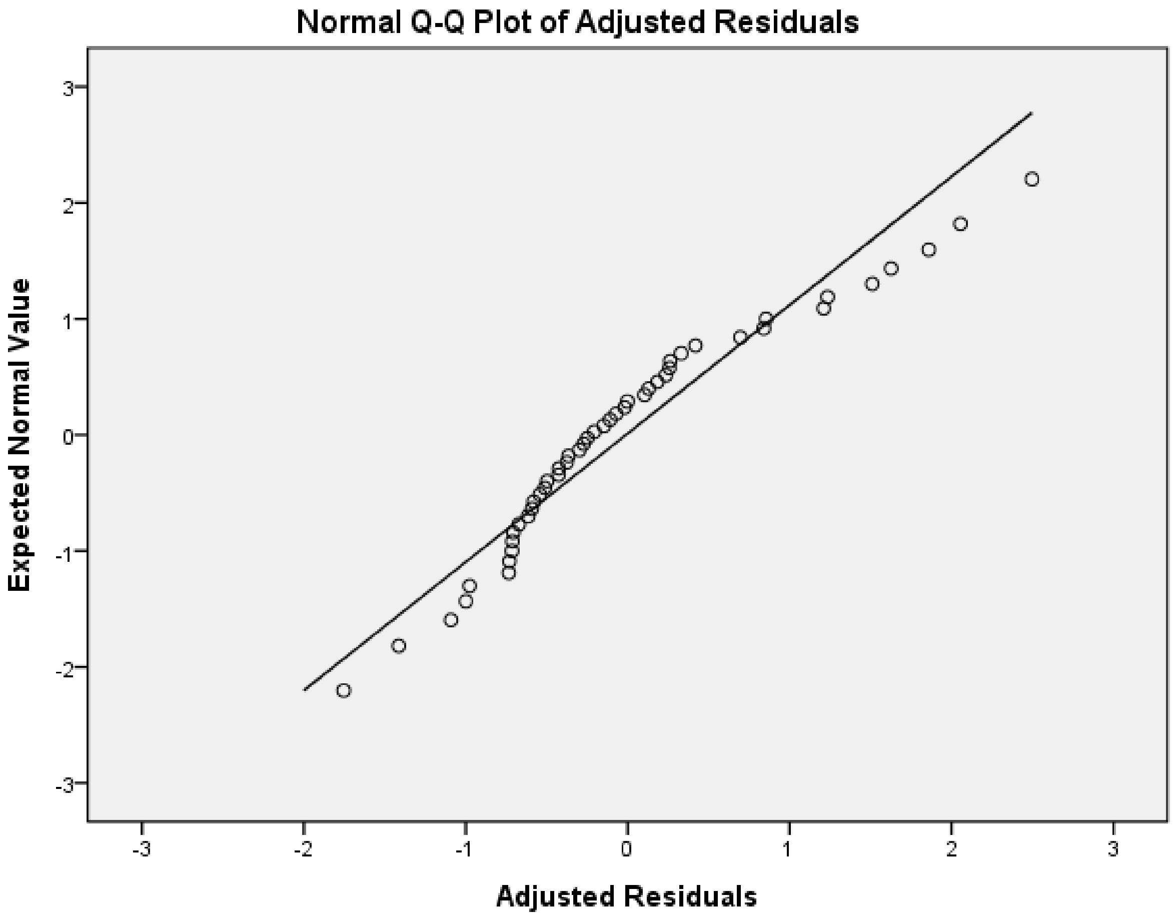
Normal Q-Q Plot of Adjusted Residuals
Expected Normal Value
Adjusted Residuals
3
2
1
0
-1
-2
-3
-3
-2
-1
0
1
2
3

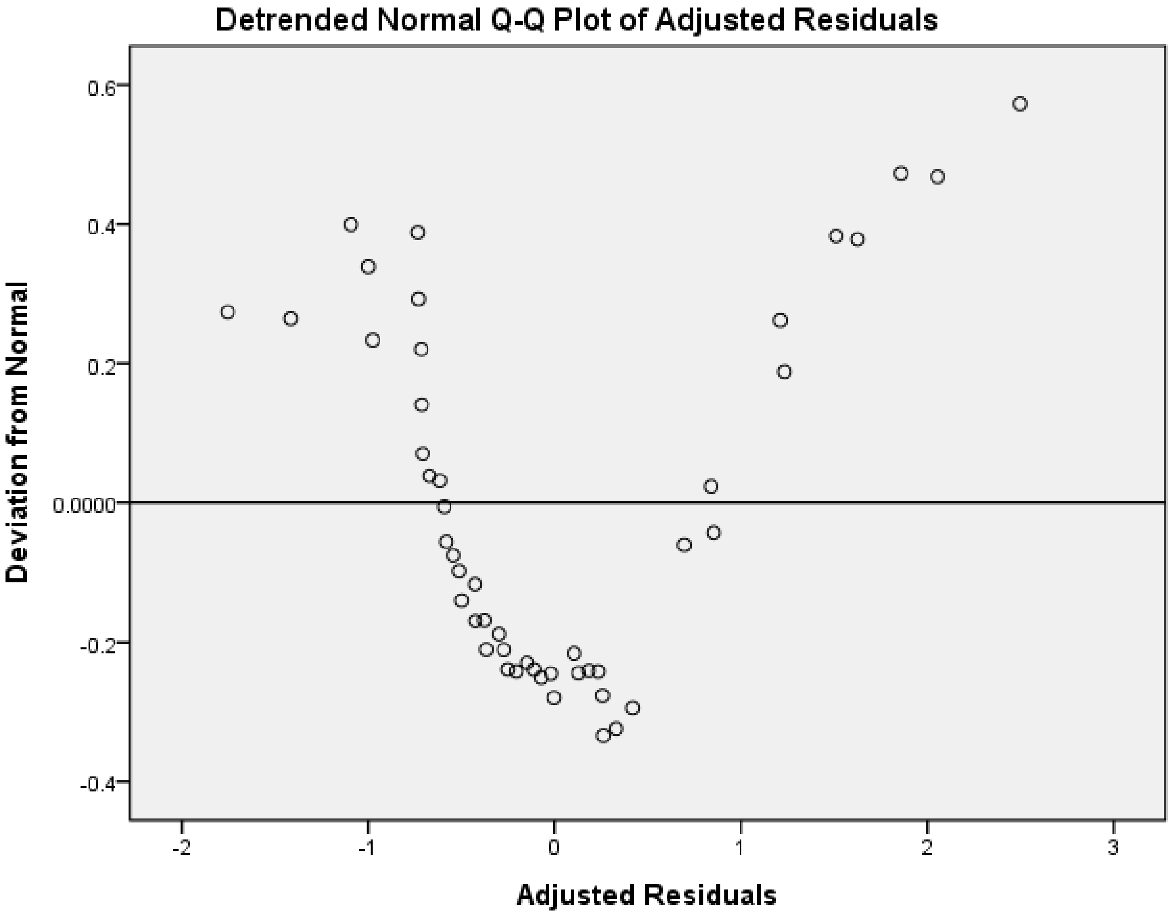


```
GENLOG Recur1yr123 BY HIBP DM Age2 DPaverage
  /MODEL=MULTINOMIAL
  /PRINT=FREQ RESID ADJRESID ZRESID DEV ESTIM CORR COV
  /PLOT=RESID(ADJRESID) NORMPROB(ADJRESID)
  /CRITERIA=CIN(95) ITERATE(20) CONVERGE(0.001) DELTA(.5)
  /DESIGN Recur1yr123 Recur1yr123*HIBP Recur1yr123*DM Recur1yr123*Age2
Recur1yr123*DPaverage.
```

logit Loglinear female

Notes

Output Created		14:4월:202319시 58분 20초
Comments		
Input	Data	C:\Users\guzi9\OneDrive\바탕화면\자율\result\ex3.sav

	Active Dataset	DataSet1
	Filter	sex = 2 (FILTER)
	Weight	<none>
	Split File	<none>
	N of Rows in Working Data File	309
Missing Value Handling	Definition of Missing	User-defined missing values are treated as missing.
	Cases Used	Statistics are based on all cases with valid data for all variables in the model.
Syntax		GENLOG Recur1yr123 BY HIBP DM Age2 DPaverage<br>/MODEL=MULTINOMIAL<br>/PRINT=FREQ RESID ADJRESID ZRESID DEV ESTIM CORR COV<br>/PLOT=RESID(ADJRESID) NORMPROB(ADJRESID)<br>/CRITERIA=CIN(95) ITERATE(20) CONVERGE(0.001) DELTA(.5)<br>/DESIGN Recur1yr123 Recur1yr123*HIBP Recur1yr123*DM Recur1yr123*Age2 Recur1yr123*DPaverage.
Resources	Processor Time	00 00:00:00.594
	Elapsed Time	00 00:00:00.595

[DataSet1] C:\Users\guzi9\OneDrive\바탕 화면\자율\result\ex3.sav

Data Information

		N
Cases	Valid	309
	Missing	0
	Weighted Valid	309
Cells	Defined Cells	48
	Structural Zeros	0
	Sampling Zeros	16
Categories	Recur1yr123	3
	HIBP	2

	DM	2
	Age2	2
	DPaverage	2

Convergence Information[b,c]

Maximum Number of Iterations	20
Converge Tolerance	.00100
Final Maximum Absolute Difference	3.32355E-5[a]
Final Maximum Relative Difference	2.19054E-5
Number of Iterations	6

a. The iteration converged because the maximum absolute changes of parameter estimates is less than the specified convergence criterion.

b. Model: Multinomial Logit

c. Design: Constant + Recur1yr123 + Recur1yr123 * HIBP + Recur1yr123 * DM + Recur1yr123 * Age2 + Recur1yr123 * DPaverage

Goodness-of-Fit Tests[a,b]

	Value	df	Sig.
Likelihood Ratio	20.712	22	.539
Pearson Chi-Square	18.932	22	.650

a. Model: Multinomial Logit

b. Design: Constant + Recur1yr123 + Recur1yr123 * HIBP + Recur1yr123 * DM + Recur1yr123 * Age2 + Recur1yr123 * DPaverage

Analysis of Dispersion[a,b]

	Entropy	Concentration	df
Model	3.780	.813	8

Residual	182.310	99.277	608
Total	186.090	100.091	616

a. Model: Multinomial Logit

b. Design: Constant + Recur1yr123 + Recur1yr123 * HIBP + Recur1yr123 * DM + Recur1yr123 * Age2 + Recur1yr123 * DPaverage

Measure of Association[a,b]

Entropy	.020
Concentration	.008

a. Model: Multinomial Logit

b. Design: Constant + Recur1yr123 + Recur1yr123 * HIBP + Recur1yr123 * DM + Recur1yr123 * Age2 + Recur1yr123 * DPaverage

Cell Counts and Residuals[a,b]

					Observed		Expected	
HIBP	DM	Age2	DPaverage	Recur1yr123	Count	%	Count	%
1.00	1.00	1.00	1.00	1.00	97	79.5%	98.822	81.0%
				2.00	15	12.3%	12.907	10.6%
				3.00	10	8.2%	10.271	8.4%
			2.00	1.00	42	87.5%	41.007	85.4%
				2.00	5	10.4%	5.703	11.9%
				3.00	1	2.1%	1.290	2.7%
		2.00	1.00	1.00	24	80.0%	23.842	79.5%
				2.00	3	10.0%	4.381	14.6%
				3.00	3	10.0%	1.777	5.9%
			2.00	1.00	9	69.2%	10.672	82.1%
				2.00	4	30.8%	2.088	16.1%
				3.00	0	.0%	.241	1.9%
	2.00	1.00	1.00	1.00	5	100.0%	4.047	80.9%
				2.00	0	.0%	.697	13.9%
				3.00	0	.0%	.256	5.1%

			2.00	1.00	3	100.0%	2.495	83.2%
				2.00	0	.0%	.457	15.2%
				3.00	0	.0%	.048	1.6%
		2.00	1.00	1.00	3	100.0%	2.330	77.7%
				2.00	0	.0%	.564	18.8%
				3.00	0	.0%	.106	3.5%
			2.00	1.00	1	100.0%	.786	78.6%
				2.00	0	.0%	.203	20.3%
				3.00	0	.0%	.011	1.1%
2.00	1.00	1.00	1.00	1.00	10	83.3%	9.662	80.5%
				2.00	1	8.3%	1.526	12.7%
				3.00	1	8.3%	.812	6.8%
			2.00	1.00	5	100.0%	4.189	83.8%
				2.00	0	.0%	.704	14.1%
				3.00	0	.0%	.107	2.1%
		2.00	1.00	1.00	23	85.2%	21.054	78.0%
				2.00	4	14.8%	4.676	17.3%
				3.00	0	.0%	1.269	4.7%
			2.00	1.00	12	75.0%	12.752	79.7%
				2.00	3	18.8%	3.016	18.8%
				3.00	1	6.3%	.233	1.5%
	2.00	1.00	1.00	1.00	2	40.0%	3.970	79.4%
				2.00	2	40.0%	.826	16.5%
				3.00	1	20.0%	.203	4.1%
			2.00	1.00	1	100.0%	.808	80.8%
				2.00	0	.0%	.179	17.9%
				3.00	0	.0%	.013	1.3%
		2.00	1.00	1.00	8	72.7%	8.273	75.2%
				2.00	3	27.3%	2.423	22.0%
				3.00	0	.0%	.304	2.8%
			2.00	1.00	5	71.4%	5.291	75.6%
				2.00	2	28.6%	1.650	23.6%
				3.00	0	.0%	.059	.8%

Cell Counts and Residuals[a,b]

HIBP	DM	Age2	DPaverage	Recur1yr123	Residual	Standardized Residual	Adjusted Residual	Deviance
1.00	1.00	1.00	1.00	1.00	-1.822	-.420	-.959	-1.900
				2.00	2.093	.616	1.216	2.123

				3.00	-.271	-.088	-.286	-.731
			2.00	1.00	.993	.406	.642	1.418
				2.00	-.703	-.314	-.484	-1.147
				3.00	-.290	-.259	-.460	-.714
		2.00	1.00	1.00	.158	.071	.107	.563
				2.00	-1.381	-.714	-1.041	-1.507
				3.00	1.223	.946	1.605	1.772
			2.00	1.00	-1.672	-1.209	-1.453	-1.751
				2.00	1.912	1.445	1.751	2.281
				3.00	-.241	-.495	-.552	.000
	2.00	1.00	1.00	1.00	.953	1.085	1.212	1.454
				2.00	-.697	-.900	-.985	.000
				3.00	-.256	-.520	-.620	.000
			2.00	1.00	.505	.780	.833	1.052
				2.00	-.457	-.735	-.786	.000
				3.00	-.048	-.220	-.230	.000
		2.00	1.00	1.00	.670	.929	1.009	1.232
				2.00	-.564	-.834	-.906	.000
				3.00	-.106	-.331	-.359	.000
			2.00	1.00	.214	.521	.537	.693
				2.00	-.203	-.504	-.520	.000
				3.00	-.011	-.105	-.106	.000
2.00	1.00	1.00	1.00	1.00	.338	.246	.299	.829
				2.00	-.526	-.455	-.535	-.919
				3.00	.188	.216	.285	.645
			2.00	1.00	.811	.984	1.064	1.330
				2.00	-.704	-.905	-.982	.000
				3.00	-.107	-.330	-.349	.000
		2.00	1.00	1.00	1.946	.904	1.310	2.016
				2.00	-.676	-.344	-.496	-1.118
				3.00	-1.269	-1.154	-1.759	.000
			2.00	1.00	-.752	-.467	-.600	-1.208
				2.00	-.016	-.010	-.013	-.177
				3.00	.767	1.602	1.794	1.708
	2.00	1.00	1.00	1.00	-1.970	-2.178	-2.463	-1.656
				2.00	1.174	1.413	1.590	1.880
				3.00	.797	1.803	2.092	1.784
			2.00	1.00	.192	.487	.500	.652

			2.00	-.179	-.467	-.480	.000
			3.00	-.013	-.113	-.114	.000
	2.00	1.00	1.00	-.273	-.191	-.245	-.733
			2.00	.577	.420	.543	1.132
			3.00	-.304	-.559	-.692	.000
		2.00	1.00	-.291	-.256	-.307	-.752
			2.00	.350	.312	.375	.877
			3.00	-.059	-.244	-.256	.000

a. Model: Multinomial Logit

b. Design: Constant + Recur1yr123 + Recur1yr123 * HIBP + Recur1yr123 * DM + Recur1yr123 * Age2 + Recur1yr123 * DPaverage

Parameter Estimates[c,d]

Parameter		Estimate	Std. Error	Z	Sig.	95% Confidence Interval	
						Lower Bound	Upper Bound
Constant	[HIBP = 1.00] * [DM = 1.00] * [Age2 = 1.00] * [DPaverage = 1.00]	2.329[a]					
	[HIBP = 1.00] * [DM = 1.00] * [Age2 = 1.00] * [DPaverage = 2.00]	.255[a]					
	[HIBP = 1.00] * [DM = 1.00] * [Age2 = 2.00] * [DPaverage = 1.00]	.575[a]					
	[HIBP = 1.00] * [DM = 1.00] * [Age2 = 2.00] * [DPaverage = 2.00]	-1.424[a]					
	[HIBP = 1.00] * [DM = 2.00] * [Age2 = 1.00] * [DPaverage = 1.00]	-1.361[a]					
	[HIBP = 1.00] * [DM = 2.00] * [Age2 = 1.00] * [DPaverage = 2.00]	-3.040[a]					
	[HIBP = 1.00] * [DM = 2.00] * [Age2 = 2.00] * [DPaverage = 1.00]	-2.245[a]					

[HIBP = 1.00] * [DM = 2.00] * [Age2 = 2.00] * [DPaverage = 2.00]	-4.527[a]					
[HIBP = 2.00] * [DM = 1.00] * [Age2 = 1.00] * [DPaverage = 1.00]	-.208[a]					
[HIBP = 2.00] * [DM = 1.00] * [Age2 = 1.00] * [DPaverage = 2.00]	-2.239[a]					
[HIBP = 2.00] * [DM = 1.00] * [Age2 = 2.00] * [DPaverage = 1.00]	.239[a]					
[HIBP = 2.00] * [DM = 1.00] * [Age2 = 2.00] * [DPaverage = 2.00]	-1.458[a]					
[HIBP = 2.00] * [DM = 2.00] * [Age2 = 1.00] * [DPaverage = 1.00]	-1.592[a]					
[HIBP = 2.00] * [DM = 2.00] * [Age2 = 1.00] * [DPaverage = 2.00]	-4.379[a]					
[HIBP = 2.00] * [DM = 2.00] * [Age2 = 2.00] * [DPaverage = 1.00]	-1.190[a]					
[HIBP = 2.00] * [DM = 2.00] * [Age2 = 2.00] * [DPaverage = 2.00]	-2.832[a]					
[Recur1yr123 = 1.00]	4.498	1.262	3.564	.000	2.025	6.972
[Recur1yr123 = 2.00]	3.333	1.324	2.517	.012	.738	5.928
[Recur1yr123 = 3.00]	0[b]	.	.	.	.	.
[Recur1yr123 = 1.00] * [HIBP = 1.00]	-.212	.743	-.285	.775	-1.669	1.245
[Recur1yr123 = 1.00] * [HIBP = 2.00]	0[b]	.	.	.	.	.
[Recur1yr123 = 2.00] * [HIBP = 1.00]	-.402	.822	-.489	.625	-2.013	1.209
[Recur1yr123 = 2.00] * [HIBP = 2.00]	0[b]	.	.	.	.	.
[Recur1yr123 = 3.00] * [HIBP = 1.00]	0[b]	.	.	.	.	.
[Recur1yr123 = 3.00] * [HIBP = 2.00]	0[b]	.	.	.	.	.

[Recur1yr123 = 1.00] * [DM = 1.00]	-.495	1.094	-.452	.651	-2.639	1.649
[Recur1yr123 = 1.00] * [DM = 2.00]	0[b]	.	.	.	.	.
[Recur1yr123 = 2.00] * [DM = 1.00]	-.771	1.163	-.663	.507	-3.050	1.508
[Recur1yr123 = 2.00] * [DM = 2.00]	0[b]	.	.	.	.	.
[Recur1yr123 = 3.00] * [DM = 1.00]	0[b]	.	.	.	.	.
[Recur1yr123 = 3.00] * [DM = 2.00]	0[b]	.	.	.	.	.
[Recur1yr123 = 1.00] * [Age2 = 1.00]	-.332	.652	-.510	.610	-1.610	.946
[Recur1yr123 = 1.00] * [Age2 = 2.00]	0[b]	.	.	.	.	.
[Recur1yr123 = 2.00] * [Age2 = 1.00]	-.674	.729	-.924	.355	-2.102	.755
[Recur1yr123 = 2.00] * [Age2 = 2.00]	0[b]	.	.	.	.	.
[Recur1yr123 = 3.00] * [Age2 = 1.00]	0[b]	.	.	.	.	.
[Recur1yr123 = 3.00] * [Age2 = 2.00]	0[b]	.	.	.	.	.
[Recur1yr123 = 1.00] * [DPaverage = 1.00]	-1.195	.766	-1.560	.119	-2.697	.306
[Recur1yr123 = 1.00] * [DPaverage = 2.00]	0[b]	.	.	.	.	.
[Recur1yr123 = 2.00] * [DPaverage = 1.00]	-1.258	.824	-1.526	.127	-2.873	.357
[Recur1yr123 = 2.00] * [DPaverage = 2.00]	0[b]	.	.	.	.	.
[Recur1yr123 = 3.00] * [DPaverage = 1.00]	0[b]	.	.	.	.	.
[Recur1yr123 = 3.00] * [DPaverage = 2.00]	0[b]	.	.	.	.	.

a. Constants are not parameters under the multinomial assumption. Therefore, their standard errors are not calculated.

b. This parameter is set to zero because it is redundant.

c. Model: Multinomial Logit

d. Design: Constant + Recur1yr123 + Recur1yr123 * HIBP + Recur1yr123 * DM + Recur1yr123 * Age2 + Recur1yr123 * DPaverage

Correlations of Parameter Estimates[a,b,c]

	[Recur1yr123 = 1.00]	[Recur1yr123 = 2.00]	[Recur1yr123 = 1.00] * [HIBP = 1.00]	[Recur1yr123 = 2.00] * [HIBP = 1.00]
[Recur1yr123 = 1.00]	1	.927	-.131	-.114
[Recur1yr123 = 2.00]	.927	1	-.121	-.127
[Recur1yr123 = 1.00] * [HIBP = 1.00]	-.131	-.121	1	.859
[Recur1yr123 = 2.00] * [HIBP = 1.00]	-.114	-.127	.859	1
[Recur1yr123 = 1.00] * [DM = 1.00]	-.689	-.636	-.228	-.196
[Recur1yr123 = 2.00] * [DM = 1.00]	-.628	-.683	-.204	-.235
[Recur1yr123 = 1.00] * [Age2 = 1.00]	-.157	-.145	-.403	-.344
[Recur1yr123 = 2.00] * [Age2 = 1.00]	-.135	-.150	-.340	-.414
[Recur1yr123 = 1.00] * [DPaverage = 1.00]	-.516	-.481	-.015	-.012
[Recur1yr123 = 2.00] * [DPaverage = 1.00]	-.471	-.512	-.012	-.016

Correlations of Parameter Estimates[a,b,c]

	[Recur1yr123 = 1.00] * [DM = 1.00]	[Recur1yr123 = 2.00] * [DM = 1.00]	[Recur1yr123 = 1.00] * [Age2 = 1.00]	[Recur1yr123 = 2.00] * [Age2 = 1.00]
[Recur1yr123 = 1.00]	-.689	-.628	-.157	-.135
[Recur1yr123 = 2.00]	-.636	-.683	-.145	-.150
[Recur1yr123 = 1.00] * [HIBP = 1.00]	-.228	-.204	-.403	-.340
[Recur1yr123 = 2.00] * [HIBP = 1.00]	-.196	-.235	-.344	-.414
[Recur1yr123 = 1.00] * [DM = 1.00]	1	.907	-.037	-.031
[Recur1yr123 = 2.00] * [DM = 1.00]	.907	1	-.032	-.038
[Recur1yr123 = 1.00] * [Age2 = 1.00]	-.037	-.032	1	.847
[Recur1yr123 = 2.00] * [Age2 = 1.00]	-.031	-.038	.847	1
[Recur1yr123 = 1.00] * [DPaverage = 1.00]	.000	.000	-.020	-.017

Correlations of Parameter Estimates[a,b,c]

	[Recur1yr123 = 1.00] * [DM = 1.00]	[Recur1yr123 = 2.00] * [DM = 1.00]	[Recur1yr123 = 1.00] * [Age2 = 1.00]	[Recur1yr123 = 2.00] * [Age2 = 1.00]
[Recur1yr123 = 1.00]	-.689	-.628	-.157	-.135
[Recur1yr123 = 2.00]	-.636	-.683	-.145	-.150
[Recur1yr123 = 1.00] * [HIBP = 1.00]	-.228	-.204	-.403	-.340
[Recur1yr123 = 2.00] * [HIBP = 1.00]	-.196	-.235	-.344	-.414
[Recur1yr123 = 1.00] * [DM = 1.00]	1	.907	-.037	-.031
[Recur1yr123 = 2.00] * [DM = 1.00]	.907	1	-.032	-.038
[Recur1yr123 = 1.00] * [Age2 = 1.00]	-.037	-.032	1	.847
[Recur1yr123 = 2.00] * [Age2 = 1.00]	-.031	-.038	.847	1
[Recur1yr123 = 1.00] * [DPaverage = 1.00]	.000	.000	-.020	-.017
[Recur1yr123 = 2.00] * [DPaverage = 1.00]	.001	.001	-.015	-.021

Correlations of Parameter Estimates[a,b,c]

	[Recur1yr123 = 1.00] * [DPaverage = 1.00]	[Recur1yr123 = 2.00] * [DPaverage = 1.00]
[Recur1yr123 = 1.00]	-.516	-.471
[Recur1yr123 = 2.00]	-.481	-.512
[Recur1yr123 = 1.00] * [HIBP = 1.00]	-.015	-.012
[Recur1yr123 = 2.00] * [HIBP = 1.00]	-.012	-.016
[Recur1yr123 = 1.00] * [DM = 1.00]	.000	.001
[Recur1yr123 = 2.00] * [DM = 1.00]	.000	.001
[Recur1yr123 = 1.00] * [Age2 = 1.00]	-.020	-.015
[Recur1yr123 = 2.00] * [Age2 = 1.00]	-.017	-.021

[Recur1yr123 = 1.00] * [DPaverage = 1.00]	1	.902
[Recur1yr123 = 2.00] * [DPaverage = 1.00]	.902	1

a. Model: Multinomial Logit

b. Design: Constant + Recur1yr123 + Recur1yr123 * HIBP + Recur1yr123 * DM + Recur1yr123 * Age2 + Recur1yr123 * DPaverage

c. Constants and redundant parameters are not displayed.

Covariances of Parameter Estimates[a,b,c]

	[Recur1yr123 = 1.00]	[Recur1yr123 = 2.00]	[Recur1yr123 = 1.00] * [HIBP = 1.00]	[Recur1yr123 = 2.00] * [HIBP = 1.00]
[Recur1yr123 = 1.00]	1.593	1.550	-.123	-.118
[Recur1yr123 = 2.00]	1.550	1.753	-.119	-.138
[Recur1yr123 = 1.00] * [HIBP = 1.00]	-.123	-.119	.553	.525
[Recur1yr123 = 2.00] * [HIBP = 1.00]	-.118	-.138	.525	.676
[Recur1yr123 = 1.00] * [DM = 1.00]	-.951	-.922	-.185	-.177
[Recur1yr123 = 2.00] * [DM = 1.00]	-.922	-1.052	-.176	-.225
[Recur1yr123 = 1.00] * [Age2 = 1.00]	-.129	-.125	-.196	-.184
[Recur1yr123 = 2.00] * [Age2 = 1.00]	-.124	-.145	-.184	-.248
[Recur1yr123 = 1.00] * [DPaverage = 1.00]	-.499	-.488	-.008	-.008
[Recur1yr123 = 2.00] * [DPaverage = 1.00]	-.490	-.559	-.007	-.011

Covariances of Parameter Estimates[a,b,c]

	[Recur1yr123 = 1.00] * [DM = 1.00]	[Recur1yr123 = 2.00] * [DM = 1.00]	[Recur1yr123 = 1.00] * [Age2 = 1.00]	[Recur1yr123 = 2.00] * [Age2 = 1.00]
[Recur1yr123 = 1.00]	-.951	-.922	-.129	-.124
[Recur1yr123 = 2.00]	-.922	-1.052	-.125	-.145

[Recur1yr123 = 1.00] * [HIBP = 1.00]	-.185	-.176	-.196	-.184
[Recur1yr123 = 2.00] * [HIBP = 1.00]	-.177	-.225	-.184	-.248
[Recur1yr123 = 1.00] * [DM = 1.00]	1.197	1.154	-.026	-.025
[Recur1yr123 = 2.00] * [DM = 1.00]	1.154	1.352	-.024	-.032
[Recur1yr123 = 1.00] * [Age2 = 1.00]	-.026	-.024	.425	.402
[Recur1yr123 = 2.00] * [Age2 = 1.00]	-.025	-.032	.402	.531
[Recur1yr123 = 1.00] * [DPaverage = 1.00]	.000	.000	-.010	-.009
[Recur1yr123 = 2.00] * [DPaverage = 1.00]	.001	.001	-.008	-.013

Covariances of Parameter Estimates[a,b,c]

	[Recur1yr123 = 1.00] * [DPaverage = 1.00]	[Recur1yr123 = 2.00] * [DPaverage = 1.00]
[Recur1yr123 = 1.00]	-.499	-.490
[Recur1yr123 = 2.00]	-.488	-.559
[Recur1yr123 = 1.00] * [HIBP = 1.00]	-.008	-.007
[Recur1yr123 = 2.00] * [HIBP = 1.00]	-.008	-.011
[Recur1yr123 = 1.00] * [DM = 1.00]	.000	.001
[Recur1yr123 = 2.00] * [DM = 1.00]	.000	.001
[Recur1yr123 = 1.00] * [Age2 = 1.00]	-.010	-.008
[Recur1yr123 = 2.00] * [Age2 = 1.00]	-.009	-.013
[Recur1yr123 = 1.00] * [DPaverage = 1.00]	.587	.569
[Recur1yr123 = 2.00] * [DPaverage = 1.00]	.569	.679

a. Model: Multinomial Logit

b. Design: Constant + Recur1yr123 + Recur1yr123 * HIBP + Recur1yr123 * DM + Recur1yr123 * Age2 + Recur1yr123 * DPaverage

c. Constants and redundant parameters are not displayed.

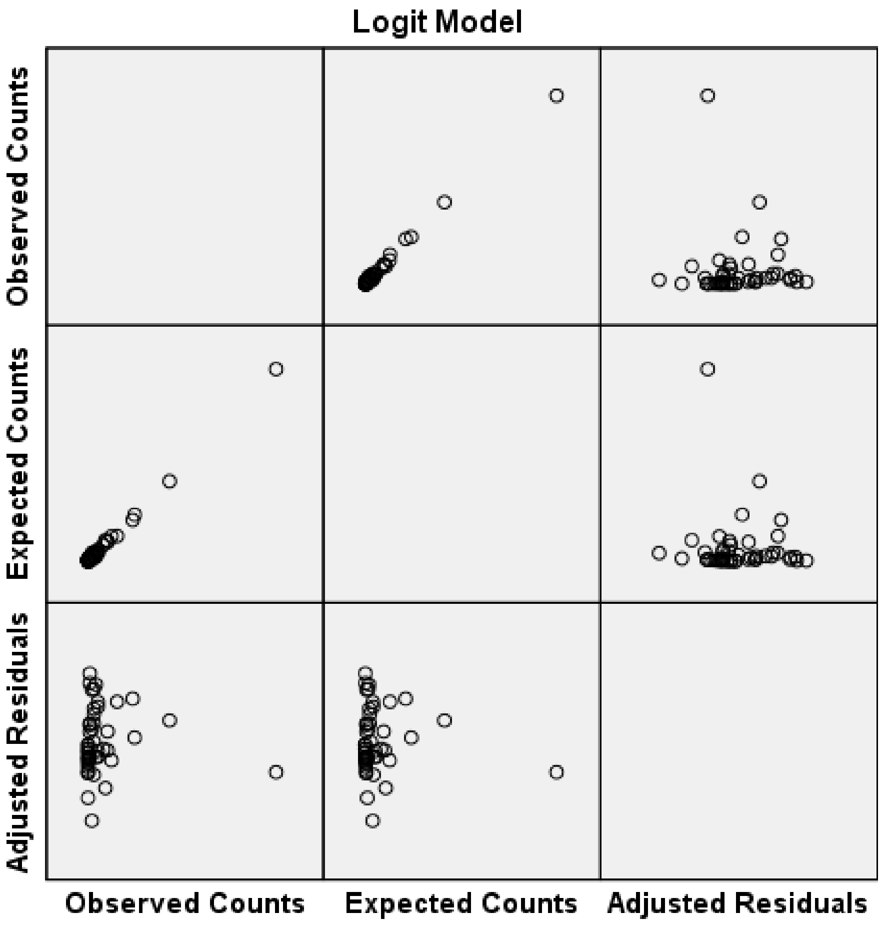

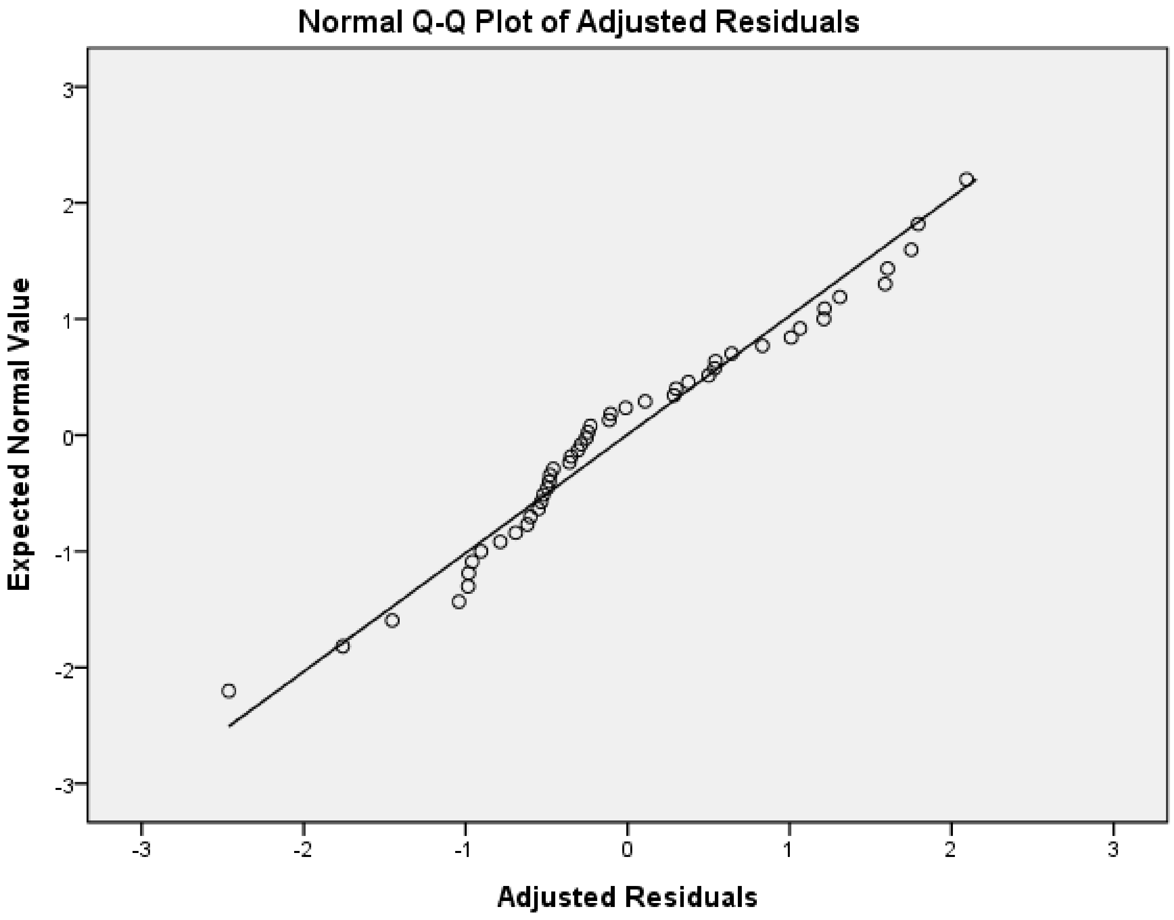
Normal Q-Q Plot of Adjusted Residuals
Expected Normal Value
Adjusted Residuals
3
2
1
0
-1
-2
-3
-3
-2
-1
0
1
2
3

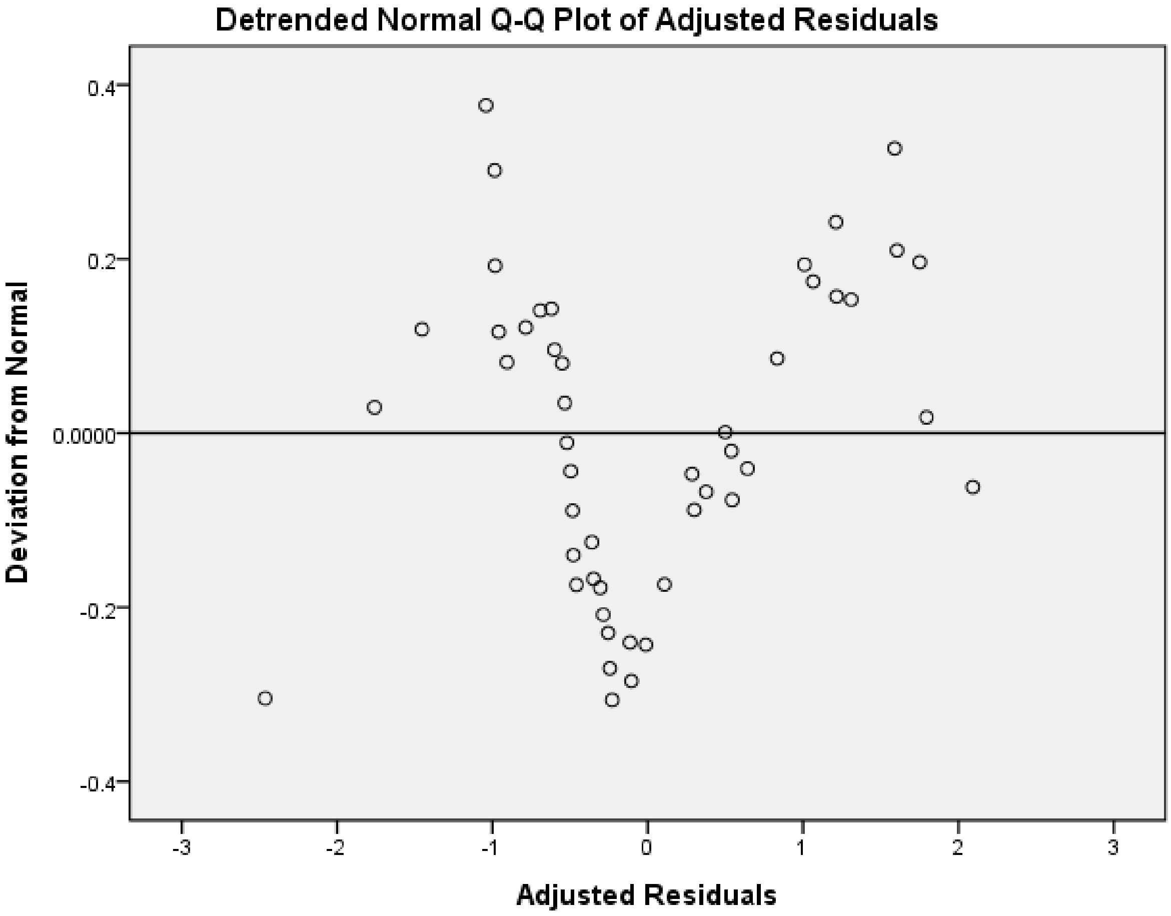


```
GENLOG Recur1yr123 BY HIBP DM Age2 DPaverage
  /MODEL=MULTINOMIAL
  /PRINT=FREQ RESID ADJRESID ZRESID DEV ESTIM CORR COV
  /PLOT=RESID(ADJRESID) NORMPROB(ADJRESID)
  /CRITERIA=CIN(95) ITERATE(20) CONVERGE(0.001) DELTA(.5)
  /DESIGN Recur1yr123 Recur1yr123*HIBP Recur1yr123*DM Recur1yr123*Age2
Recur1yr123*DPaverage.
```

General Loglinear

Notes

Output Created		26:6월:202321시 51분 00초
Comments		
Input	Data	C:\Users\guzi9\OneDrive\바탕 화면\자율\result\ex3.sav

	Active Dataset	DataSet1
	Filter	<none>
	Weight	<none>
	Split File	<none>
	N of Rows in Working Data File	432
Missing Value Handling	Definition of Missing	User-defined missing values are treated as missing.
	Cases Used	Statistics are based on all cases with valid data for all variables in the model.
Syntax		GENLOG Recur1yr123 BY HIBP DM Age2 DPaverage<br>/MODEL=MULTINOMIAL<br>/PRINT=FREQ RESID ADJRESID ZRESID DEV ESTIM CORR COV<br>/PLOT=RESID(ADJRESID) NORMPROB(ADJRESID)<br>/CRITERIA=CIN(95) ITERATE(20) CONVERGE(0.001) DELTA(.5)<br>/DESIGN Recur1yr123 Recur1yr123*HIBP Recur1yr123*DM Recur1yr123*Age2 Recur1yr123*DPaverage.
Resources	Processor Time	00 00:00:01.657
	Elapsed Time	00 00:00:01.404

[DataSet1] C:\Users\guzi9\OneDrive\바탕 화면\자율\result\ex3.sav

Data Information

		N
Cases	Valid	432
	Missing	0
	Weighted Valid	432
Cells	Defined Cells	48
	Structural Zeros	0
	Sampling Zeros	14
Categories	Recur1yr123	3
	HIBP	2

	DM	2
	Age2	2
	DPaverage	2

Convergence Information[b,c]

Maximum Number of Iterations	20
Converge Tolerance	.00100
Final Maximum Absolute Difference	.00013[a]
Final Maximum Relative Difference	.00011
Number of Iterations	6

a. The iteration converged because the maximum absolute changes of parameter estimates is less than the specified convergence criterion.

b. Model: Multinomial Logit

c. Design: Constant + Recur1yr123 + Recur1yr123 * HIBP + Recur1yr123 * DM + Recur1yr123 * Age2 + Recur1yr123 * DPaverage

Goodness-of-Fit Tests[a,b]

	Value	df	Sig.
Likelihood Ratio	22.655	22	.421
Pearson Chi-Square	19.463	22	.617

a. Model: Multinomial Logit

b. Design: Constant + Recur1yr123 + Recur1yr123 * HIBP + Recur1yr123 * DM + Recur1yr123 * Age2 + Recur1yr123 * DPaverage

Analysis of Dispersion[a,b]

	Entropy	Concentration	df
Model	2.967	.564	8

Residual	233.225	124.098	854
Total	236.193	124.662	862

a. Model: Multinomial Logit

b. Design: Constant + Recur1yr123 + Recur1yr123 * HIBP + Recur1yr123 * DM + Recur1yr123 * Age2 + Recur1yr123 * DPaverage

Measure of Association[a,b]

Entropy	.013
Concentration	.005

a. Model: Multinomial Logit

b. Design: Constant + Recur1yr123 + Recur1yr123 * HIBP + Recur1yr123 * DM + Recur1yr123 * Age2 + Recur1yr123 * DPaverage

Cell Counts and Residuals[a,b]

					Observed		Expected	
HIBP	DM	Age2	DPaverage	Recur1yr123	Count	%	Count	%
1.00	1.00	1.00	1.00	1.00	149	81.4%	150.883	82.4%
				2.00	23	12.6%	20.968	11.5%
				3.00	11	6.0%	11.149	6.1%
			2.00	1.00	51	89.5%	49.841	87.4%
				2.00	5	8.8%	6.189	10.9%
				3.00	1	1.8%	.970	1.7%
		2.00	1.00	1.00	29	82.9%	29.325	83.8%
				2.00	3	8.6%	3.822	10.9%
				3.00	3	8.6%	1.853	5.3%
			2.00	1.00	14	77.8%	15.886	88.3%
				2.00	4	22.2%	1.850	10.3%
				3.00	0	.0%	.264	1.5%
	2.00	1.00	1.00	1.00	8	100.0%	6.567	82.1%
				2.00	0	.0%	.952	11.9%
				3.00	0	.0%	.481	6.0%

			2.00	1.00	4	100.0%	3.482	87.0%
				2.00	0	.0%	.451	11.3%
				3.00	0	.0%	.067	1.7%
		2.00	1.00	1.00	3	100.0%	2.503	83.4%
				2.00	0	.0%	.340	11.3%
				3.00	0	.0%	.157	5.2%
			2.00	1.00	4	100.0%	3.515	87.9%
				2.00	0	.0%	.427	10.7%
				3.00	0	.0%	.058	1.5%
2.00	1.00	1.00	1.00	1.00	13	86.7%	11.862	79.1%
				2.00	1	6.7%	2.354	15.7%
				3.00	1	6.7%	.783	5.2%
			2.00	1.00	8	80.0%	8.370	83.7%
				2.00	2	20.0%	1.484	14.8%
				3.00	0	.0%	.146	1.5%
		2.00	1.00	1.00	30	88.2%	27.362	80.5%
				2.00	4	11.8%	5.093	15.0%
				3.00	0	.0%	1.545	4.5%
			2.00	1.00	19	82.6%	19.472	84.7%
				2.00	3	13.0%	3.239	14.1%
				3.00	1	4.3%	.290	1.3%
	2.00	1.00	1.00	1.00	3	42.9%	5.501	78.6%
				2.00	3	42.9%	1.139	16.3%
				3.00	1	14.3%	.360	5.1%
			2.00	1.00	3	100.0%	2.495	83.2%
				2.00	0	.0%	.462	15.4%
				3.00	0	.0%	.043	1.4%
		2.00	1.00	1.00	11	73.3%	11.998	80.0%
				2.00	3	20.0%	2.330	15.5%
				3.00	1	6.7%	.672	4.5%
			2.00	1.00	11	84.6%	10.940	84.2%
				2.00	2	15.4%	1.898	14.6%
				3.00	0	.0%	.161	1.2%

Cell Counts and Residuals[a,b]

HIBP	DM	Age2	DPaverage	Recur1yr123	Residual	Standardized Residual	Adjusted Residual	Deviance
1.00	1.00	1.00	1.00	1.00	-1.883	-.366	-.877	-1.934
				2.00	2.032	.471	1.045	2.062

				3.00	-.149	-.046	-.140	-.544
			2.00	1.00	1.159	.463	.687	1.531
				2.00	-1.189	-.506	-.749	-1.461
				3.00	.030	.030	.045	.245
		2.00	1.00	1.00	-.325	-.149	-.209	-.804
				2.00	-.822	-.446	-.597	-1.205
				3.00	1.147	.866	1.383	1.700
			2.00	1.00	-1.886	-1.380	-1.616	-1.881
				2.00	2.150	1.669	1.963	2.484
				3.00	-.264	-.518	-.580	.000
	2.00	1.00	1.00	1.00	1.433	1.321	1.501	1.777
				2.00	-.952	-1.040	-1.152	.000
				3.00	-.481	-.716	-.864	.000
			2.00	1.00	.518	.772	.815	1.054
				2.00	-.451	-.713	-.754	.000
				3.00	-.067	-.262	-.272	.000
		2.00	1.00	1.00	.497	.772	.814	1.043
				2.00	-.340	-.620	-.648	.000
				3.00	-.157	-.407	-.437	.000
			2.00	1.00	.485	.743	.790	1.017
				2.00	-.427	-.691	-.737	.000
				3.00	-.058	-.243	-.252	.000
2.00	1.00	1.00	1.00	1.00	1.138	.722	.871	1.543
				2.00	-1.354	-.961	-1.153	-1.309
				3.00	.217	.252	.315	.699
			2.00	1.00	-.370	-.317	-.362	-.851
				2.00	.516	.459	.527	1.092
				3.00	-.146	-.384	-.413	.000
		2.00	1.00	1.00	2.638	1.141	1.639	2.350
				2.00	-1.093	-.525	-.747	-1.390
				3.00	-1.545	-1.272	-1.940	.000
			2.00	1.00	-.472	-.273	-.344	-.965
				2.00	-.239	-.143	-.182	-.678
				3.00	.710	1.328	1.504	1.574
	2.00	1.00	1.00	1.00	-2.501	-2.304	-2.635	-1.907
				2.00	1.861	1.906	2.174	2.410
				3.00	.640	1.094	1.278	1.429
			2.00	1.00	.505	.779	.822	1.051

			2.00	-.462	-.739	-.781	.000
			3.00	-.043	-.209	-.214	.000
	2.00	1.00	1.00	-.998	-.644	-.810	-1.382
			2.00	.670	.477	.598	1.231
			3.00	.328	.410	.531	.892
		2.00	1.00	.060	.045	.054	.347
			2.00	.102	.080	.096	.457
			3.00	-.161	-.404	-.440	.000

a. Model: Multinomial Logit

b. Design: Constant + Recur1yr123 + Recur1yr123 * HIBP + Recur1yr123 * DM + Recur1yr123 * Age2 + Recur1yr123 * DPaverage

Parameter Estimates[c,d]

Parameter		Estimate	Std. Error	Z	Sig.	95% Confidence Interval	
						Lower Bound	Upper Bound
Constant	[HIBP = 1.00] * [DM = 1.00] * [Age2 = 1.00] * [DPaverage = 1.00]	2.411[a]					
	[HIBP = 1.00] * [DM = 1.00] * [Age2 = 1.00] * [DPaverage = 2.00]	-.030[a]					
	[HIBP = 1.00] * [DM = 1.00] * [Age2 = 2.00] * [DPaverage = 1.00]	.617[a]					
	[HIBP = 1.00] * [DM = 1.00] * [Age2 = 2.00] * [DPaverage = 2.00]	-1.330[a]					
	[HIBP = 1.00] * [DM = 2.00] * [Age2 = 1.00] * [DPaverage = 1.00]	-.731[a]					
	[HIBP = 1.00] * [DM = 2.00] * [Age2 = 1.00] * [DPaverage = 2.00]	-2.700[a]					
	[HIBP = 1.00] * [DM = 2.00] * [Age2 = 2.00] * [DPaverage = 1.00]	-1.852[a]					

[HIBP = 1.00] * [DM = 2.00] * [Age2 = 2.00] * [DPaverage = 2.00]	-2.847[a]					
[HIBP = 2.00] * [DM = 1.00] * [Age2 = 1.00] * [DPaverage = 1.00]	-.244[a]					
[HIBP = 2.00] * [DM = 1.00] * [Age2 = 1.00] * [DPaverage = 2.00]	-1.927[a]					
[HIBP = 2.00] * [DM = 1.00] * [Age2 = 2.00] * [DPaverage = 1.00]	.435[a]					
[HIBP = 2.00] * [DM = 1.00] * [Age2 = 2.00] * [DPaverage = 2.00]	-1.239[a]					
[HIBP = 2.00] * [DM = 2.00] * [Age2 = 1.00] * [DPaverage = 1.00]	-1.021[a]					
[HIBP = 2.00] * [DM = 2.00] * [Age2 = 1.00] * [DPaverage = 2.00]	-3.145[a]					
[HIBP = 2.00] * [DM = 2.00] * [Age2 = 2.00] * [DPaverage = 1.00]	-.398[a]					
[HIBP = 2.00] * [DM = 2.00] * [Age2 = 2.00] * [DPaverage = 2.00]	-1.824[a]					
[Recur1yr123 = 1.00]	4.216	.988	4.269	.000	2.280	6.152
[Recur1yr123 = 2.00]	2.465	1.056	2.335	.020	.396	4.534
[Recur1yr123 = 3.00]	0[b]	.	.	.	.	.
[Recur1yr123 = 1.00] * [HIBP = 1.00]	-.113	.685	-.164	.870	-1.456	1.231
[Recur1yr123 = 1.00] * [HIBP = 2.00]	0[b]	.	.	.	.	.
[Recur1yr123 = 2.00] * [HIBP = 1.00]	-.469	.756	-.620	.535	-1.951	1.013
[Recur1yr123 = 2.00] * [HIBP = 2.00]	0[b]	.	.	.	.	.
[Recur1yr123 = 3.00] * [HIBP = 1.00]	0[b]	.	.	.	.	.
[Recur1yr123 = 3.00] * [HIBP = 2.00]	0[b]	.	.	.	.	.

[Recur1yr123 = 1.00] * [DM = 1.00]	-.008	.819	-.010	.992	-1.613	1.597
[Recur1yr123 = 1.00] * [DM = 2.00]	0[b]	.	.	.	.	.
[Recur1yr123 = 2.00] * [DM = 1.00]	-.051	.901	-.056	.955	-1.816	1.714
[Recur1yr123 = 2.00] * [DM = 2.00]	0[b]	.	.	.	.	.
[Recur1yr123 = 3.00] * [DM = 1.00]	0[b]	.	.	.	.	.
[Recur1yr123 = 3.00] * [DM = 2.00]	0[b]	.	.	.	.	.
[Recur1yr123 = 1.00] * [Age2 = 1.00]	-.157	.610	-.257	.797	-1.352	1.038
[Recur1yr123 = 1.00] * [Age2 = 2.00]	0[b]	.	.	.	.	.
[Recur1yr123 = 2.00] * [Age2 = 1.00]	-.092	.683	-.135	.892	-1.432	1.247
[Recur1yr123 = 2.00] * [Age2 = 2.00]	0[b]	.	.	.	.	.
[Recur1yr123 = 3.00] * [Age2 = 1.00]	0[b]	.	.	.	.	.
[Recur1yr123 = 3.00] * [Age2 = 2.00]	0[b]	.	.	.	.	.
[Recur1yr123 = 1.00] * [DPaverage = 1.00]	-1.334	.761	-1.752	.080	-2.826	.158
[Recur1yr123 = 1.00] * [DPaverage = 2.00]	0[b]	.	.	.	.	.
[Recur1yr123 = 2.00] * [DPaverage = 1.00]	-1.221	.812	-1.504	.133	-2.812	.370
[Recur1yr123 = 2.00] * [DPaverage = 2.00]	0[b]	.	.	.	.	.
[Recur1yr123 = 3.00] * [DPaverage = 1.00]	0[b]	.	.	.	.	.
[Recur1yr123 = 3.00] * [DPaverage = 2.00]	0[b]	.	.	.	.	.

a. Constants are not parameters under the multinomial assumption. Therefore, their standard errors are not calculated.

b. This parameter is set to zero because it is redundant.

c. Model: Multinomial Logit

d. Design: Constant + Recur1yr123 + Recur1yr123 * HIBP + Recur1yr123 * DM + Recur1yr123 * Age2 + Recur1yr123 * DPaverage

Correlations of Parameter Estimates[a,b,c]

	[Recur1yr123 = 1.00]	[Recur1yr123 = 2.00]	[Recur1yr123 = 1.00] * [HIBP = 1.00]	[Recur1yr123 = 2.00] * [HIBP = 1.00]
[Recur1yr123 = 1.00]	1	.910	-.110	-.095
[Recur1yr123 = 2.00]	.910	1	-.099	-.100
[Recur1yr123 = 1.00] * [HIBP = 1.00]	-.110	-.099	1	.867
[Recur1yr123 = 2.00] * [HIBP = 1.00]	-.095	-.100	.867	1
[Recur1yr123 = 1.00] * [DM = 1.00]	-.540	-.484	-.284	-.247
[Recur1yr123 = 2.00] * [DM = 1.00]	-.471	-.554	-.248	-.281
[Recur1yr123 = 1.00] * [Age2 = 1.00]	-.139	-.125	-.436	-.378
[Recur1yr123 = 2.00] * [Age2 = 1.00]	-.120	-.143	-.371	-.441
[Recur1yr123 = 1.00] * [DPaverage = 1.00]	-.611	-.563	-.041	-.035
[Recur1yr123 = 2.00] * [DPaverage = 1.00]	-.566	-.587	-.035	-.044

Correlations of Parameter Estimates[a,b,c]

	[Recur1yr123 = 1.00] * [DM = 1.00]	[Recur1yr123 = 2.00] * [DM = 1.00]	[Recur1yr123 = 1.00] * [Age2 = 1.00]	[Recur1yr123 = 2.00] * [Age2 = 1.00]
[Recur1yr123 = 1.00]	-.540	-.471	-.139	-.120
[Recur1yr123 = 2.00]	-.484	-.554	-.125	-.143
[Recur1yr123 = 1.00] * [HIBP = 1.00]	-.284	-.248	-.436	-.371
[Recur1yr123 = 2.00] * [HIBP = 1.00]	-.247	-.281	-.378	-.441
[Recur1yr123 = 1.00] * [DM = 1.00]	1	.871	-.048	-.040
[Recur1yr123 = 2.00] * [DM = 1.00]	.871	1	-.041	-.051
[Recur1yr123 = 1.00] * [Age2 = 1.00]	-.048	-.041	1	.852
[Recur1yr123 = 2.00] * [Age2 = 1.00]	-.040	-.051	.852	1
[Recur1yr123 = 1.00] * [DPaverage = 1.00]	-.035	-.031	-.059	-.050

Correlations of Parameter Estimates[a,b,c]

	[Recur1yr123 = 1.00] * [DM = 1.00]	[Recur1yr123 = 2.00] * [DM = 1.00]	[Recur1yr123 = 1.00] * [Age2 = 1.00]	[Recur1yr123 = 2.00] * [Age2 = 1.00]
[Recur1yr123 = 1.00]	-.540	-.471	-.139	-.120
[Recur1yr123 = 2.00]	-.484	-.554	-.125	-.143
[Recur1yr123 = 1.00] * [HIBP = 1.00]	-.284	-.248	-.436	-.371
[Recur1yr123 = 2.00] * [HIBP = 1.00]	-.247	-.281	-.378	-.441
[Recur1yr123 = 1.00] * [DM = 1.00]	1	.871	-.048	-.040
[Recur1yr123 = 2.00] * [DM = 1.00]	.871	1	-.041	-.051
[Recur1yr123 = 1.00] * [Age2 = 1.00]	-.048	-.041	1	.852
[Recur1yr123 = 2.00] * [Age2 = 1.00]	-.040	-.051	.852	1
[Recur1yr123 = 1.00] * [DPaverage = 1.00]	-.035	-.031	-.059	-.050
[Recur1yr123 = 2.00] * [DPaverage = 1.00]	-.031	-.036	-.053	-.062

Correlations of Parameter Estimates[a,b,c]

	[Recur1yr123 = 1.00] * [DPaverage = 1.00]	[Recur1yr123 = 2.00] * [DPaverage = 1.00]
[Recur1yr123 = 1.00]	-.611	-.566
[Recur1yr123 = 2.00]	-.563	-.587
[Recur1yr123 = 1.00] * [HIBP = 1.00]	-.041	-.035
[Recur1yr123 = 2.00] * [HIBP = 1.00]	-.035	-.044
[Recur1yr123 = 1.00] * [DM = 1.00]	-.035	-.031
[Recur1yr123 = 2.00] * [DM = 1.00]	-.031	-.036
[Recur1yr123 = 1.00] * [Age2 = 1.00]	-.059	-.053
[Recur1yr123 = 2.00] * [Age2 = 1.00]	-.050	-.062

[Recur1yr123 = 1.00] * [DPaverage = 1.00]	1	.916
[Recur1yr123 = 2.00] * [DPaverage = 1.00]	.916	1

a. Model: Multinomial Logit

b. Design: Constant + Recur1yr123 + Recur1yr123 * HIBP + Recur1yr123 * DM + Recur1yr123 * Age2 + Recur1yr123 * DPaverage

c. Constants and redundant parameters are not displayed.

Covariances of Parameter Estimates[a,b,c]

	[Recur1yr123 = 1.00]	[Recur1yr123 = 2.00]	[Recur1yr123 = 1.00] * [HIBP = 1.00]	[Recur1yr123 = 2.00] * [HIBP = 1.00]
[Recur1yr123 = 1.00]	.976	.948	-.074	-.071
[Recur1yr123 = 2.00]	.948	1.114	-.072	-.080
[Recur1yr123 = 1.00] * [HIBP = 1.00]	-.074	-.072	.470	.449
[Recur1yr123 = 2.00] * [HIBP = 1.00]	-.071	-.080	.449	.572
[Recur1yr123 = 1.00] * [DM = 1.00]	-.437	-.419	-.159	-.153
[Recur1yr123 = 2.00] * [DM = 1.00]	-.419	-.527	-.153	-.192
[Recur1yr123 = 1.00] * [Age2 = 1.00]	-.084	-.081	-.182	-.174
[Recur1yr123 = 2.00] * [Age2 = 1.00]	-.081	-.103	-.174	-.228
[Recur1yr123 = 1.00] * [DPaverage = 1.00]	-.459	-.453	-.021	-.020
[Recur1yr123 = 2.00] * [DPaverage = 1.00]	-.453	-.503	-.019	-.027

Covariances of Parameter Estimates[a,b,c]

	[Recur1yr123 = 1.00] * [DM = 1.00]	[Recur1yr123 = 2.00] * [DM = 1.00]	[Recur1yr123 = 1.00] * [Age2 = 1.00]	[Recur1yr123 = 2.00] * [Age2 = 1.00]
[Recur1yr123 = 1.00]	-.437	-.419	-.084	-.081
[Recur1yr123 = 2.00]	-.419	-.527	-.081	-.103

[Recur1yr123 = 1.00] * [HIBP = 1.00]	-.159	-.153	-.182	-.174
[Recur1yr123 = 2.00] * [HIBP = 1.00]	-.153	-.192	-.174	-.228
[Recur1yr123 = 1.00] * [DM = 1.00]	.671	.642	-.024	-.022
[Recur1yr123 = 2.00] * [DM = 1.00]	.642	.811	-.022	-.032
[Recur1yr123 = 1.00] * [Age2 = 1.00]	-.024	-.022	.372	.355
[Recur1yr123 = 2.00] * [Age2 = 1.00]	-.022	-.032	.355	.467
[Recur1yr123 = 1.00] * [DPaverage = 1.00]	-.022	-.021	-.027	-.026
[Recur1yr123 = 2.00] * [DPaverage = 1.00]	-.021	-.026	-.026	-.034

Covariances of Parameter Estimates[a,b,c]

	[Recur1yr123 = 1.00] * [DPaverage = 1.00]	[Recur1yr123 = 2.00] * [DPaverage = 1.00]
[Recur1yr123 = 1.00]	-.459	-.453
[Recur1yr123 = 2.00]	-.453	-.503
[Recur1yr123 = 1.00] * [HIBP = 1.00]	-.021	-.019
[Recur1yr123 = 2.00] * [HIBP = 1.00]	-.020	-.027
[Recur1yr123 = 1.00] * [DM = 1.00]	-.022	-.021
[Recur1yr123 = 2.00] * [DM = 1.00]	-.021	-.026
[Recur1yr123 = 1.00] * [Age2 = 1.00]	-.027	-.026
[Recur1yr123 = 2.00] * [Age2 = 1.00]	-.026	-.034
[Recur1yr123 = 1.00] * [DPaverage = 1.00]	.580	.566
[Recur1yr123 = 2.00] * [DPaverage = 1.00]	.566	.659

a. Model: Multinomial Logit

b. Design: Constant + Recur1yr123 + Recur1yr123 * HIBP + Recur1yr123 * DM + Recur1yr123 * Age2 + Recur1yr123 * DPaverage

c. Constants and redundant parameters are not displayed.

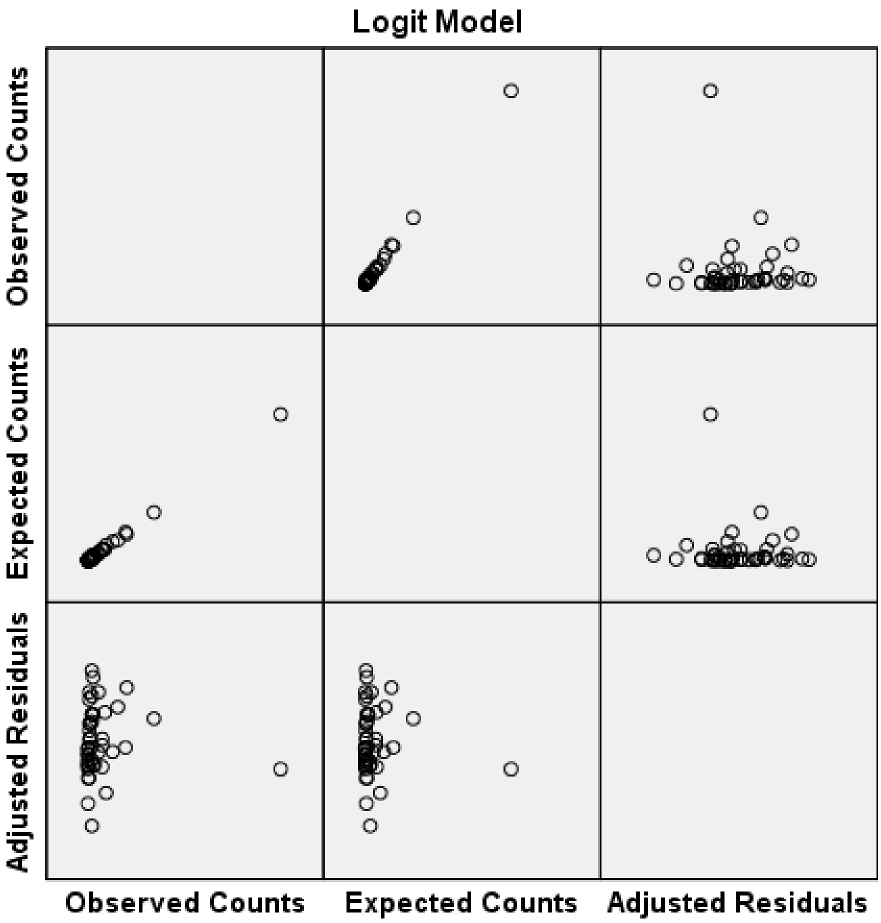

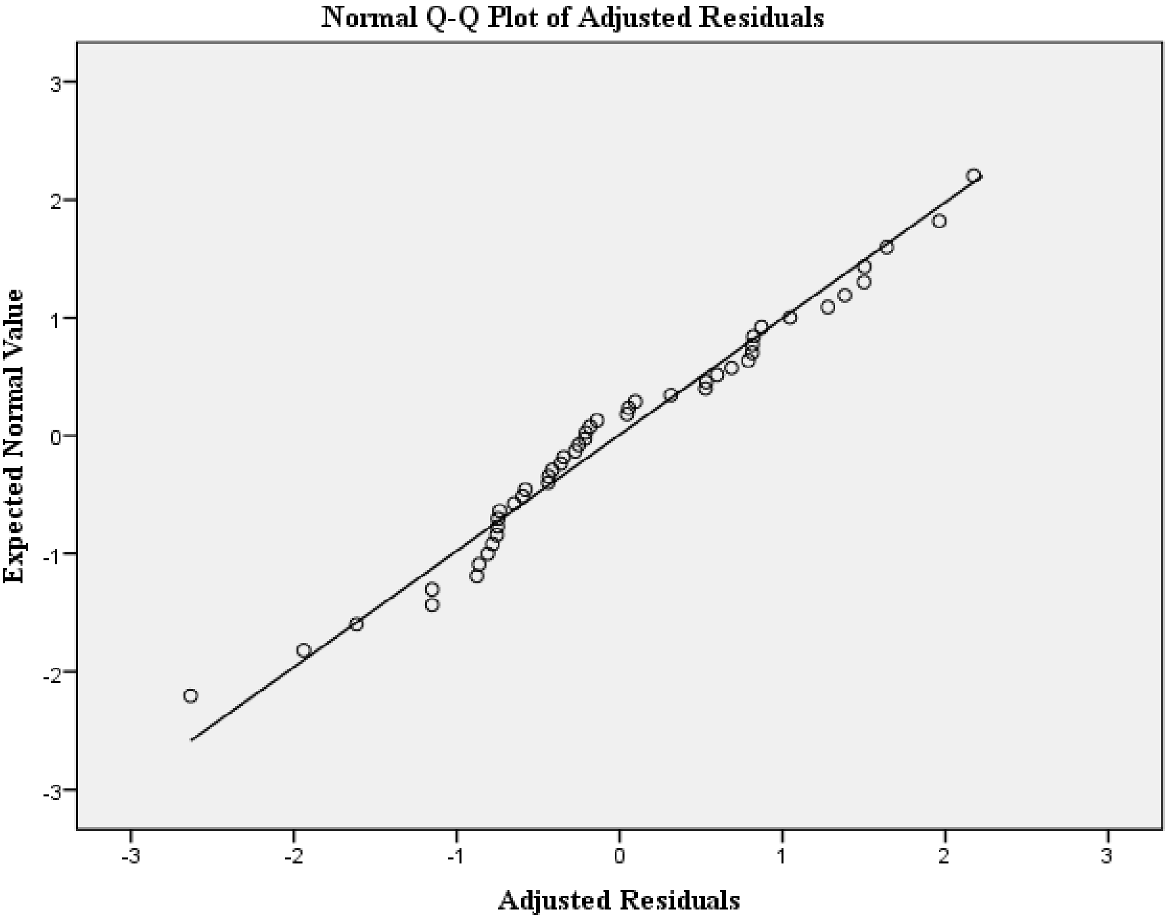
Normal Q-Q Plot of Adjusted Residuals
Expected Normal Value
Adjusted Residuals
3
2
1
0
-1
-2
-3
-3
-2
-1
0
1
2
3

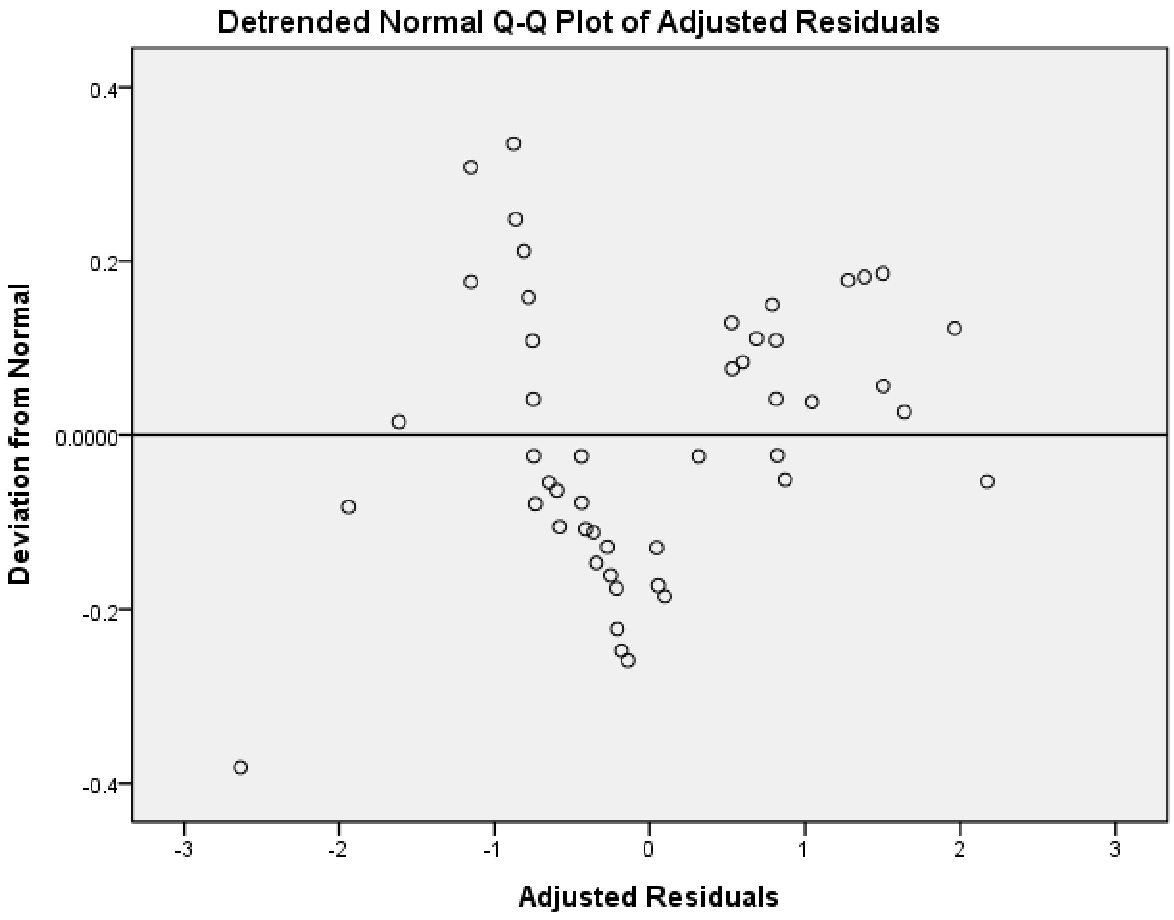
Detrended Normal Q-Q Plot of Adjusted Residuals
Deviation from Normal
0.4
0.2
0.0000
-0.2
-0.4
-3
-2
-1
0
1
2
3
Adjusted Residuals
