# Supplementary material for: Effect of the early diastolic blood pressure response to the head-up tilt test on the recurrence of benign paroxysmal positional vertigo
Source: PLoS One. 2024 May 2;19(5):e0301800. doi: 10.1371/journal.pone.0301800 (PMC11065201; doi:10.1371/journal.pone.0301800)
Supplement: S2 File — (PDF) [file pone.0301800.s002.pdf]

AGE	GENDER	RECUR	HIBP	DM	Supine1	Supine2	Upright1	Upright2	Upright3	AGE2	Supaverae	Upaverag	Dpaverage
51	1	1	1	1	79	77	76	81	81	1	78	79.33	1
51	2	2	2	2	89	90	101	95	89	1	89.5	95	1
64	2	1	2	2	74	69	84	76	75	2	71.5	78.33	1
61	2	1	2	1	78	81	79	78	74	2	79.5	77	2
60	2	1	2	1	107	101	104	107	81	2	104	97.33	2
71	2	1	2	1	70	67	73	74	73	2	68.5	73.33	1
50	2	1	1	1	73	72	74	80	81	1	72.5	78.33	1
47	2	1	1	1	55	55	57	55	56	1	55	56	1
18	1	1	1	1	65	60	66	68	73	1	62.5	69	1
50	2	1	1	1	75	73	86	82	77	1	74	81.67	1
54	2	1	2	1	80	81	90	85	87	1	80.5	87.33	1
42	1	1	1	1	67	64	78	72	71	1	65.5	73.67	1
53	2	1	1	1	87	84	66	75	76	1	85.5	72.33	2
62	2	2	2	1	88	91	86	87	83	2	89.5	85.33	2
60	2	2	1	1	58	54	64	69	65	2	56	66	1
66	2	1	2	1	65	66	81	84	82	2	65.5	82.33	1
58	2	3	2	1	92	87	93	95	90	1	89.5	92.67	1
33	2	2	1	1	69	75	70	67	66	1	72	67.67	2
49	2	1	1	1	83	77	89	86	81	1	80	85.33	1
65	1	1	2	1	83	80	83	86	88	2	81.5	85.67	1
41	2	1	1	1	88	91	84	87	99	1	89.5	90	1
49	2	1	1	1	78	74	75	85	79	1	76	79.67	1
38	2	1	1	1	86	84	90	84	82	1	85	85.33	1
39	1	1	1	1	66	60	73	68	70	1	63	70.33	1
67	2	1	2	2	84	84	81	82	79	2	84	80.67	2
60	2	1	1	1	87	84	91	92	84	2	85.5	89	1
77	2	1	2	1	73	72	86	83	78	2	72.5	82.33	1
50	2	3	1	1	72	71	78	77	80	1	71.5	78.33	1
46	2	2	1	1	83	80	80	86	86	1	81.5	84	1
61	2	1	1	1	86	88	72	84	87	2	87	81	2
45	2	1	1	1	75	73	66	64	64	1	74	64.67	2
30	2	1	1	1	80	65	69	76	71	1	72.5	72	2

37	2	1	1	1	58	56	63	59	57	1	57	59.67	1
55	2	2	2	1	104	104	108	104	102	1	104	104.67	1
44	2	3	1	1	78	79	91	90	88	1	78.5	89.67	1
51	2	1	1	1	90	84	90	91	88	1	87	89.67	1
61	2	1	2	1	84	82	77	74	78	2	83	76.33	2
56	2	1	1	1	60	57	67	65	64	1	58.5	65.33	1
60	2	3	1	1	80	77	81	82	78	2	78.5	80.33	1
75	2	1	2	1	85	81	87	91	85	2	83	87.67	1
36	2	2	1	1	58	54	65	62	60	1	56	62.33	1
30	1	1	1	1	84	84	84	89	85	1	84	86	1
54	2	2	1	1	83	76	83	84	81	1	79.5	82.67	1
60	2	1	2	1	86	71	84	83	77	2	78.5	81.33	1
24	2	1	1	1	59	54	70	68	62	1	56.5	66.67	1
73	1	1	1	1	70	69	49	80	81	2	69.5	70	1
43	2	1	1	1	75	72	66	74	74	1	73.5	71.33	2
27	1	1	1	1	74	67	78	76	78	1	70.5	77.33	1
47	2	1	1	1	94	89	94	88	89	1	91.5	90.33	2
35	2	1	1	1	64	59	63	65	59	1	61.5	62.33	1
78	1	1	2	1	83	82	82	80	76	2	82.5	79.33	2
42	1	1	2	1	71	73	73	69	66	1	72	69.33	2
61	1	1	2	2	101	99	101	96	95	2	100	97.33	2
60	2	1	2	2	76	77	80	77	80	2	76.5	79	1
52	2	1	2	2	85	82	85	92	87	1	83.5	88	1
56	2	1	1	1	83	84	84	85	89	1	83.5	86	1
73	2	1	1	1	84	83	91	87	86	2	83.5	88	1
74	2	2	2	2	69	67	66	65	65	2	68	65.33	2
36	2	1	1	1	58	61	68	59	67	1	59.5	64.67	1
70	2	1	2	1	85	84	74	81	82	2	84.5	79	2
60	2	1	2	1	73	77	82	80	84	2	75	82	1
65	2	1	2	1	85	79	91	92	86	2	82	89.67	1
40	2	1	1	1	61	58	67	68	64	1	59.5	66.33	1
66	1	3	2	1	80	76	79	79	78	2	78	78.67	1
76	2	1	1	1	69	68	57	61	67	2	68.5	61.67	2

56	2	1	1	1	96	94	96	94	94	1	95	94.67	2
75	2	1	1	1	85	84	86	82	86	2	84.5	84.67	1
41	1	1	1	1	97	96	109	108	111	1	96.5	109.33	1
51	1	1	2	2	85	86	99	100	96	1	85.5	98.33	1
38	2	2	1	1	80	85	84	87	80	1	82.5	83.67	1
53	1	1	2	1	101	100	99	107	100	1	100.5	102	1
20	1	1	1	1	82	75	82	83	78	1	78.5	81	1
61	2	1	1	1	74	79	82	83	88	2	76.5	84.33	1
58	2	1	1	1	81	75	76	77	78	1	78	77	2
42	1	1	1	1	96	94	100	93	86	1	95	93	2
51	2	1	1	1	92	96	88	100	93	1	94	93.67	2
50	2	1	1	2	83	86	97	88	96	1	84.5	93.67	1
52	2	1	1	2	91	82	95	91	92	1	86.5	92.67	1
24	1	1	1	1	98	93	99	100	101	1	95.5	100	1
34	2	1	1	1	68	77	73	77	75	1	72.5	75	1
28	2	2	1	1	76	76	83	83	84	1	76	83.33	1
60	1	1	2	2	95	97	97	95	94	2	96	95.33	2
32	1	1	1	1	101	99	99	98	99	1	100	98.67	2
27	1	1	1	1	73	67	71	71	71	1	70	71	1
62	2	1	1	1	98	98	102	95	96	2	98	97.67	2
48	2	1	2	2	81	80	72	70	71	1	80.5	71	2
69	2	2	1	1	85	84	75	77	79	2	84.5	77	2
57	2	1	1	1	89	83	96	99	91	1	86	95.33	1
56	2	1	2	2	83	83	83	90	90	1	83	87.67	1
49	1	1	1	1	102	103	112	110	115	1	102.5	112.33	1
67	2	1	2	2	74	71	84	80	79	2	72.5	81	1
26	2	1	1	1	79	73	85	76	82	1	76	81	1
68	2	1	2	2	94	86	92	93	89	2	90	91.33	1
32	1	1	1	1	81	78	84	82	91	1	79.5	85.67	1
53	1	1	1	1	97	96	92	97	94	1	96.5	94.33	2
54	2	1	2	1	79	74	86	81	76	1	76.5	81	1
58	2	2	2	2	77	78	86	84	83	1	77.5	84.33	1
35	2	3	1	1	65	63	84	81	84	1	64	83	1

44	2	1	1	1	76	77	85	82	79	1	76.5	82	1
19	2	3	1	1	64	58	67	55	68	1	61	63.33	1
55	2	1	1	1	105	95	109	99	101	1	100	103	1
22	2	1	1	1	72	67	84	84	82	1	69.5	83.33	1
33	2	1	1	1	75	73	74	74	79	1	74	75.67	1
60	1	1	2	2	94	86	74	92	86	2	90	84	2
64	2	1	1	1	77	72	83	82	83	2	74.5	82.67	1
76	2	1	1	1	64	62	65	66	63	2	63	64.67	1
41	2	1	1	1	78	70	85	75	75	1	74	78.33	1
60	2	1	1	1	74	74	78	78	76	2	74	77.33	1
30	2	2	1	1	60	56	65	54	66	1	58	61.67	1
53	1	1	2	1	78	77	87	88	87	1	77.5	87.33	1
69	2	1	1	1	81	78	85	84	84	2	79.5	84.33	1
46	1	1	1	1	87	83	92	87	81	1	85	86.67	1
54	2	1	2	1	82	88	102	91	92	1	85	95	1
43	2	1	1	1	82	79	83	84	83	1	80.5	83.33	1
36	1	1	1	1	95	93	101	99	91	1	94	97	1
46	2	1	1	1	67	60	67	65	67	1	63.5	66.33	1
63	1	1	2	1	99	94	95	96	97	2	96.5	96	2
68	2	1	2	2	64	63	73	75	70	2	63.5	72.67	1
21	2	1	1	1	54	49	62	63	63	1	51.5	62.67	1
31	1	1	1	1	69	70	83	84	86	1	69.5	84.33	1
57	1	1	2	2	83	80	73	77	78	1	81.5	76	2
73	2	1	2	2	73	67	72	68	68	2	70	69.33	2
74	1	1	2	2	84	86	86	89	90	2	85	88.33	1
37	2	1	1	1	81	75	82	88	82	1	78	84	1
21	1	1	1	1	77	74	79	77	76	1	75.5	77.33	1
78	2	2	2	2	60	58	64	79	82	2	59	75	1
29	1	1	1	1	68	62	71	66	66	1	65	67.67	1
63	2	2	2	2	73	71	82	84	82	2	72	82.67	1
38	2	2	1	1	64	66	75	70	62	1	65	69	1
29	2	2	1	1	77	67	71	78	76	1	72	75	1
39	2	1	1	1	84	81	86	88	86	1	82.5	86.67	1

51	2	1	2	1	90	85	93	89	90	1	87.5	90.67	1
77	2	1	2	1	81	81	93	87	77	2	81	85.67	1
75	2	2	2	1	65	65	56	58	65	2	65	59.67	2
66	2	1	1	1	85	82	86	92	94	2	83.5	90.67	1
61	1	1	2	2	84	83	78	84	84	2	83.5	82	2
72	2	1	2	1	79	77	81	77	76	2	78	78	2
58	2	1	1	1	89	85	93	97	99	1	87	96.33	1
77	2	2	2	2	81	75	86	90	88	2	78	88	1
78	2	1	2	2	86	89	90	87	96	2	87.5	91	1
38	2	1	1	1	73	72	72	75	73	1	72.5	73.33	1
53	2	1	1	1	64	66	74	78	78	1	65	76.67	1
38	2	1	1	1	80	78	83	85	86	1	79	84.67	1
56	2	1	1	1	80	80	96	91	94	1	80	93.67	1
21	2	2	1	1	71	67	70	74	75	1	69	73	1
62	2	2	1	1	77	71	71	73	73	2	74	72.33	2
69	1	1	1	1	84	83	91	86	89	2	83.5	88.67	1
36	2	1	1	1	69	69	72	70	69	1	69	70.33	1
60	2	3	1	1	79	79	82	81	80	2	79	81	1
68	2	1	1	1	66	68	69	73	73	2	67	71.67	1
23	2	1	1	1	67	68	70	77	66	1	67.5	71	1
58	1	1	1	1	94	85	94	96	92	1	89.5	94	1
50	1	1	2	1	80	80	77	77	76	1	80	76.67	2
46	2	3	1	1	86	82	87	87	84	1	84	86	1
50	1	1	1	1	80	79	85	90	78	1	79.5	84.33	1
43	2	1	1	1	70	67	69	74	66	1	68.5	69.67	1
73	2	1	2	2	79	80	79	83	82	2	79.5	81.33	1
73	1	1	2	2	77	78	67	74	79	2	77.5	73.33	2
47	1	1	1	1	69	69	70	70	74	1	69	71.33	1
38	1	2	1	1	86	82	89	87	90	1	84	88.67	1
27	2	1	1	1	75	68	74	81	79	1	71.5	78	1
30	2	1	1	1	56	53	60	61	58	1	54.5	59.67	1
69	2	2	1	1	83	85	82	89	85	2	84	85.33	1
36	2	1	1	1	58	52	58	61	57	1	55	58.67	1

25	2	1	1	1	80	77	84	78	80	1	78.5	80.67	1
33	1	1	1	1	68	63	71	76	68	1	65.5	71.67	1
52	2	3	1	1	83	84	88	87	89	1	83.5	88	1
61	2	2	1	1	80	78	79	69	75	2	79	74.33	2
45	2	1	1	1	83	79	92	88	89	1	81	89.67	1
52	1	1	1	1	75	74	80	80	79	1	74.5	79.67	1
47	2	1	1	2	74	72	73	73	68	1	73	71.33	2
34	2	2	1	1	83	78	77	71	72	1	80.5	73.33	2
74	1	1	2	1	79	78	78	82	73	2	78.5	77.67	2
54	2	2	1	1	88	85	84	64	79	1	86.5	75.67	2
52	2	3	2	1	87	84	89	84	84	1	85.5	85.67	1
55	2	1	1	1	67	69	76	72	72	1	68	73.33	1
72	1	1	1	1	73	69	65	67	69	2	71	67	2
42	2	1	1	1	85	81	74	83	86	1	83	81	2
47	2	1	1	1	89	74	77	75	75	1	81.5	75.67	2
61	2	1	2	2	96	86	92	97	90	2	91	93	1
37	2	1	1	1	76	71	82	82	84	1	73.5	82.67	1
79	1	1	1	2	58	62	35	50	58	2	60	47.67	2
46	1	1	1	1	77	82	68	79	74	1	79.5	73.67	2
46	2	1	1	1	54	55	55	58	58	1	54.5	57	1
84	2	1	2	1	72	74	71	69	74	2	73	71.33	2
70	1	1	2	1	89	82	100	106	105	2	85.5	103.67	1
55	2	1	1	1	60	58	65	62	62	1	59	63	1
18	2	2	1	1	59	54	53	56	53	1	56.5	54	2
49	1	1	1	1	83	86	91	89	89	1	84.5	89.67	1
73	2	2	2	1	102	92	92	95	90	2	97	92.33	2
51	1	1	1	1	83	85	91	97	98	1	84	95.33	1
80	2	1	1	1	89	96	105	90	89	2	92.5	94.67	1
47	2	1	1	1	82	80	91	94	89	1	81	91.33	1
49	1	1	1	2	78	75	88	90	85	1	76.5	87.67	1
40	2	2	1	1	62	60	65	67	67	1	61	66.33	1
36	2	1	1	1	86	81	83	86	87	1	83.5	85.33	1
33	1	1	1	1	82	83	85	88	85	1	82.5	86	1

74	1	1	2	1	69	68	73	71	69	2	68.5	71	1
43	1	2	1	1	110	110	118	115	114	1	110	115.67	1
41	2	1	1	1	54	54	55	58	56	1	54	56.33	1
32	2	1	1	1	77	68	88	72	74	1	72.5	78	1
44	2	1	1	1	102	99	92	101	103	1	100.5	98.67	2
10	2	3	1	1	52	54	54	52	51	1	53	52.33	2
56	1	2	1	1	92	88	95	93	91	1	90	93	1
68	2	1	2	2	80	81	77	74	76	2	80.5	75.67	2
16	2	1	1	1	56	57	57	53	56	1	56.5	55.33	2
54	2	1	1	1	79	75	83	82	85	1	77	83.33	1
56	1	1	2	1	69	70	68	65	62	1	69.5	65	2
67	1	1	2	1	76	74	72	68	67	2	75	69	2
44	2	1	1	1	74	69	77	68	80	1	71.5	75	1
30	2	1	1	1	64	63	78	66	70	1	63.5	71.33	1
53	2	1	1	1	91	87	89	86	85	1	89	86.67	2
19	2	1	1	1	62	57	54	55	59	1	59.5	56	2
54	2	1	2	1	78	78	87	80	76	1	78	81	1
63	2	1	1	1	89	86	92	99	94	2	87.5	95	1
83	1	1	2	1	86	85	85	86	84	2	85.5	85	2
80	2	1	2	1	84	80	89	92	86	2	82	89	1
56	2	1	1	1	77	77	85	84	77	1	77	82	1
32	2	1	1	1	62	59	58	64	59	1	60.5	60.33	2
44	1	1	2	2	88	89	83	86	84	1	88.5	84.33	2
68	2	1	2	1	64	60	59	59	59	2	62	59	2
57	2	1	1	2	75	72	73	73	70	1	73.5	72	2
70	2	1	2	1	87	82	90	87	89	2	84.5	88.67	1
65	2	1	1	2	86	84	92	93	91	2	85	92	1
35	2	1	1	1	66	67	76	74	68	1	66.5	72.67	1
71	2	1	2	1	70	64	73	76	75	2	67	74.67	1
74	2	1	2	1	65	64	72	72	73	2	64.5	72.33	1
69	2	1	1	1	78	80	83	122	115	2	79	106.67	1
48	1	1	1	1	75	75	80	79	75	1	75	78	1
26	1	1	1	1	62	58	80	76	78	1	60	78	1

42	2	1	1	1	66	62	74	73	71	1	64	72.67	1
18	2	1	1	1	58	61	63	60	66	1	59.5	63	1
43	2	1	1	1	77	74	75	73	72	1	75.5	73.33	2
38	1	1	1	1	70	69	76	83	81	1	69.5	80	1
42	2	1	1	1	86	86	71	81	74	1	86	75.33	2
65	2	1	1	1	80	78	87	86	81	2	79	84.67	1
49	2	1	2	1	83	78	78	79	72	1	80.5	76.33	2
78	1	1	2	1	64	62	69	74	77	2	63	73.33	1
26	2	1	1	1	71	64	68	70	71	1	67.5	69.67	1
78	2	1	1	1	85	80	66	81	65	2	82.5	70.67	2
51	1	1	1	1	74	74	79	87	81	1	74	82.33	1
62	2	1	1	1	68	67	72	75	74	2	67.5	73.67	1
65	2	1	1	1	72	76	56	71	67	2	74	64.67	2
61	2	1	2	1	68	70	72	70	75	2	69	72.33	1
34	2	3	1	1	61	57	68	68	69	1	59	68.33	1
31	2	1	1	1	90	90	90	86	87	1	90	87.67	2
51	2	1	1	1	89	80	87	85	81	1	84.5	84.33	2
61	2	1	2	1	120	117	121	111	117	2	118.5	116.33	2
39	2	1	1	1	83	85	75	78	81	1	84	78	2
23	2	1	1	1	64	61	55	62	65	1	62.5	60.67	2
67	1	1	1	1	82	84	87	86	87	2	83	86.67	1
63	2	2	2	1	74	76	77	74	77	2	75	76	1
72	2	1	1	1	83	80	85	84	84	2	81.5	84.33	1
55	2	1	1	1	76	79	80	84	83	1	77.5	82.33	1
65	1	1	1	2	72	71	56	68	61	2	71.5	61.67	2
38	1	2	1	1	76	73	84	86	85	1	74.5	85	1
69	1	1	2	1	90	85	86	89	90	2	87.5	88.33	1
68	1	1	2	2	65	66	68	72	72	2	65.5	70.67	1
30	2	3	1	1	64	64	76	78	72	1	64	75.33	1
28	1	1	1	1	90	87	86	86	86	1	88.5	86	2
63	1	1	1	1	64	67	65	67	68	2	65.5	66.67	1
47	1	1	1	1	83	76	89	92	87	1	79.5	89.33	1
55	2	1	2	1	76	70	80	90	92	1	73	87.33	1

20	2	1	1	1	70	68	77	76	72	1	69	75	1
64	2	1	1	1	85	84	85	86	90	2	84.5	87	1
43	2	3	1	1	64	68	75	78	71	1	66	74.67	1
37	2	1	1	1	59	55	53	54	55	1	57	54	2
39	2	1	1	1	66	69	67	65	77	1	67.5	69.67	1
34	2	1	1	1	72	69	77	75	77	1	70.5	76.33	1
37	1	1	1	1	77	72	80	79	79	1	74.5	79.33	1
77	2	1	1	1	80	85	79	85	84	2	82.5	82.67	1
40	2	1	1	1	62	63	63	56	69	1	62.5	62.67	1
62	1	1	2	1	87	90	88	86	86	2	88.5	86.67	2
49	1	2	2	1	71	66	67	65	64	1	68.5	65.33	2
53	1	2	2	1	98	93	86	91	88	1	95.5	88.33	2
48	2	1	1	1	90	83	90	90	82	1	86.5	87.33	1
59	2	1	1	1	76	74	71	74	69	1	75	71.33	2
42	2	1	2	1	78	82	83	85	81	1	80	83	1
35	2	1	1	1	73	68	70	71	75	1	70.5	72	1
63	2	1	2	1	89	85	88	92	90	2	87	90	1
45	2	1	1	2	70	69	79	84	66	1	69.5	76.33	1
40	2	1	1	1	96	95	93	100	100	1	95.5	97.67	1
41	2	1	1	1	83	83	72	84	79	1	83	78.33	2
52	2	1	1	1	81	81	61	73	73	1	81	69	2
47	2	1	1	1	86	87	88	88	82	1	86.5	86	2
24	1	1	1	1	89	91	85	84	83	1	90	84	2
40	2	1	1	1	99	94	98	100	104	1	96.5	100.67	1
36	1	3	1	1	84	80	88	97	83	1	82	89.33	1
64	2	1	1	1	85	96	96	97	94	2	90.5	95.67	1
77	1	1	1	1	82	78	67	72	67	2	80	68.67	2
49	2	1	1	1	79	80	76	79	87	1	79.5	80.67	1
21	2	1	1	1	64	54	71	79	71	1	59	73.67	1
59	2	3	1	1	86	86	91	92	91	1	86	91.33	1
66	2	1	1	1	89	88	81	83	83	2	88.5	82.33	2
83	2	1	2	1	71	68	77	79	84	2	69.5	80	1
30	2	1	1	1	86	84	86	86	83	1	85	85	2

57	2	1	1	2	76	81	69	73	72	1	78.5	71.33	2
36	2	1	1	1	68	60	58	69	61	1	64	62.67	2
63	2	2	2	1	90	94	99	97	90	2	92	95.33	1
34	1	1	1	1	69	61	64	60	62	1	65	62	2
36	1	1	1	1	102	103	111	105	105	1	102.5	107	1
34	2	1	1	1	83	77	71	84	75	1	80	76.67	2
59	2	1	2	1	89	87	82	84	89	1	88	85	2
26	2	1	1	1	75	73	85	77	86	1	74	82.67	1
30	2	1	1	1	74	70	72	71	63	1	72	68.67	2
52	2	1	2	1	92	92	88	87	85	1	92	86.67	2
47	2	1	1	1	88	89	90	88	89	1	88.5	89	1
38	1	2	1	1	91	76	97	79	89	1	83.5	88.33	1
57	2	1	2	1	98	101	99	94	90	1	99.5	94.33	2
68	2	1	2	1	84	83	88	86	81	2	83.5	85	1
16	1	1	1	1	71	74	79	71	85	1	72.5	78.33	1
48	2	1	1	1	83	82	76	81	78	1	82.5	78.33	2
61	2	1	1	1	83	78	81	82	83	2	80.5	82	1
72	2	1	2	2	85	87	83	82	85	2	86	83.33	2
55	1	1	1	1	92	89	81	87	91	1	90.5	86.33	2
40	1	1	1	1	102	94	106	106	105	1	98	105.67	1
48	2	1	1	1	68	63	70	70	66	1	65.5	68.67	1
53	1	1	1	1	81	79	84	82	82	1	80	82.67	1
52	2	2	1	1	58	59	70	65	67	1	58.5	67.33	1
39	2	1	1	1	80	78	69	75	70	1	79	71.33	2
87	2	2	2	2	91	86	84	86	83	2	88.5	84.33	2
37	1	1	1	1	86	79	86	88	87	1	82.5	87	1
61	1	1	2	1	100	102	105	105	106	2	101	105.33	1
65	2	1	1	1	94	101	104	86	97	2	97.5	95.67	2
47	1	1	1	2	105	99	108	103	102	1	102	104.33	1
48	2	1	1	1	69	66	65	67	60	1	67.5	64	2
40	1	1	1	1	80	75	83	77	76	1	77.5	78.67	1
66	2	2	1	1	77	79	84	83	77	2	78	81.33	1
66	2	1	2	1	81	79	82	80	85	2	80	82.33	1

45	2	1	2	1	100	93	99	93	91	1	96.5	94.33	2
77	2	1	2	1	103	97	100	91	77	2	100	89.33	2
53	2	1	1	1	74	71	80	71	84	1	72.5	78.33	1
31	2	1	1	1	74	75	83	84	84	1	74.5	83.67	1
69	1	1	2	1	88	86	73	81	71	2	87	75	2
46	2	1	1	1	78	75	89	86	91	1	76.5	88.67	1
38	2	1	1	1	91	87	92	93	83	1	89	89.33	1
36	2	1	1	1	72	68	67	69	72	1	70	69.33	2
74	2	1	2	1	66	67	74	79	78	2	66.5	77	1
38	2	1	1	1	64	64	63	66	57	1	64	62	2
53	2	1	1	1	74	73	86	86	85	1	73.5	85.67	1
48	1	2	2	2	88	84	100	97	93	1	86	96.67	1
72	2	1	2	1	67	66	63	69	64	2	66.5	65.33	2
52	1	1	1	1	97	95	101	98	98	1	96	99	1
35	2	1	1	1	62	61	65	69	66	1	61.5	66.67	1
53	2	1	1	1	81	78	83	81	87	1	79.5	83.67	1
59	2	1	1	2	75	82	81	80	81	1	78.5	80.67	1
34	1	1	1	1	83	76	81	79	83	1	79.5	81	1
40	1	1	1	1	79	75	81	84	82	1	77	82.33	1
21	1	1	1	1	67	64	74	71	72	1	65.5	72.33	1
61	2	1	2	1	101	96	105	101	104	2	98.5	103.33	1
84	2	1	1	1	82	86	79	83	81	2	84	81	2
61	2	1	1	1	79	79	80	82	82	2	79	81.33	1
16	2	1	1	1	58	57	57	58	60	1	57.5	58.33	1
83	2	1	2	1	77	74	75	79	77	2	75.5	77	1
62	2	3	2	1	98	96	93	94	85	2	97	90.67	2
86	2	1	2	1	86	89	88	94	88	2	87.5	90	1
39	2	1	1	1	65	66	63	64	74	1	65.5	67	1
60	2	3	1	1	84	85	83	91	88	2	84.5	87.33	1
36	1	1	1	1	77	81	85	81	82	1	79	82.67	1
57	1	1	1	2	96	90	94	95	93	1	93	94	1
78	1	1	2	2	62	61	54	52	51	2	61.5	52.33	2
72	2	1	2	1	83	82	83	85	80	2	82.5	82.67	1

10	2	2	1	1	50	51	52	60	48	1	50.5	53.33	1
51	1	1	1	1	64	71	81	76	68	1	67.5	75	1
23	2	1	1	1	93	85	90	89	90	1	89	89.67	1
50	2	1	1	1	84	83	78	75	76	1	83.5	76.33	2
64	2	2	2	1	86	86	89	97	85	2	86	90.33	1
67	2	1	1	1	93	86	88	87	86	2	89.5	87	2
32	2	1	1	1	54	54	60	64	55	1	54	59.67	1
64	1	1	1	1	91	84	86	87	87	2	87.5	86.67	2
65	2	2	2	1	87	82	94	85	87	2	84.5	88.67	1
56	2	1	2	1	98	96	102	102	107	1	97	103.67	1
76	1	1	1	1	76	73	73	67	71	2	74.5	70.33	2
68	2	1	1	1	69	68	76	76	73	2	68.5	75	1
40	1	1	1	1	65	65	70	65	69	1	65	68	1
38	2	1	1	1	71	72	76	75	70	1	71.5	73.67	1
62	2	1	1	1	70	68	78	73	76	2	69	75.67	1
61	1	1	1	2	76	75	72	70	67	2	75.5	69.67	2
25	1	1	1	1	77	74	81	81	78	1	75.5	80	1
33	2	1	1	1	66	60	56	63	48	1	63	55.67	2
48	1	1	1	1	98	90	88	93	83	1	94	88	2
59	2	1	2	1	84	79	86	84	83	1	81.5	84.33	1
40	2	1	1	1	74	75	82	84	83	1	74.5	83	1
36	2	1	1	1	58	58	52	57	56	1	58	55	2
64	1	1	1	1	89	86	86	90	92	2	87.5	89.33	1
49	2	1	2	1	45	43	70	70	67	1	44	69	1
34	2	1	1	1	77	73	72	74	72	1	75	72.67	2
49	2	1	1	1	117	109	120	120	121	1	113	120.33	1
35	2	1	1	1	85	85	83	83	82	1	85	82.67	2
53	2	1	1	1	82	82	85	87	91	1	82	87.67	1
34	1	1	1	1	72	68	80	81	66	1	70	75.67	1
47	1	2	1	1	97	94	102	99	98	1	95.5	99.67	1
24	1	1	1	1	78	72	76	75	79	1	75	76.67	1
46	1	1	1	1	93	76	87	90	93	1	84.5	90	1
55	2	2	1	1	79	78	86	87	82	1	78.5	85	1

77	2	1	2	1	78	77	78	74	79	2	77.5	77	2
16	2	1	1	1	82	83	87	88	85	1	82.5	86.67	1
39	1	1	2	1	75	73	75	75	78	1	74	76	1
12	1	1	1	1	60	59	60	55	65	1	59.5	60	1
16	2	1	1	1	56	47	56	65	58	1	51.5	59.67	1
44	1	1	1	1	77	78	85	89	88	1	77.5	87.33	1
42	2	1	1	1	74	76	83	87	89	1	75	86.33	1
72	2	1	2	1	82	71	79	66	75	2	76.5	73.33	2
64	1	1	2	2	64	78	81	80	79	2	71	80	1
41	2	1	1	2	77	79	86	84	83	1	78	84.33	1
46	2	2	1	1	76	80	85	84	82	1	78	83.67	1
57	2	1	1	1	72	70	74	78	74	1	71	75.33	1
59	2	1	1	1	68	69	75	70	66	1	68.5	70.33	1
49	2	1	1	1	71	73	80	82	80	1	72	80.67	1
61	2	1	2	1	80	75	84	82	81	2	77.5	82.33	1
36	1	2	1	1	75	77	80	81	79	1	76	80	1
50	2	1	1	1	61	54	61	63	58	1	57.5	60.67	1
67	2	1	2	2	68	67	68	60	54	2	67.5	60.67	2
53	1	1	1	1	84	79	90	90	93	1	81.5	91	1
80	2	1	1	2	80	79	84	83	83	2	79.5	83.33	1
75	1	1	2	1	95	104	108	103	114	2	99.5	108.33	1
39	1	1	1	1	67	65	76	78	84	1	66	79.33	1
44	2	1	1	1	85	83	86	85	89	1	84	86.67	1
30	2	1	1	1	57	54	64	66	62	1	55.5	64	1
64	2	1	1	1	102	92	88	86	79	2	97	84.33	2
61	2	2	1	1	69	72	66	68	70	2	70.5	68	2
43	1	2	1	1	78	73	81	79	86	1	75.5	82	1
53	2	1	1	1	68	66	70	67	66	1	67	67.67	1
50	2	2	1	1	58	59	67	72	68	1	58.5	69	1
34	1	1	1	2	62	59	60	60	56	1	60.5	58.67	2
61	2	1	1	1	86	80	87	92	99	2	83	92.67	1
58	2	1	1	1	79	81	75	78	82	1	80	78.33	2
56	2	1	1	1	68	73	78	78	76	1	70.5	77.33	1

66	2	1	1	2	89	89	91	87	87	2	89	88.33	2
24	2	2	1	1	84	80	80	86	79	1	82	81.67	2
61	2	1	1	2	75	75	83	82	83	2	75	82.67	1
54	2	1	1	1	80	79	74	80	75	1	79.5	76.33	2
